# Comparison of luteal support protocols in fresh IVF/ICSI cycles: a network meta-analysis

**DOI:** 10.1038/s41598-024-64804-z

**Published:** 2024-06-24

**Authors:** Stavroula L. Kastora, Grigoria Gkova, Konstantinos Stavridis, Neerujah Balachandren, Athanasios Kastoras, Andreas Karakatsanis, Dimitrios Mavrelos

**Affiliations:** 1 UCL EGA Institute for Women’s Health, University College London, Medical School Building, Room G15, 86-96 Chenies Mews, 74 Huntley Street, London, WC1E 6HX UK; 2 Department of Obstetrics and Gynaecology, Barnet Hospital, Royal Free London NHS Foundation Trust, London, UK; 3 2nd Department of Obstetrics and Gynaecology, “Aretaieion” University Hospital, Athens, Greece; 4 Reproductive Medicine Unit, “Leto” Maternity Hospital, Mouson Str. 7-13, 11524 Athens, Greece; 5 Department of Surgical Sciences, Faculty of Medicine, Uppsala University, Uppsala, Sweden; 6 Section for Breast Surgery, Department of Surgery, Uppsala University Hospital (Akademiska), Uppsala, Sweden

**Keywords:** Network meta-analysis, Progesterone, Fertility, Luteal support, Clinical pregnancy, Live birth, Miscarriage, Multiple pregnancies, OHSS, Infertility, Outcomes research

## Abstract

Despite the proven superiority of various luteal phase support protocols (LPS) over placebo in view of improved pregnancy rates in fresh cycles of IVF (in vitro fertilization) and ICSI (intracytoplasmic sperm injection) cycles, there is ongoing controversy over specific LPS protocol selection, dosage, and duration. The aim of the present study was to identify the optimal LPS under six core aspects of ART success, clinical pregnancy, live birth as primary outcomes and biochemical pregnancy, miscarriage, multiple pregnancy, ovarian hyperstimulation syndrome (OHSS) events as secondary outcomes. Twelve databases, namely Embase (OVID), MEDLINE (R) (OVID), GlobalHealth (Archive), GlobalHealth, Health and Psychosocial Instruments, Maternity & Infant Care Database (MIDIRS), APA PsycTests, ClinicalTrials.gov, HMIC Health Management Information Consortium, CENTRAL, Web of Science, Scopus and two prospective registers, MedRxiv, Research Square were searched from inception to Aug.1st, 2023, (PROSPERO Registration: CRD42022358986). Only Randomised Controlled Trials (RCTs) were included. Bayesian network meta-analysis (NMA) model was employed for outcome analysis, presenting fixed effects, odds ratios (ORs) with 95% credibility intervals (CrIs). Vaginal Progesterone (VP) was considered the reference LPS given its’ clinical relevance. Seventy-six RCTs, comparing 22 interventions, and including 26,536 participants were included in the present NMA. Overall CiNeMa risk of bias was deemed moderate, and network inconsistency per outcome was deemed low (Multiple pregnancy *χ*^2^: 0.11, OHSS *χ*^2^: 0.26), moderate (Clinical Pregnancy: *χ*^2^: 7.02, Live birth *χ*^2^: 10.95, Biochemical pregnancy: χ^2^: 6.60, Miscarriage: *χ*^2^: 11.305). Combinatorial regimens, with subcutaneous GnRH-a (SCGnRH-a) on a vaginal progesterone base and oral oestrogen (OE) appeared to overall improve clinical pregnancy events; VP + OE + SCGnRH-a [OR 1.57 (95% CrI 1.11 to 2.22)], VP + SCGnRH-a [OR 1.28 (95% CrI 1.05 to 1.55)] as well as live pregnancy events, VP + OE + SCGnRH-a [OR 8.81 (95% CrI 2.35 to 39.1)], VP + SCGnRH-a [OR 1.76 (95% CrI 1.45 to 2.15)]. Equally, the progesterone free LPS, intramuscular human chorionic gonadotrophin, [OR 9.67 (95% CrI 2.34, 73.2)] was also found to increase live birth events, however was also associated with an increased probability of ovarian hyperstimulation, [OR 1.64 (95% CrI 0.75, 3.71)]. The combination of intramuscular and vaginal progesterone was associated with higher multiple pregnancy events, [OR 7.09 (95% CrI 2.49, 31.)]. Of all LPS protocols, VP + SC GnRH-a was found to significantly reduce miscarriage events, OR 0.54 (95% CrI 0.37 to 0.80). Subgroup analysis according to ovarian stimulation (OS) protocol revealed that the optimal LPS across both long and short OS, taking into account increase in live birth and reduction in miscarriage as well as OHSS events, was VP + SCGnRH-a, with an OR 2.89 [95% CrI 1.08, 2.96] and OR 2.84 [95% CrI 1.35, 6.26] respectively. Overall, NMA data suggest that combinatorial treatments, with the addition of SCGnRH-a on a VP base result in improved clinical pregnancy and live birth events in both GnRH-agonist and antagonist ovarian stimulation protocols.

## Introduction

Normal luteal function is an essential component for pregnancy maintenance. In natural ovulatory cycles, the corpus luteum can produce adequate progesterone after ovulation until the placental function starts at seven weeks of gestation. Ovarian stimulation (OS) techniques, either with gonadotropin-releasing hormone (GnRH) agonist or antagonist protocols, often induce endocrine defects in the luteal phase with increasing evidence suggesting that the resulting luteal-phase dysfunction may lead to lower pregnancy rates in in vitro fertilization (IVF) and/or ICSI (intracytoplasmic sperm injection) cycles^1,2^. To counteract these effects, luteal-phase support (LPS) is a well-known intervention for almost all stimulated assisted reproductive technology (ART) cycles^3^. Progesterone is amongst the most commonly, exogenously supplemented compounds employed as support of the luteal phase; however, the route of progesterone administration remains controversial^4^. In addition to the route of progesterone supplementation, disparities across literature are also present, regarding LPS dosage, duration and its use as monotherapy or in the context of combinatorial treatment with compounds such as oestradiol, Dehydroepiandrosterone (DHEA), gonadotropin-releasing hormone agonist (GNRH-a) and/or human chorionic gonadotropin (hCG)^2,4^. A plethora of previous pairwise and network meta-analyses has been published in an effort to discern the optimal LPS protocol in fresh cycles^5–10^. However, significant modifiable limitations were recognised. Amongst the pairwise analyses, the one-to-one comparison of specific LPS protocols, dimmed the option of a holistic picture of LPS variability and efficacy to be provided. The homogenisation of LPS protocols under a single agent umbrella did not allow for the appreciation of combinatorial protocols whilst combination of patient populations undergoing both fresh and frozen embryo transfers introduced a significant degree of data bias. Lastly, the effect of LPS selection under different ovarian stimulation protocols had not been previously addressed despite the significant impact upon clinical outcomes^11,12^.

Given the significance of clinical implications of appropriate LPS selection upon pregnancy outcomes, the present network meta-analysis compared mono-and multi-compound LPS regimens for women undergoing fresh cycles of IVF or ICSI in respect to core aspects of IVF/ICSI success (live birth, clinical and biochemical pregnancy rate, miscarriage, multiple pregnancy and ovarian hyperstimulation events). Additionally, the optimal LPS protocol in both agonist and antagonist OS has been explored.

## Methods

**Figure 1:**
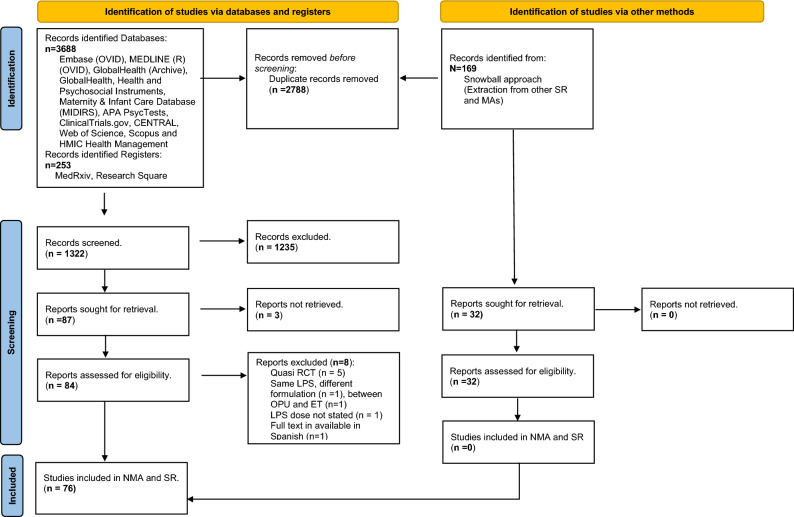
PRISMA Flow chart. PRISMA 2020 flow diagram for new systematic reviews which included searches of databases, registers and other sources.

Search strategy and selection criteria. The present study was prospectively registered under the PROSPERO database CRD42022358986 and conducted according to the PRISMA-NMA checklist^13^. Twelve databases, namely Embase (OVID), MEDLINE (R) (OVID), GlobalHealth (Archive), GlobalHealth, Health and Psychosocial Instruments, Maternity & Infant Care Database (MIDIRS), APA PsycTests, ClinicalTrials.gov, CENTRAL, Web of Science, Scopus and HMIC Health Management Information Consortium and two prospective registers, MedRxiv, Research Square were searched from inception to August 1st 2023. Search strategy was as follows and adapted per requirements of each target database (luteal and (support or supplementation or addition) and (assisted reproduction or IVF or ICSI or in vitro fertilization) and fresh). mp. [mp = ti, ab, hw, tn, ot, dm, mf, dv, kf, fx, dq, cw, ta, te, bt, nm, ox, px, rx, an, ui, sy, ux, mx]. To ensure that all previous meta-synthesised evidence have been identified and assessed, a snowball approach has also been implemented, where the search to the databases described above was also conducted with a limit to include only meta-analyses (N = 102). The original studies included in the relevant meta-analysis manuscripts and were extracted and deduplicated (N = 169). Those were compared to the manuscripts identified through the classical search (Fig. 1). All study designs were included in the initial search but only Randomised Control Trials (RCTs) met abstract selection criteria. No language or geographical restrictions were applied.

For both systematic review and network meta-analysis (NMA), RCTs comparing pharmacological treatments administered for luteal support, either as monotherapy or combinatorial therapy, against placebo or other active agents administered either as mono- or combinatorial therapy for women undergoing fresh IVF/ICSI cycles were included. Studies reporting outcomes from oocyte donation cycles, comparing dosage or timing of same compound, or including patients that had undergone Intrauterine insemination (IUI) or Gamete intrafallopian transfer (GIFT) and zygote intrafallopian transfer (ZIFT) and studies where the route or compound of LPS was not stated or ≥ 4 embryos transferred were excluded (Table S1). Non-blind, single and double-blind studies were included in the analysis. Two independent researchers (SLK, KS) independently selected the studies, reviewed the main reports and supplementary materials, extracted the relevant information from the included trials, and assessed the risk of bias. Any discrepancies were double-checked and resolved by discussion with other members of the review team (GG, NB, DM).

### Data extraction

**Figure 2:**
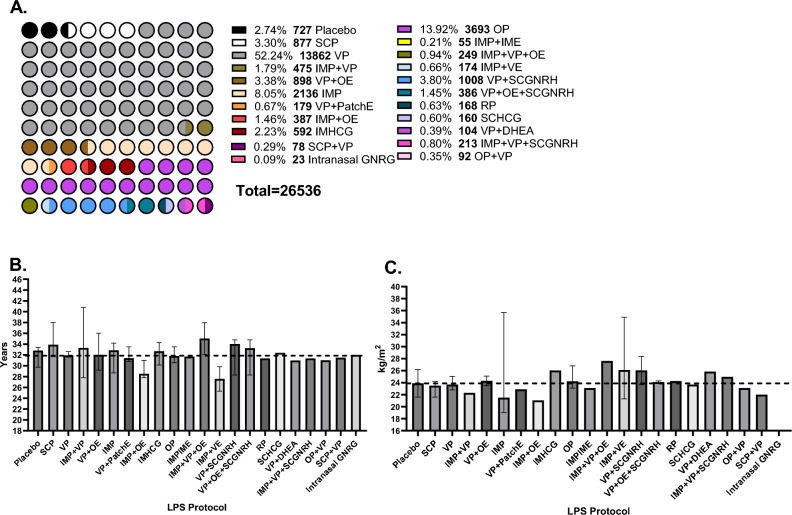
Population percentage and crude numbers exposed to each luteal support regimen and baseline demographic characteristics. Percentage and number of participants exposed to each luteal support protocol (**A**), Comparison of median participant age [95% CrI] (**B**) and median BMI [95% CrI] (**C**) per luteal support intervention. Reference group was considered to be VP. One way ANOVA analysis was employed as data values abided by gaussian distribution. Two decimal p values and asterisk annotation of significance where *p*-value < 0.05, it is flagged with one star (*), *p*-value < 0.01, 2 stars (**), *p*-value < 0.001, three stars (***). placebo (no exposure), SCP (Subcutaneous progesterone), VP (vaginal progesterone), IMP + VP (intramuscular progesterone and vaginal progesterone), VP + OE (vaginal progesterone and oral estradiol), IMP (intramuscular progesterone), VP + PatchE (vaginal progesterone and patch oestrogen), IMP + OE (intramuscular progesterone and oral estradiol), IMHCG (intramuscular hCG), SCP + VP, Intranasal GnRH-a, OP (oral progesterone), IMP + IME (intramuscular progesterone and intramuscular estradiol), IMP + VP + OE (Intramuscular progesterone, vaginal progesterone and oral estradiol), IMP + VE (Intramuscular progesterone and vaginal estradiol), VP + SCGNRH-a [(Vaginal progesterone and subcutaneous GNRH agonist (GNRH-a)], VP + OE + SCGNRH-a (Vaginal progesterone, oral estradiol and subcutaneous GNRH-a), RP (Rectal progesterone), SCHCG (subcutaneous HCG), VP + DHEA (vaginal progesterone and oral DHEA), IMP + VP + SCGNRH-a (Intramuscular progesterone, vaginal progesterone and subcutaneous GNRH-a), OP + VP (oral progesterone and vaginal progesterone).

**Figure 3:**
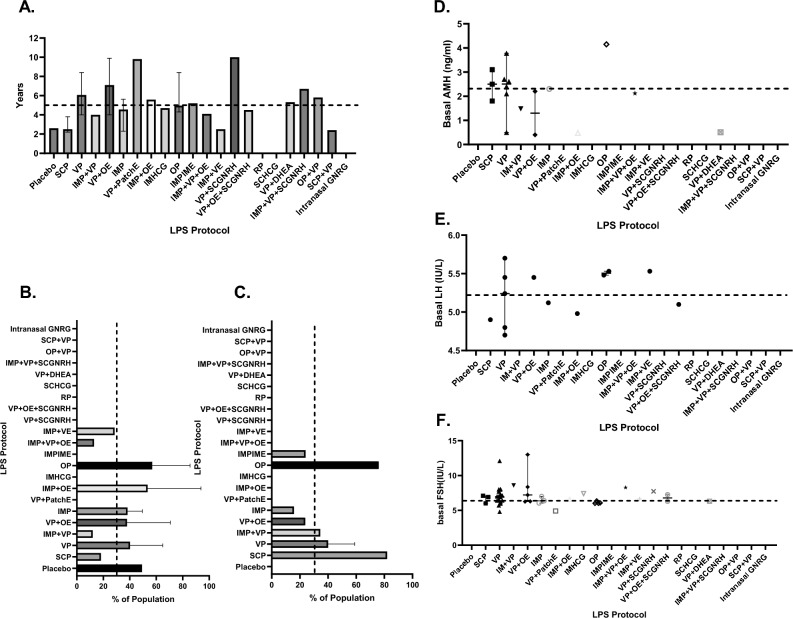
Comparison of clinical parameters [Median, 95% CrI] across treatment groups. Duration of infertility (**A**), Percentage of population diagnosed with primary infertility (**B**) or secondary infertility (**C**), Basal AMH ng/ml (**D**), basal LH IU/L (**E**), FSH IU/L (**F**). Only comparisons that reached statistical significance are depicted. The reference group was PVP. Two decimal p values and asterisk annotation of significance where *p*-value < 0.05, it is flagged with one star (*), *p*-value < 0.01, 2 stars (**), *p*-value < 0.001, three stars (***), *p*-value < 0.0001, four stars (****). LPS (luteal support), AMH (anti-mullerian hormone), FSH (Follicle stimulating hormone), LH (luteinising hormone), placebo (no exposure), SCP (Subcutaneous progesterone), VP (vaginal progesterone), IMP + VP (intramuscular progesterone and vaginal progesterone), VP + OE (vaginal progesterone and oral estradiol), IMP (intramuscular progesterone), VP + PatchE (vaginal progesterone and patch oestrogen), IMP + OE (intramuscular progesterone and oral estradiol), IMHCG (intramuscular hCG), SCP + VP, Intranasal GnRH-a, OP (oral progesterone), IMP + IME (intramuscular progesterone and intramuscular estradiol), IMP + VP + OE (Intramuscular progesterone, vaginal progesterone and oral estradiol), IMP + VE (Intramuscular progesterone and vaginal estradiol), VP + SCGNRH-a [(Vaginal progesterone and subcutaneous GNRH agonist (GNRH-a)], VP + OE + SCGNRH-a (Vaginal progesterone, oral estradiol and subcutaneous GNRH-a), RP (Rectal progesterone), SCHCG (subcutaneous HCG), VP + DHEA (vaginal progesterone and oral DHEA), IMP + VP + SCGNRH-a (Intramuscular progesterone, vaginal progesterone and subcutaneous GNRH-a), OP + VP (oral progesterone and vaginal progesterone).

**Figure 4:**
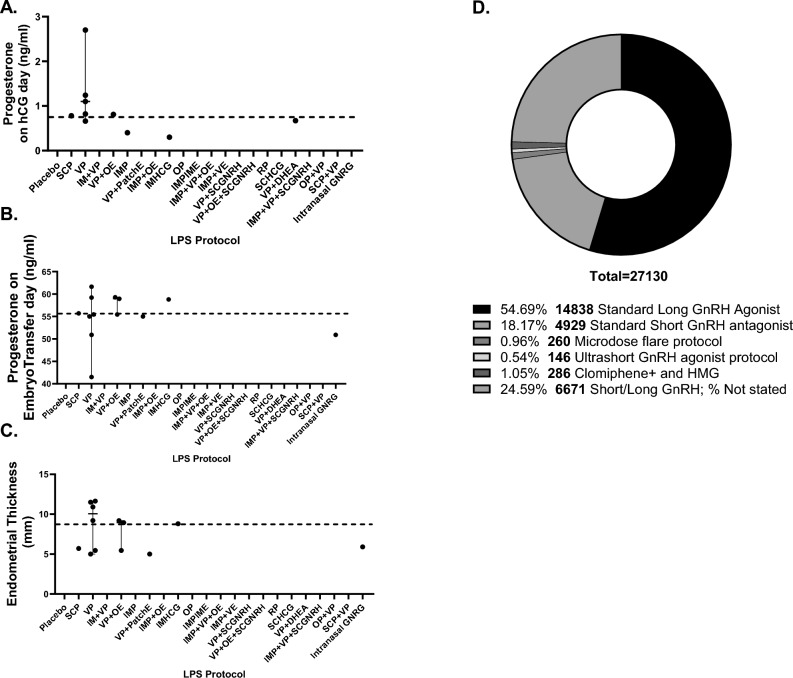
Comparison of clinical parameters [Median, 95% CrI], luteal support regimens duration and dosage across treatment groups. Progesterone levels on hCG trigger day (ng/ml) (**A**), Progesterone levels on embryo transfer (ET) day (ng/ml) (**B**), Endometrial thickness measurement on ET day (**C**), Ovarian stimulation protocol employed (**D**). Only comparisons that reached statistical significance are depicted. The reference group was PVP. Two decimal *p* values and asterisk annotation of significance where *p*-value < 0.05, it is flagged with one star (*), *p*-value < 0.01, 2 stars (**), *p*-value < 0.001, three stars (***), *p*-value < 0.0001, four stars (****). Abbreviations: LPS (luteal support), hCG (Human chorionic gonadotropin), hMG (human menopausal gonadotrophin), placebo (no exposure), SCP (Subcutaneous progesterone), VP (vaginal progesterone), IMP + VP (intramuscular progesterone and vaginal progesterone), VP + OE (vaginal progesterone and oral estradiol), IMP (intramuscular progesterone), VP + PatchE (vaginal progesterone and patch oestrogen), IMP + OE (intramuscular progesterone and oral estradiol), IMHCG (intramuscular hCG), SCP + VP, Intranasal GnRH-a, OP (oral progesterone), IMP + IME (intramuscular progesterone and intramuscular estradiol), IMP + VP + OE (Intramuscular progesterone, vaginal progesterone and oral estradiol), IMP + VE (Intramuscular progesterone and vaginal estradiol), VP + SCGNRH-a [(Vaginal progesterone and subcutaneous GNRH agonist (GNRH-a)], VP + OE + SCGNRH-a (Vaginal progesterone, oral estradiol and subcutaneous GNRH-a), RP (Rectal progesterone), SCHCG (subcutaneous HCG), VP + DHEA (vaginal progesterone and oral DHEA), IMP + VP + SCGNRH-a (Intramuscular progesterone, vaginal progesterone and subcutaneous GNRH-a), OP + VP (oral progesterone and vaginal progesterone).

Events (%, N) of clinical pregnancy, live birth, biochemical pregnancy, miscarriage, multiple pregnancy and OHSS, as previously defined, and the total number of patients exposed per treatment were extracted. Patient demographics and treatment specific parameters were also collected to allow for NMA transitivity analysis and comprehensive exploration of employed treatments across studies. Crude demographic and clinical data were collected. Per study, the total percentage of fresh cycles, Day 3 ETs was calculated from the reported, individual study data (Figs. 2, 3, 4). Missing SD or IQR were calculated from p values, *t* values, and standard error (SE) to allow for data harmonisation. When mean and standard deviation values were recorded, Bland’s method was employed to calculate median and IQR (Wan et al., 2014). Additionally, treatment specific parameters, namely active compound (Progesterone, Estradiol, hCG, GNRH-a, DHEA), brand name, route of administration [O, IM, SC, PV, PR, Topical (Patch)] dose (Progesterone, Estradiol, DHEA and GnRH agonist in mg, hCG in IU, median day of treatment initiation and SD, median end of treatment (weeks) and SD, number of patients exposed to named compound (Table 1). Lastly, implantation and fertilisation rates (%) were extracted as reported per study, given the inclusion of ≥ 1 embryos per study and aggregate data analysed as descriptive statistics (Fig. S1).

**Table 1:** Included studies.

Author et al., year	Country	Time period of study	Sample Size (post-drop outs)	Protocol for IVF (Compound, dose, duration)	Treatments examined	Treatment 1	Treatment 2	Treatment 3	Treatment 4
Gawron et al.^25^	Poland	2015–2016	170	Standard Long GnRH Agonist (35%); Standard Short GnRH antagonist (65%)	OPVP (21) vs. SCPVP (22)	T20: Duphaston, O, 30 mg, OPU till 12 weeks + Crinone 8%, PV, 90 mg, OPU till 12 week, 92	T21: Prolutex, SC, 25 mg, OPU till 12 weeks + Crinone 8%, PV, 90 mg, OPU till 12 week, 78	N/A	N/A
Kao et al.^26^	Taiwan	2019–2022	65	Standard Long GnRH Agonist (18.4%); Standard Short GnRH antagonist (81.6%)	SCP (2) vs. VP (3)	T2: Prolutex, SC, 25 mg, OPU + 2 till 7 weeks, 33	T3: Crinone 8%, PV, 90 mg, OPU + 2 till 7 week, 32	N/A	N/A
Razieh et al.^27^	Iran	Not stated	180	Standard Short GnRH antagonist (100%)	VPSCGNRH (14) vs Placebo (1)	T1: Placebo, 90	T14:Cyclogest, PV, 800 mg, OPU till 11 weeks + Decapeptyl, SC, 0.1 mg once off D3 post ET, 90	N/A	N/A
Iwase et al.^28^	Japan	1993–2003	40	Standard Short GnRH antagonist (100%)	IMP (6) vs OP (10)	T6: Progesterone, IM, 25 mg, from ET till D6 and 50 mg from D7-14, 20	T10: Chormadione acetate 12 mg, O, ET till 2 weeks, 20	N/A	N/A
Moini et al.^29^	Iran	2016 to 2018	80	Standard Long GnRH Agonist	SCP (2) vs VP (3)	T2: Prolutex, SC, 50 mg, OPU till 10 weeks, 40	T3: Cyclogest, PV, 400 mg, OPU till 10 weeks, 40	N/A	N/A
Madkour et al.^30^	Egypt	2011 to 2013	220	Standard Short GnRH antagonist	VP (3) vs VPOE (T5)	T3: Crinone 8%, PV, 180 mg, OPU till 12 weeks, 110	T5: Progyluton, PV, 4 mg, OPU to 7 weeks and Crinone 8%, PV, 180 mg, OPU till 12 weeks, 110	N/A	N/A
Kara et al.^31^	Turkey	2011 to 2013	208	Microdose flare protocol	VP (3) vs VPDHEA (18)	T3: Crinone 8%, PV, 90 mg BD, OPU till 12 weeks, 104	T18: Crinone 8%, PV, 90 mg BD, OPU till 12 weeks and DHEA, O, 75 mg, OPU till 12 weeks,104	N/A	N/A
Serna et al.^32^	Spain	Not stated	160	Standard Long GnRH Agonist	VP (3) vs VPPE (7)	Progeffik, PV, 400 mg, OPU till 10 week, 81	Progeffik, PV, 400 mg, OPU till 10 week and E2 patch Estraderm, 200 microgr, ET to 11 week, 79	N/A	N/A
Fatemi et al.^33^	Belgium	2004 to 2005	201	Standard Long GnRH Agonist	VP (3) vs VPOE (T5)	Utrogestan, PV, 600 mg, OPU + 1 to 7 week, 100	Utrogestan, PV, 600 mg, OPU + 1 to 7 week and Progynova, O, 4 mg, OPU + 1 to 7 week, 101	N/A	N/A
Kleinstein et al.^34^	Germany	1999 to 2001	212	Standard Long GnRH Agonist	VP (3) Tablet vs. gel (gel incl)	Crinone 8%, PV, 270 mg, OPU till 12 week, 212	Utrogestan, PV, 600 mg, OPU till 12 week,, 218	N/A	N/A
Zegers-Hochschild et al.^35^	Brazil	Not stated	505	Standard Long GnRH Agonist	VP (3) vs. IMP (6)	Vaginal ring, PV, 1 g, OPU to 5 weeks, 243	Progesterone, IM, 50 mg, OPU till 5 week, 262	N/A	N/A
Andersen et al.^36^	Denmark	1999 to 2003	153	Standard Long GnRH Agonist	VP (3) early W2 vs W5 withrawal	Progestan, PV, 600 mg, OPU till 5 week, 153	N/A	N/A	N/A
Artini et al.^37^	Italy	Not stated	176	Standard Long GnRH Agonist	Placebo (1) vs. VP (3) vs. IMP (6) vs IMHCG (9)	T1: Placebo, 44	T3: Miconised Progesterone, PV, 100 mg, OPU till 2 weeks, 44	T6: Natural progesterone, IM, 50 mg, OPU to 2 weeks, 44	T9: Profasi, IM, 2000 IU, OPU to 2 weeks, 44
Araujo et al.^38^	USA	Not stated	74	Standard Long GnRH Agonist	IMP (6) vs IMHCG (9)	T6: Progesterone, IM, 50 mg, OPU till 4 weeks, 37	T9: hCG, IM, 2000 IU on 3,6,9,12 post OPU, OPU till 4 weeks, 37	N/A	N/A
Griesinger et al.^39^	Australia, Belgium, China, Germany, Hong Kong, India, Russia, Singapore, Thailand and Ukraine	2015 to 2017	971	Standard Long GnRH Agonist and short GnRH antagonist (%, unknown)	VP (3) vs. OP (10)	T3: Crinone 8%, PV, 90 mg, OPU till 12 week, 481	T10: Duphaston, O, 30 mg, OPU till 12 weeks, 490	N/A	N/A
Goudge et al.^40^	USA	2005 to 2007	46	Standard Long GnRH Agonist and short GnRH antagonist	IMP (6)	T6: Progesterone, IM, 200 mg, OPU till 6 week, 46	N/A	N/A	N/A
Kohls et al.^41^	Spain	2009 to 2010	110	Standard short GnRH antagonist	VP (3)	T3: Miconised Progesterone, PV, 400 mg, OPU till 8 week, 110	N/A	N/A	N/A
Kyrou et al.^42^	Belgium	2008 to 2010	100	Standard short GnRH antagonist	VP (3)	T3: Miconised Progesterone, 600 mg, OPU till 8 week, 100	N/A	N/A	N/A
Prietl et al.^43^	Germany	1989 to Not stated	120	CC (5)/hMG (52)/long GnRH (32) agonist	Placebo (1) vs. IMPIME (11)	T1: Placebo, 65	T11: 17a-hydroxyprogesterone caproate, IM, 1000 mg weekly, OPU till 12 week and oestradiol valerate, 20 mg weekly, OPU till 12 week, 55	N/A	N/A
Ceyhan et al.^44^	Turkey	2006	59	Standard Short GnRH Antagonist	VP (3) vs. VPPE (7)	T3: Progestan, PV, 600 mg, OPU till 8 week, 29	T7: Progestan, PV, 600 mg, OPU till 8 week and Estrogen, Patch, 100 mcg, OPU till 8 week, 30	N/A	N/A
Farhi et al.^45^	Israel	1997 to 1998	271	Standard Long GnRH Agonist (214) and short GnRH antagonist (72)	IMVP (4) vs. IMPVPOE (12)	T4: Geston, IM, 150 mg, OPU + 1 till 6 week and Brand unknown, PV, 100 mg, OPU + 1 till 6 week, 142	T12: Geston, IM, 150 mg, OPU + 1 till 6 week and Brand unknown, PV, 100 mg, OPU + 1 till 6 week and Estrophem, O, 4 mg, OPU + 1 till 6 week, 129	N/A	N/A
Engmann et al.^46^	USA	2004 to 2005	166	Standard Long GnRH Agonist orstandard shor GnRh antagonist or microdose GnRH agonist	IMP (6) vs IMPPVE (13)	T6: Brand not stated, IM, 50 mg, OPU till 6 weeks, 82	T13: Brand not stated, IM, 50 mg, OPU till 6 weeks and micronised E2, PV, 4 mg, ET till week 6, 84	N/A	N/A
Belaisch-Allart et al.^47^	France	1988 to 1989	387	Standard Long GnRH Agonist (67%) or standard short GnRh antagonist (33%)	Placebo (1) vs IMHCG (9)	T1: Placebo, 194	T9: Pregnyl, IM, 1500 IU, OPU and 2 doses, 193	N/A	N/A
Kupferminc et al.^48^	Israel	1988 to 1989	156	Standard Long GnRH Agonist	Placebo (1) vs IMHCG (9) OP (10)	T1: Placebo, 51	T9: hCG, IM, 2500 IU on 3,6,10, ET till 2 weeks, 51	T10: Duphaston, O, 30 mg, ET to 2 weeks, 54	
Aghahosseini et al.^49^	Iran	2008 to 2009	118	Standard Long GnRH Agonist	VP (3) vs. VPOE (5)	T3: Cyclogest, PV, 400 mg, OPU till 12 weeks, 55	T5: Cyclogest, PV, 400 mg, OPU till 12 weeks and Estradiol, O, 4 mg, OPU till 12 weeks, 53	N/A	N/A
Lin et al.^50^	China	2010 to 2011	402	Standard Long GnRH Agonist and short GnRH antagonist	IMP (6) vs. IMPOE (8)	T6: Progesterone oil, IM, 240 mg, OPU till 2 week, 200	T8: Progesterone oil, IM, 240 mg, OPU till 2 weeks and Estradiol valereate, O, 6 mg OD, OPU till 2 weeks, 202	N/A	N/A
Yanushpolsky^51^	USA	Not stated	407	Standard Short GnRH antagonist	VP (3) vs. IMP (6)	T3: Crinone 8%, 90 mg, PV, dose, OPU to 10 week, 206	T6: Progesterone, IM, 50 mg, OPU to 10 week, 201	N/A	N/A
Elgindy et al.^52^	Egypt	2004 to 2006	270	Standard Short GnRH antagonist	IMP (6) vs. IMPOE (8) vs. IMPPVE (13)	T6: Gestone, IM, 100 mg, ET to 6 week, 90	T8: Gestone, IM, 100 mg, ET to 6 week and Cycloprogynova, O, 6 mg, ET to 6 week, 90	T13:Gestone, IM, 100 mg, ET to 6 week and Cycloprogynova, PV, 6 mg, ET to 6 week, 90	N/A
Isik et al.^53^	Turkey	2005	159	Standard Short GnRH antagonist	VP (3) vs VPSCGNRH (14)	T3: Progestan, PV, 600 mg, OPU till 2 weeks, 80	T14: Progestan, PV, 600 mg, OPU till 2 weeks and Leuprolide acetate, SC, 0.5 mg once off D6 post ET, 74	N/A	N/A
Yildiz et al.^54^	Turkey	2008 to 2010	279	Standard Long GnRH Agonist	VPOE (5) vs. VPOESCGNRH (15)	T5: Progestan, O, 600 mg, OPU to 2 weeks and Estrofem, O, 4 mg, OPU to 2 weeks, 95	T15: Progestan, PV, 600 mg OD, OPU to 2 weeks and Estrofem, O, 4 mg OD, OPU to 2 weeks and leuprolide acetate, SC, 1/2 mg mg once off 3D post ET, 100	N/A	N/A
Dal Prato et al.^55^	Italy	2001 to 2004	412	Standard Long GnRH Agonist	VP (3) vs IMP (6)	T3: Crinone 8%, PV, 90 mg/180 mg, OPU + 1 till 5 week, 274	T6: Prontogest, IM, 50 mg, OPU + 1 till 5 week, 138	N/A	N/A
Propst et al.^56^	USA	1998 to 1999	201	Standard Long GnRH Agonist	VP (3) vs IMP (6)	T3: : Crinone 8%, PV, 90 mg, OPU till 10 weeks, 102	T6: Progesterone, IM, 50 mg, OPU till 10 weeks, 99	N/A	N/A
Chakravarty et al.^57^	India	2002 to 2003	430	Standard Long GnRH Agonist	VP (3) vs oP (10)	T3: Utrogestan, PV, 200 mg, ET + 1 till 2 weeks, 32	T10: Dydrogesterone, O, 20 mg, ET till 12 weeks, 79	N/A	N/A
Friedler et al.^58^	Israel	Not stated	64	Standard Long GnRH Agonist	VP (3) vs OP (10)	T3: Utrogestan, PV, 200 mg, ET + 1 till 2 weeks, 32	T10: Utrogestan, O, 800 mg, ET + 1 till 2 weeks, 32	N/A	N/A
Pouly et al.^59^	Belgium	Not stated	283	Standard Long GnRH Agonist	VP (3) vs OP (10)	T3: Crinone 8%, PV, 90 mg, ET till 13 weeks,139	T10: Utrogestan, O, 300 mg, ET till 13 weeks, 144	N/A	N/A
Salehpour et al.^60^	Iran	2014 to 2015	210	Standard Long GnRH Agonist (107) and short GnRH antagonist (103)	VP (3) vs OP (10)	T3: Cyclogest, PV, 800 mg, OPU till 12 weeks, 114	T10: Duphaston, O, 40 mg, OPU till 12 weeks, 96	N/A	N/A
Bergh et al.^61^	Denmark	2006 to 2010	1983	Standard Long GnRH Agonist	VP (3)	T3: Gel/Tablets, PV, 200 mg/600 mg/ 90 mg (gel)OPU to 5 weeks, 1983	N/A	N/A	N/A
Doody et al.^62^	USA	2005 to 2008	1211	Standard Long GnRH Agonist	VP (3)	T3: Gel/Tablets, PV, 200 mg/600 mg/ 90 mg (gel), OPU to 10 weeks, 1211	N/A	N/A	N/A
Tay et al.^63^	UK	1999 to 2000	161	Standard Long GnRH Agonist	VP (3) vs PRP (16) SCHCG (17)	T3: Crinone/Utrogestan, PV, 90 mg (gel), 200 mg/400 mg/600 mg (tablets), OPU to 2 weeks, 91	T16: Cyclogest, PR, 400 mg, OPU till 2 weeks, 35	T17: hCG, SC, 1500 IU, OPU + 2 and OPU + 7, twice off to 2 weeks , 35	N/A
Abate et al.^64^	Italy	1997 to 1998	156	Standard Long GnRH Agonist	PLACEBO (1) vs. VP (3) vs. IMP (6)	T1: placebo, 52	T3: Progesterone gel, PV, 90 mg OD, ET + 1 till 2 weeks, 52	T6: Progesterone, IM, 50 mg, ET + 1 till 2 weeks, 52	N/A
Abate et al.^65^	Italy	1996 to 1997	86	Standard Long GnRH Agonist	PLACEBO (1) vs IMP (6)	T1: placebo, 43	T6: 17-OHPc, IM, 50 mg, OPU + 1 till 2 weeks, 43	N/A	N/A
Aboulghar et al.^66^	Egypt	2011 to 2012	446	Standard Long GnRH Agonist	VP (3) vs. VPSCGNRH (14)	T3: Prontogest, PV, 600 mg, OPU till 2 weeks	T14: Decapeptyl, SC; 0.1 mg OD, OPU till 2 weeks; Prontogest, PV, 600 mg, OPU till 2 weeks	N/A	N/A
Aghsa et al.^67^	Iran	Not stated	147	Standard Short GnRH antagonist	VP (3) vs. PRP (16)	T3:Cyclogest, PV,800 mg, OPU till 6 weeks	T16: Cyclogest, PR,800 mg, OPU till 6 weeks	N/A	N/A
Ata et al.^68^	Turkey	2006 to 2007	570	Standard Long GnRH agonist protocol	VP (3) vs. VPSCGNRH (14)	T3: Crinone 8%, PV, 90 mg, OPU till 10 weeks, 285	T14: Decapeptyl, SC, 0.1 mg, six days after ICSI and Crinone 8%, PV, 90 mg, OPU till 10 weeks, 285	N/A	N/A
Baker et al.^69^	USA	2009–2011	800	GnRH agonist (long and flare protocols) and GnRH antagonist	SCP (2) vs. VP (3)	T2: Prolutex, SC, 25 mg, OPU till 12 weeks, 400	T3: Endometrin, PV, 200 mg, OPU till 12 weeks, 400	N/A	N/A
Ganesh et al.^70^	India	Not stated	904	Standard Long GnRH Agonist	VP (3) vs OP(10)	T3: Crinone 8%, PV, 90 mg, OPU till 12 weeks, 482	T10: Duphaston, O, 20 mg, OPU till 12 weeks, 422	N/A	N/A
Golan et al.^71^	Israel	Not stated	56	Ultrashort GnRH agonist protocol	IMP (T6) vs. IMHCG (T9)	T6: Progesterone, IM, 100 mg, from ET till 2 weeks, 26	T9: HCG, IM, 1000 IU or 2500 IU, from ET every 3 days for 2 weeks, 30	N/A	N/A
Inamdar et al.^72^	India	2010 to 2011	426	long GnRH agonist protocol	IMVP (T4) vs. IMVPSCGNRH (T19)	T4: Micronized progesterone, PV, 800 mg followed by progesterone, IM, 100 mg, OPU till 10 weeks, 213	T19: Micronized progesterone, PV, 800 mg followed by progesterone, IM, 100 mg, OPU till 10 weeks + Lupride 1 mg 6th, 7th and 8th days after OPU	N/A	N/A
Lockwood et al.^73^	Europe	2009 to 2010	683	GnRH agonist and GnRH antagonist	SCP (2) vs. VP (3)	T2:Prolutex, SC, 25 mg, OPU till 8 weeks, 339	T3: Crinone 8%, PV, 90 mg, OPU till 8 weeks, 344	N/A	N/A
Martinez et al.^74^	Spain	1996	310	Standard Long GnRH Agonist	VP (3) vs. IMHCG (T9)	T3:Utrogestan, PV, 300 mg, ET till 10 days,168	T9:HCG, IM, 2500 IU on 2,4,6, ET till 6 days, 142	N/A	N/A
Patki et al.^75^	India	2004 to 2005	675	Standard Long GnRH Agonist	VP (3) vs. OP (T10)	T3: Utrogestan, PV, 600 mg, OPU till 10 weeks, 309	T10: Dydrogesterone, O, 30 mg, OPU till 10 weeks, 150	N/A	N/A
Stadtmauer et al.^76^	USA	2008 to 2009	1297	long GnRH agonist protocol	VP (3)	T3: Ring/ Gel, PV, 11 mg (ring), 90 mg(gel), OPU + 1 till 10 weeks, 1297	N/A	N/A	N/A
Tesarik et al.^77^	Spain	2003 to 2005	572	Long GnRH agonist and GnRH antagonist protocol	VPOE (T5) vs. VPOESCGNRH (T15)	T5:Utrogestan, PV, 400 mg OPU till 17 days and Progynova, O, 4 mg, OPU till 17 days, 286	T15:Utrogestan, PV, 400 mg, OPU till 17 days and Progynova, O, 4 mg, OPU till 17 days and triptorelin, 0.1 mg, 6 days after ICSI, 286	N/A	N/A
Tournaye et al.^78^	Multicountry	2013 to 2016	974	Not stated	VP (3) VS OP (T10)	T3:Utrogestan, PV, 600 mg, OPU till 12 weeks, 477	T10:Dydrogesteron, O, 30 mg, OPU till 12 weeks, 497	N/A	N/A
Michnova et al.^79^	Czech Republic	Not stated	58	Long GnRH agonist and GnRH antagonist protocol	VP (3)	T3:Utrogestan, PV, 600 mg, OPU till 12 weeks, 477	N/A	N/A	N/A
Elgindy et al.^80^	Egypt	2015 to 2016	190	Standard Short GnRH antagonist	VPOE (5) vs. IMPOE (8)	T3:Endometrin, PV, 300 mg, OPU to 8 week and Estradiol valearate, O, 6 mg, OPU to 8 week, 95	T8: Prontogest IM, 100 mg, OPU till 8 weeks and Estradiol valereate, O, 6 mg, OPU till 8 weeks, 95	N/A	N/A
Yang et al.^81^	China	2015 to 2017	983	Long GnRH agonist and GnRH antagonist protocol	VP (3) VS. OP(10)	T3:Crinone 8%, PV, 90 mg/180 mg, OPU till 12 week, 489	T10: Duphaston, O, 30 mg, OPU till 12 week, 494	N/A	N/A
Tomic et al.^82^	Croatia	2010 to 2013	853	Standard Long GnRH Agonist	VP (3) VS. OP(10)	T3: Crinone 8%, PV, 90 mg/180 mg, OPU till 10 week, 416	T10: Duphaston, O, 20 mg, OPU till 10 week, 415	N/A	N/A
Gizzo et al.^83^	Italy	2010 to 2013	360	Standard Long GnRH Agonist (50%) ; Standard Short GnRH antagonist (25%); Short agonist (25%)	VP (3) VS. IMVP (4) VS. IMPVPOE (12)	T3:Progesterone, PV, 400 mg, OPU + 1 till ?12 weeks, 120	T4: Progesterone IM, 100 mg, OPU + 1 till 12 week and Brand unknown, PV, 600 mg, OPU + 1 till 12 week, 120	T12:Progesterone IM, 100 mg, OPU + 1 till 12 week and Brand unknown, PV, 600 mg, OPU + 1 till 12 week + valerate E2, O, 4 mg, OPU + 1 till 12 week; 120	N/A
Kutlusoy et al.^84^	Turkey	2008 to 2009	60	Standard Long GnRH Agonist or Standard Short GnRH antagonist	VP (3) VS. VPOE (5)	T3:Crinone 8%, PV, 90 mg, OPU till 10 week, 33	T5:Crinone 8%, PV, 90 mg, OPU till 10 week, and Estrofem, O, 2 mg, OPU to 10 weeks, 27	N/A	N/A
Ozer et al.^85^	Turkey	2019	134	Not stated	VP (3) VS. OP(10)	T3:Crinone 8%, PV, 90 mg, OPU till 12 week, 67	T10:Duphaston, O, 30 mg, OPU till 12 weeks, 67	N/A	N/A
Saharkhiz et al.^86^	Iran	2014 to 2015	210	Standard Long GnRH Agonist; Standard Short GnRH antagonist	VP (3) VS. OP(10)	T3:Cyclogest, PV, 800 mg, OPU till 12 weeks, 114	T10:Duphaston, O, 40 mg, OPU till 12 weeks, 96	N/A	N/A
Horowitz et al.^87^	Israel	2012 to 2018	59	Standard Long GnRH Agonist (34%); Standard Short GnRH antagonist (66%)	PLACEBO (T1) vs. VP (3)	T1: placebo, 43	T3:Endometrin, PV, 200 mg, OPU till 12 week , 31	N/A	N/A
Belaisch-Allart et al.^88^	France	1985 to 1986	286	clomiphene + and HMG , pure FSH, Programmed cycles	VP (3) VS. OP(10)	T1: placebo, 145	T10:Duphaston, O, Not stated, OPU till 3 weeks, 141	N/A	N/A
Chi et al.^89^	China	Not stated	1058	Standard Short GnRH antagonist	VP (3) VS. IMP (T6)	T3:Crinone 8%, PV, 90 mg, OPU till 6 week, 527	T6: Progesterone, IM, 60 mg, from ET till 6 weeks, 531	N/A	N/A
Fusi et al.^90^	Italy	2013 to 2015	1344	Standard Short GnRH antagonist	VP (3) VS. VPSCGNRH (T14)	T3:Cyclogest, PV, 600 mg, OPU till 12 weeks, 241	T14: Triptorelin, SC, 0.1 mg, from day of ET and every other day for 5 doses and Cyclogest, PV, 600 mg, OPU till NS weeks,507	N/A	N/A
Gorkemli et al.^91^	Turkey	2001 to 2003	144	Standard Long GnRH Agonist (100%)	VP (3) vs. VPPE (7)	T3:Progestan , PV, 600 mg, OPU till 10 weeks, 74	T7: Progestan, PV, 600 mg, OPU till 8 week and Estraderm T, Patch, 100 mcg, OPU till 10 week, 70	N/A	N/A
Ibrahem et al.^92^	Egypt	2016 to 2019	564	Standard Long GnRH Agonist (100%)	VP (3) vs OP (10)	T3:Prontogest , PV, 800 mg, OPU till 14 weeks, 280	T10:Duphaston, O, 30 mg, OPU till 12 weeks, 284	N/A	N/A
Kapur et al.^93^	India	Not stated	150	Standard Long GnRH Agonist (100%)	VP (3) vs VPOE (5)	T3:Micronized progesterone, PV, 800 mg, ET till 14 weeks, 75	T5:Micronized progesterone, PV, 800 mg, ET till 14 weeks + Estradiol valerate, O, 4 mg, ET till 14 weeks, 75	N/A	N/A
Khrouf et al.^94^	Tunisia	Not stated	126	Standard Long GnRH Agonist (73%); Standard Short GnRH antagonist (27%)	VP (3) vs PRP (16)	T3:Cyclogest , PV, 600 mg, ET till 14 weeks, 68	T16:Cyclogest , PR, 600 mg, ET till 14 weeks, 58	N/A	N/A
Kwon et al.^95^	Korea	Not stated	110	tandard Short GnRH antagonist	VP (3) vs VPOE (5)	T3:Crinone 8%, PV, 90 mg, OPU till 10 week, 55	T5:Micronized progesterone, PV, 800 mg, OPU till 10 weeks + Estradiol valerate, O, 4 mg, OPU till 10 weeks, 55	N/A	N/A
Mele et al.^96^	Italy	2017	130	Standard Long GnRH Agonist	SCP (T2) vs IMP (T6)	T2:Prolutex, SC, 25 mg, OPU till NS weeks, 65	T6: Progesterone, IM,33 mg from OPU and 50 mg, from ET till NS weeks, 65	N/A	N/A
Zargar et al.^97^	Iran	2014 to 2015	612	Standard Long GnRH Agonist (NS%); Standard Short GnRH antagonist (NS%)	VP (T3) vs IMP (T6) vs. OP (10)	T3:Prontogest , PV, 800 mg, ET till 12 weeks, 200	T6: Progesterone, IM, 100 mg, from ET till 12 weeks, 200	T10:Duphaston, O, 30 mg, ET till 12 weeks, 212	N/A
Pirard et al.^98^	Belgium	Not stated	53	Standard Short GnRH antagonist	VP (T3) vs. INGNRH ( T22)	T3:NS,600 mg, PV, OPU till 12 weeks, 18	T22:Buserelin, 200mcg, , followed by 100 μg IN buserelin TDS for luteal support starting the next day of ovulation trigger, 35	N/A	N/A
Var et al.^99^	Turkey	2007 to 2008	288	Standard Long GnRH Agonist	VP (T3) vs. VPOE (T5) vs. IMHCG (T9)	T3:Crinone 8%, PV, 12 mg, ET till 10 week, 97	T5:Crinone 8%, PV, 12 mg, ET till 10 week + Estrofem, O, 4 mg, OPU till 10 weeks, 96	T9: hCG, ,500 IU of hCG IM on the ET day, as well as 3 and 6 days, 95	N/A
Humaidan^100^	Denmark	2014 to 2019	250	Standard Short GnRH antagonist	VP (T3) vs. SCHCG (T17)	T3:Lutinus, Dose not stated, TDS, OPU till 2 weeks, 125	T17: Two groups 1500 IU at OPU and second dose 1000 IU at OPU + 4; Second group 1000 IU HCG at OPU and 500 IU at OPU + 4,125	N/A	N/A

Patient sample, ovarian stimulation protocol, luteal support comparison and regimen.

Placebo, no exposure; SCP, Subcutaneous progesterone; VP, vaginal progesterone; IMP + VP, intramuscular progesterone and vaginal progesterone; VP + OE, vaginal progesterone and oral estradiol; IMP, intramuscular progesterone; VP + PatchE, vaginal progesterone and patch oestrogen; IMP + OE, intramuscular progesterone and oral estradiol; IMHCG, intramuscular hCG; SCP + VP, Intranasal GnRH-a; OP, oral progesterone; IMP + IME, intramuscular progesterone and intramuscular estradiol; IMP + VP + OE, Intramuscular progesterone, vaginal progesterone and oral estradiol; IMP + VE, Intramuscular progesterone and vaginal estradiol; VP + SCGNRH-a, Vaginal progesterone and subcutaneous GNRH agonist (GNRH-a); VP + OE + SCGNRH-a, Vaginal progesterone, oral estradiol and subcutaneous GNRH-a; RP, Rectal progesterone; SCHCG, subcutaneous HCG; VP + DHEA, vaginal progesterone and oral DHEA; IMP + VP + SCGNRH-a, Intramuscular progesterone, vaginal progesterone and subcutaneous GNRH-a; OP + VP, oral progesterone and vaginal progesterone.

### Outcomes

The NMA primary outcomes were clinical pregnancy, defined as the presence of a gestational sac, with or without a fetal heartbeat on ultrasonography (US) and live birth, defined as the number of deliveries that resulted in live born neonate/s. Regarding live birth, singleton and non-singleton deliveries were considered as a single event. Secondary outcomes included biochemical pregnancy, defined as positive hCG test but without US verification 2 weeks following embryo transfer (ET), miscarriage defined as the spontaneous loss of a pregnancy before the 20th week, multiple pregnancy was defined as non-singleton clinical pregnancy and OHSS events. Crude events were collected per included study, and therefore no homogenisation of extracted data was required.

### Data analysis

**Figure 5:**
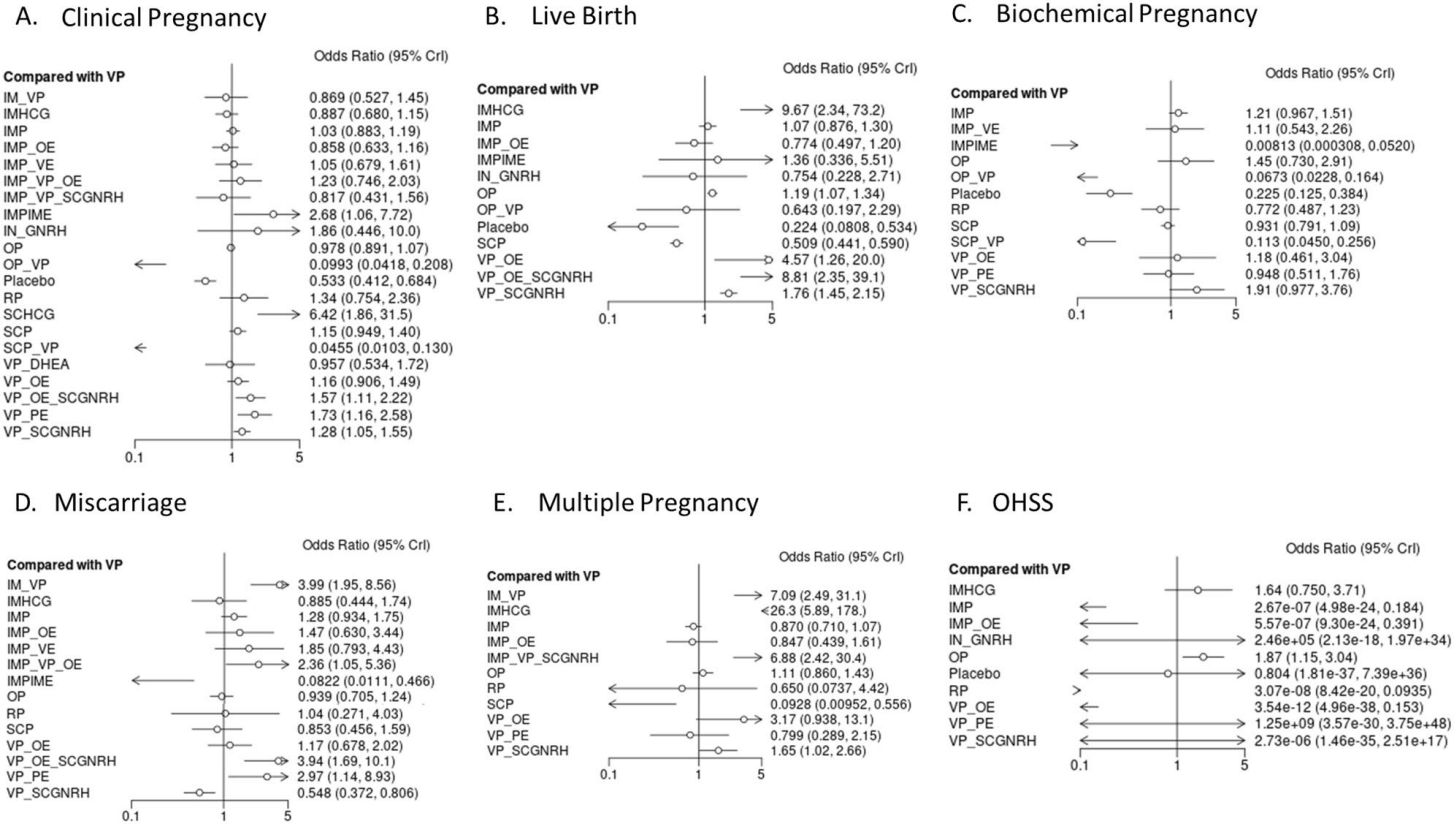
Luteal support Bayesian fixed effect consistency forest plot (Odds ratio, 95% CrI) for Clinical Pregnancy (**A**) Live Birth (**B**) Biochemical Pregnancy (**C**) Miscarriage (**D**) and Multiple pregnancy (**E**) OHSS (**F**) outcomes). Graph generated by MetaInsight R package. Tabular results of design-by-treatment interaction model consistency depicted in Table S1–S2 per outcome. Node splitting model per comparison (direct and indirect effects) depicted in Table S.6–7. placebo (no exposure), SCP (Subcutaneous progesterone), VP (vaginal progesterone), IMP + VP (intramuscular progesterone and vaginal progesterone), VP + OE (vaginal progesterone and oral estradiol), IMP (intramuscular progesterone), VP + PatchE (vaginal progesterone and patch oestrogen), IMP + OE (intramuscular progesterone and oral estradiol), IMHCG (intramuscular hCG), SCP + VP, Intranasal GnRH-a, OP (oral progesterone), IMP + IME (intramuscular progesterone and intramuscular estradiol), IMP + VP + OE (Intramuscular progesterone, vaginal progesterone and oral estradiol), IMP + VE (Intramuscular progesterone and vaginal estradiol), VP + SCGNRH-a [(Vaginal progesterone and subcutaneous GNRH agonist (GNRH-a)], VP + OE + SCGNRH-a (Vaginal progesterone, oral estradiol and subcutaneous GNRH-a), RP (Rectal progesterone), SCHCG (subcutaneous HCG), VP + DHEA (vaginal progesterone and oral DHEA), IMP + VP + SCGNRH-a (Intramuscular progesterone, vaginal progesterone and subcutaneous GNRH-a), OP + VP (oral progesterone and vaginal progesterone).

Effect estimates were calculated as odds ratios (ORs) for all outcomes, given that all were dichotomous, with respective 95% credibility intervals (95% CrIs) using Bayesian network and pair-wise meta-analysis^14^ (Fig. 5, Fig. S2–S10). Of note, a credibility interval is an interval within which an unobserved parameter value falls with a particular probability in Bayesian statistics comparable to the 95% Confidence interval commonly seen in frequentist statistics^15^. Network meta-analysis iterations were conducted with MetaInsight visual R package^16^. NMA was conducted using a fixed-effects model within a Bayesian setting, as unequal heterogeneity across all comparisons was assumed. Vaginal Progesterone (VP) was used as the reference treatment given its proven superiority over placebo and the NICE guideline recommendations^17^. A hierarchy of treatments was calculated for each outcome, based on the p-scores and SUCRA ratings. Summary of the rank distribution of LPS treatments, interpreted as the estimated proportion of treatments worse than the treatment of reference (VP) was displayed by Litmus Rank-O-Gram graphs and Radial SUCRA^18^ (Fig. S5–S6). Transitivity assumption was evaluated by comparing the distribution of key study characteristics across studies grouped by comparison (age and BMI). We assessed inconsistency between direct and indirect sources of evidence using global and local approaches. We assessed global inconsistency by using a design-by-treatment test^19,20^. Local inconsistency was evaluated by using the back calculation and separate indirect from direct design evidence methods, comparing direct and indirect evidence for each pairwise treatment comparison and node-splitting model^21^ (Table S2–S3; Fig. S3–S4). Possible heterogeneity of treatment effects and the robustness of findings was explored by subgroup network meta-analyses including only trials at overall low and medium risk of bias (Table 1, Fig. S7–S8, S10). Further subgroup analysis was conducted on trials using either standard (long) GnRH agonist or standard (short) GnRH antagonist protocol for ovarian stimulation to limit data heterogeneity. If mixed populations were included in the original publication, a cut-off of ≥ 65% of patients being treated with either of the protocols, was employed to categorise studies according to subgroup (Table S4). Mixmeta package in R v4.1.2 was employed for confounder exploration in a network meta-regression model. Gelman network convergence, network deviance and ranking analysis were conducted to quantify overall network discordance (Fig. S9–S10). Intergroup differences regarding demographic and treatment parameters were quantified, where appropriate by ANOVA (for parametric distributed variables e.g., Age, BMI) or Kruskal–Wallis test (non-parametric distribution of variables, e.g., all remaining variables). Multilevel network meta‐regression for the embryological parameters (number of transferred embryos, number of retrieved and mature oocytes, peak estradiol, % of day 3 embryos transferred) was undertaken for both primary and secondary outcomes^22^ (Table S11–S12).

### Risk of bias assessment

Within-study bias was assessed with the Cochrane risk of bias tool RoB2^23^ and the certainty of evidence using the GRADE Framework (Table 2). Overall network risk of bias was assessed with the Network Meta-Analysis framework (CINeMA)^24^ (Table S5–S10). Small-study effects and publication bias for each treatment pair was assessed using a contour-enhanced funnel plot.

**Table 2:** RoB Tool V. 2 risk of bias assessment and GRADE certainty in evidence ratings per study.

References	RoB Tool 2								GRADE					
	Random sequence generation	Allocation concealment	Performance bias	Detection bias	Attrition bias	Reporting bias	Other	Collectively	Risk of Bias	Imprecision	Inconsistency	Indirectness	Publication bias	Collectively
Gawron et al.^25^	Low	Moderate	Low	Low	Low	Low	Low	1	⨁⨁⨁⨁	⨁⨁⨁⨁	⨁⨁⨁⨁	⨁⨁⨁⨁	⨁⨁⨁⨁	⨁⨁⨁⨁
Kao et al.^26^	Low	Moderate	Moderate	Moderate	Low	Moderate	Moderate	2	⨁⨁⨁◯	⨁⨁⨁◯	⨁⨁⨁◯	⨁⨁⨁◯	⨁⨁⨁⨁	⨁⨁⨁◯
Razieh et al.^27^	Low	Low	Low	Low	Low	Low	Low	1	⨁⨁⨁⨁	⨁⨁⨁⨁	⨁⨁⨁⨁	⨁⨁⨁⨁	⨁⨁⨁⨁	⨁⨁⨁⨁
Iwase et al.^28^	Low	High	Low	Low	Low	Low	Low	1	⨁⨁⨁⨁	⨁⨁⨁⨁	⨁⨁⨁⨁	⨁⨁⨁⨁	⨁⨁⨁⨁	⨁⨁⨁⨁
Moini et al.^29^	Unclear	Low	Low	Low	Low	Low	Low	1	⨁⨁⨁⨁	⨁⨁⨁⨁	⨁⨁⨁⨁	⨁⨁⨁⨁	⨁⨁⨁⨁	⨁⨁⨁⨁
Madkour et al.^30^	Low	Low	Low	Low	Low	Low	Low	1	⨁⨁⨁⨁	⨁⨁⨁⨁	⨁⨁⨁⨁	⨁⨁⨁⨁	⨁⨁⨁⨁	⨁⨁⨁⨁
Kara et al.^31^	Low	Unclear	Low	Low	Low	Unclear	Unclear	2	⨁⨁⨁⨁	⨁⨁⨁⨁	⨁⨁⨁⨁	⨁⨁⨁◯	⨁⨁⨁⨁	⨁⨁⨁⨁
Serna et al.^32^	Low	Low	High	High	Low	Low	Low	3	⨁⨁⨁⨁	⨁⨁⨁⨁	⨁⨁⨁⨁	⨁⨁⨁◯	⨁⨁⨁⨁	⨁⨁⨁⨁
Fatemi et al.^33^	Low	High	High	High	Low	Low	High	3	⨁⨁◯◯	⨁⨁⨁◯	⨁⨁⨁◯	⨁⨁⨁⨁	⨁⨁⨁⨁	⨁⨁◯◯
Kleinstein et al.^34^	Low	Low	High	High	Low	Low	Unclear	3	⨁⨁◯◯	⨁⨁⨁◯	⨁⨁⨁◯	⨁⨁⨁⨁	⨁⨁⨁⨁	⨁⨁◯◯
Zegers-Hochschild et al.^35^	High	Unclear	Unclear	Unclear	Unclear	Low	Low	3	⨁⨁⨁◯	⨁⨁⨁◯	⨁⨁⨁◯	⨁⨁⨁◯	⨁⨁⨁⨁	⨁⨁⨁◯
Andersen et al.^36^	Low	High	Unclear	Unclear	Low	Low	Unclear	3	⨁⨁⨁◯	⨁⨁⨁◯	⨁⨁⨁◯	⨁⨁⨁◯	⨁⨁⨁⨁	⨁⨁⨁◯
Artini et al.^37^	High	Unclear	Unclear	Unclear	Unclear	Unclear	Low	3	⨁⨁◯◯	⨁⨁⨁◯	⨁⨁⨁◯	⨁⨁⨁◯	⨁⨁⨁⨁	⨁⨁◯◯
Araujo et al.^38^	High	Unclear	Unclear	Unclear	Unclear	Unclear	Unclear	2	⨁⨁◯◯	⨁⨁⨁◯	⨁⨁⨁◯	⨁⨁⨁◯	⨁⨁⨁⨁	⨁⨁◯◯
Griesinger et al.^39^	Low	Low	Low	Low	Low	Low	Low	1	⨁⨁⨁⨁	⨁⨁⨁⨁	⨁⨁⨁⨁	⨁⨁⨁⨁	⨁⨁⨁⨁	⨁⨁⨁⨁
Goudge et al.^40^	Low	Low	Unclear	Unclear	Low	Unclear	Low	2	⨁⨁⨁◯	⨁⨁⨁⨁	⨁⨁⨁◯	⨁⨁⨁⨁	⨁⨁⨁⨁	⨁⨁⨁◯
Kohls et al.^41^	Low	Low	High	High	Low	Low	Low	2	⨁⨁◯◯	⨁⨁⨁◯	⨁⨁⨁◯	⨁⨁⨁◯	⨁⨁⨁⨁	⨁⨁◯◯
Kyrou et al.^42^	Low	Low	High	High	Low	Low	Unclear	3	⨁⨁◯◯	⨁⨁⨁◯	⨁⨁⨁◯	⨁⨁⨁◯	⨁⨁⨁⨁	⨁⨁◯◯
Prietl et al.^43^	High	High	Unclear	Unclear	Low	Low	Low	3	⨁⨁◯◯	⨁⨁◯◯	⨁⨁◯◯	⨁⨁⨁◯	⨁⨁⨁⨁	⨁◯◯◯
Ceyhan et al.^44^	Low	Low	High	High	Low	Low	Low	3	⨁⨁◯◯	⨁⨁⨁◯	⨁⨁⨁⨁	⨁⨁⨁⨁	⨁⨁⨁⨁	⨁⨁⨁◯
Farhi et al.^45^	Low	Low	Unclear	Unclear	Low	Low	Low	2	⨁⨁⨁◯	⨁⨁⨁⨁	⨁⨁⨁⨁	⨁⨁⨁⨁	⨁⨁⨁⨁	⨁⨁⨁⨁
Engmann et al.^46^	Low	Low	High	High	Low	Low	Low	3	⨁⨁◯◯	⨁⨁⨁◯	⨁⨁⨁⨁	⨁⨁⨁⨁	⨁⨁⨁⨁	⨁⨁⨁◯
Belaisch-Allart et al.^47^	Low	Unclear	Unclear	Unclear	Unclear	Unclear	Low	2	⨁⨁⨁◯	⨁⨁⨁⨁	⨁⨁⨁⨁	⨁⨁⨁⨁	⨁⨁⨁⨁	⨁⨁⨁⨁
Kupferminc et al.^48^	Unclear	Unclear	Unclear	Unclear	Unclear	Unclear	Low	2	⨁⨁⨁◯	⨁⨁⨁◯	⨁⨁⨁⨁	⨁⨁⨁⨁	⨁⨁⨁⨁	⨁⨁⨁◯
Aghahosseini et al.^49^	Low	High	High	High	Low	High	Low	3	⨁⨁◯◯	⨁⨁⨁◯	⨁⨁⨁⨁	⨁⨁⨁⨁	⨁⨁⨁⨁	⨁⨁⨁◯
Lin et al.^50^	Low	Unclear	High	High	Low	Low	Low	3	⨁⨁◯◯	⨁⨁⨁◯	⨁⨁⨁⨁	⨁⨁⨁⨁	⨁⨁⨁⨁	⨁⨁⨁◯
Yanushpolsky^51^	Low	Low	High	High	Low	Low	Low	3	⨁⨁◯◯	⨁⨁⨁◯	⨁⨁⨁⨁	⨁⨁⨁⨁	⨁⨁⨁⨁	⨁⨁⨁◯
Elgindy et al.^52^	Low	Low	Unclear	Unclear	Low	Low	Low	2	⨁⨁⨁◯	⨁⨁⨁⨁	⨁⨁⨁⨁	⨁⨁⨁⨁	⨁⨁⨁⨁	⨁⨁⨁⨁
Isik et al.^53^	Low	Low	Unclear	Low	Low	Low	Low	1	⨁⨁⨁⨁	⨁⨁⨁⨁	⨁⨁⨁⨁	⨁⨁⨁⨁	⨁⨁⨁⨁	⨁⨁⨁⨁
Yildiz et al.^54^	Low	Unclear	Unclear	Unclear	Low	High	Low	3	⨁⨁⨁◯	⨁⨁⨁⨁	⨁⨁⨁⨁	⨁⨁⨁◯	⨁⨁⨁⨁	⨁⨁⨁◯
Dal Prato et al.^55^	Low	Low	High	High	Low	Low	Low	3	⨁⨁⨁⨁	⨁⨁⨁⨁	⨁⨁⨁⨁	⨁⨁⨁⨁	⨁⨁⨁⨁	⨁⨁⨁⨁
Propst et al.^56^	Unclear	Unclear	High	High	Low	Low	Low	3	⨁⨁◯◯	⨁⨁⨁◯	⨁⨁⨁⨁	⨁⨁⨁⨁	⨁⨁⨁⨁	⨁⨁⨁◯
Chakravarty et al.^57^	Unclear	Unclear	Low	Unclear	Unclear	Low	Low	2	⨁⨁⨁◯	⨁⨁⨁◯	⨁⨁⨁⨁	⨁⨁⨁⨁	⨁⨁⨁⨁	⨁⨁⨁◯
Friedler et al.^58^	Unclear	Unclear	Unclear	Unclear	Unclear	Unclear	Low	2	⨁⨁⨁◯	⨁⨁⨁◯	⨁⨁⨁◯	⨁⨁⨁⨁	⨁⨁⨁⨁	⨁⨁⨁◯
Pouly et al.^59^	Low	Unclear	Unclear	Unclear	Low	Low	Unclear	2	⨁⨁⨁◯	⨁⨁⨁⨁	⨁⨁⨁⨁	⨁⨁⨁⨁	⨁⨁⨁⨁	⨁⨁⨁⨁
Salehpour et al.^60^	Unclear	Unclear	High	High	Low	Low	Low	3	⨁⨁◯◯	⨁⨁⨁◯	⨁⨁⨁◯	⨁⨁⨁⨁	⨁⨁⨁⨁	⨁⨁◯◯
Bergh et al.^61^	Low	Unclear	Unclear	Unclear	Low	Low	Unclear	2	⨁⨁⨁◯	⨁⨁⨁⨁	⨁⨁⨁⨁	⨁⨁⨁◯	⨁⨁⨁⨁	⨁⨁⨁◯
Doody et al.^62^	Low	Low	High	Unclear	Low	Low	Low	2	⨁⨁◯◯	⨁⨁⨁◯	⨁⨁⨁⨁	⨁⨁⨁⨁	⨁⨁⨁⨁	⨁⨁◯◯
Tay et al.^63^	Unclear	Unclear	Unclear	Unclear	Unclear	Unclear	Low	2	⨁⨁◯◯	⨁⨁⨁◯	⨁⨁⨁⨁	⨁⨁⨁◯	⨁⨁⨁⨁	⨁⨁◯◯
Abate et al.^64^	Unclear	Unclear	Unclear	Unclear	Unclear	Low	Low	2	⨁⨁⨁◯	⨁⨁⨁⨁	⨁⨁⨁⨁	⨁⨁⨁⨁	⨁⨁⨁⨁	⨁⨁⨁⨁
Abate et al.^65^	Unclear	Unclear	Low	Low	Unclear	Low	Low	2	⨁⨁⨁◯	⨁⨁⨁◯	⨁⨁⨁⨁	⨁⨁⨁⨁	⨁⨁⨁⨁	⨁⨁⨁◯
Aboulghar et al.^66^	Low	Low	Low	Low	Low	Low	Low	1	⨁⨁⨁⨁	⨁⨁⨁◯	⨁⨁⨁⨁	⨁⨁⨁⨁	⨁⨁⨁⨁	⨁⨁⨁⨁
Aghsa et al.^67^	Low	Unclear	Unclear	Unclear	Low	Low	Low	2	⨁⨁⨁⨁	⨁⨁⨁◯	⨁⨁⨁◯	⨁⨁⨁◯	⨁⨁⨁⨁	⨁⨁⨁◯
Ata et al.^68^	Low	Low	Low	Low	Low	Unclear	Low	1	⨁⨁⨁⨁	⨁⨁⨁⨁	⨁⨁⨁⨁	⨁⨁⨁◯	⨁⨁⨁⨁	⨁⨁⨁⨁
Baker et al.^69^	Low	Low	Low	Low	Low	Low	Low	1	⨁⨁⨁⨁	⨁⨁⨁⨁	⨁⨁⨁⨁	⨁⨁⨁⨁	⨁⨁⨁⨁	⨁⨁⨁⨁
Ganesh et al.^70^	Low	High	Low	Low	Low	Low	Low	2	⨁⨁⨁◯	⨁⨁⨁⨁	⨁⨁⨁⨁	⨁⨁⨁⨁	⨁⨁⨁⨁	⨁⨁⨁⨁
Golan et al.^71^	High	Unclear	Low	Low	High	Unclear	Unclear	3	⨁⨁◯◯	⨁⨁⨁◯	⨁⨁⨁◯	⨁⨁⨁◯	⨁⨁⨁⨁	⨁⨁◯◯
Inamdar et al.^72^	Low	Low	Unclear	Unclear	Low	High	Low	2	⨁⨁⨁⨁	⨁⨁⨁⨁	⨁⨁⨁◯	⨁⨁◯◯	⨁⨁⨁⨁	⨁⨁⨁◯
Lockwood et al.^73^	Low	Low	Unclear	Low	Low	Low	Low	1	⨁⨁⨁⨁	⨁⨁⨁◯	⨁⨁⨁⨁	⨁⨁⨁⨁	⨁⨁⨁⨁	⨁⨁⨁⨁
Martinez et al.^74^	Low	Low	Low	Unclear	Unclear	Unclear	Low	2	⨁⨁⨁⨁	⨁⨁⨁◯	⨁⨁⨁◯	⨁⨁⨁◯	⨁⨁⨁⨁	⨁⨁⨁◯
Patki et al.^75^	Unclear	Unclear	Unclear	Unclear	Unclear	High	Unclear	3	⨁⨁⨁◯	⨁⨁◯◯	⨁⨁⨁◯	⨁⨁◯◯	⨁⨁⨁⨁	⨁◯◯◯
Stadtmauer et al.^76^	Low	High	Low	High	Unclear	Low	Unclear	3	⨁⨁◯◯	⨁⨁◯◯	⨁⨁◯◯	⨁⨁⨁◯	⨁⨁⨁⨁	⨁◯◯◯
Tesarik et al.^77^	Low	Low	Low	High	Low	High	Unclear	3	⨁⨁⨁◯	⨁⨁◯◯	⨁⨁⨁◯	⨁⨁◯◯	⨁⨁⨁⨁	⨁◯◯◯
Tournaye et al.^78^	Low	Low	Low	Low	Low	Low	Low	1	⨁⨁⨁⨁	⨁⨁⨁⨁	⨁⨁⨁⨁	⨁⨁⨁⨁	⨁⨁⨁⨁	⨁⨁⨁⨁
Michnova et al.^79^	High	Unclear	Low	Low	Unclear	Low	Low	2	⨁⨁⨁◯	⨁⨁◯◯	⨁⨁⨁◯	⨁⨁⨁◯	⨁⨁⨁⨁	⨁⨁◯◯
Elgindy et al.^80^	Low	Low	High	Unclear	Unclear	Low	Low	2	⨁⨁⨁◯	⨁⨁⨁◯	⨁⨁◯◯	⨁⨁⨁◯	⨁⨁⨁⨁	⨁⨁◯◯
Yang et al.^81^	Low	Low	Low	Low	Low	Low	Low	1	⨁⨁⨁⨁	⨁⨁⨁⨁	⨁⨁⨁⨁	⨁⨁⨁⨁	⨁⨁⨁⨁	⨁⨁⨁⨁
Tomic et al.^82^	Low	Low	Low	Low	Low	Low	Low	1	⨁⨁⨁⨁	⨁⨁⨁⨁	⨁⨁⨁⨁	⨁⨁⨁⨁	⨁⨁⨁⨁	⨁⨁⨁⨁
Gizzo et al.^83^	Low	Low	Low	Low	Low	Low	Low	1	⨁⨁⨁⨁	⨁⨁⨁⨁	⨁⨁⨁⨁	⨁⨁⨁⨁	⨁⨁⨁⨁	⨁⨁⨁⨁
Kutlusoy et al.^84^	High	Unclear	Unclear	Unclear	Low	Low	Low	2	⨁⨁◯◯	⨁⨁⨁◯	⨁⨁⨁◯	⨁⨁⨁◯	⨁⨁⨁⨁	⨁⨁◯◯
Ozer et al.^85^	Low	Low	Low	Low	Low	Low	Low	1	⨁⨁⨁⨁	⨁⨁⨁⨁	⨁⨁⨁⨁	⨁⨁⨁⨁	⨁⨁⨁⨁	⨁⨁⨁⨁
Saharkhiz et al.^86^	Low	Low	Low	Low	Low	Low	Low	1	⨁⨁⨁⨁	⨁⨁⨁⨁	⨁⨁⨁⨁	⨁⨁⨁⨁	⨁⨁⨁⨁	⨁⨁⨁⨁
Horowitz et al.^87^	Moderate	Moderate	Low	Low	Low	Low	Low	1	⨁⨁⨁◯	⨁⨁⨁⨁	⨁⨁⨁⨁	⨁⨁⨁⨁	⨁⨁⨁⨁	⨁⨁⨁⨁
Belaisch-Allart et al.^88^	Low	Moderate	High	High	Moderate	Moderate	Low	3	⨁⨁◯◯	⨁⨁◯◯	⨁⨁⨁◯	⨁⨁⨁◯	⨁⨁⨁⨁	⨁⨁◯◯
Chi et al.^89^	Low	Low	Low	Low	Low	Low	Low	1	⨁⨁⨁⨁	⨁⨁⨁⨁	⨁⨁⨁⨁	⨁⨁⨁⨁	⨁⨁⨁⨁	⨁⨁⨁⨁
Fusi et al.^90^	Low	Low	Low	Low	Low	Low	Low	1	⨁⨁⨁⨁	⨁⨁⨁⨁	⨁⨁⨁⨁	⨁⨁⨁⨁	⨁⨁⨁⨁	⨁⨁⨁⨁
Gorkemli et al.^91^	Low	Moderate	Moderate	Moderate	Low	Low	Low	2	⨁⨁⨁◯	⨁⨁⨁◯	⨁⨁⨁◯	⨁⨁⨁◯	⨁⨁⨁⨁	⨁⨁⨁◯
Ibrahem et al.^92^	Low	Low	Low	Low	Low	Low	Low	1	⨁⨁⨁⨁	⨁⨁⨁⨁	⨁⨁⨁⨁	⨁⨁⨁⨁	⨁⨁⨁⨁	⨁⨁⨁⨁
Kapur et al.^93^	Moderate	Moderate	Moderate	Moderate	High	Moderate	Low	3	⨁⨁◯◯	⨁⨁⨁◯	⨁⨁⨁◯	⨁⨁⨁◯	⨁⨁⨁⨁	⨁⨁◯◯
Khrouf et al.^94^	Moderate	Moderate	Low	Low	Low	Moderate	Low	2	⨁⨁⨁◯	⨁⨁⨁⨁	⨁⨁⨁⨁	⨁⨁◯◯	⨁⨁⨁⨁	⨁⨁⨁⨁
Kwon et al.^95^	Low	Moderate	Low	Low	Low	Moderate	Low	2	⨁⨁⨁◯	⨁⨁⨁◯	⨁⨁⨁⨁	⨁⨁◯◯	⨁⨁⨁⨁	⨁⨁⨁⨁
Mele et al.^96^	Moderate	Moderate	Moderate	Moderate	Low	Low	Low	2	⨁⨁⨁◯	⨁⨁⨁◯	⨁⨁⨁◯	⨁⨁⨁⨁	⨁⨁⨁⨁	⨁⨁⨁◯
Zargar et al.^97^	Moderate	Moderate	Moderate	Moderate	Moderate	Moderate	Low	2	⨁⨁⨁◯	⨁⨁⨁◯	⨁⨁⨁◯	⨁⨁⨁◯	⨁⨁⨁⨁	⨁⨁⨁◯
Pirard et al.^98^	Moderate	Moderate	Moderate	Low	Low	Low	Low	1	⨁⨁⨁◯	⨁⨁⨁◯	⨁⨁⨁⨁	⨁⨁⨁⨁	⨁⨁⨁⨁	⨁⨁⨁◯
Var et al.^99^	Low	Low	Low	Low	Low	Low	Low	1	⨁⨁⨁⨁	⨁⨁⨁⨁	⨁⨁⨁⨁	⨁⨁⨁⨁	⨁⨁⨁⨁	⨁⨁⨁⨁
Humaidan^100^	Low	Low	Low	Low	Low	Low	Low	1	⨁⨁⨁⨁	⨁⨁⨁⨁	⨁⨁⨁⨁	⨁⨁⨁⨁	⨁⨁⨁⨁	⨁⨁⨁⨁

## Results

### Included study design and quality of evidence assessment

From 1322 records initially retrieved, 76 RCTs, comparing 22 interventions of at least two arms comparing LPS protocols in fresh IVF/ICSI cycles, met the inclusion criteria (Fig. 1, Table 1)^25–100^. Overall risk-of-bias judgement was deemed “low” for 24 studies “some concerns” for 29 and “high” for 23 studies (Table 2). Overall, GRADE confidence in evidence was deemed “high” for 34 studies, “moderate” for 23 studies and “low” or “very low” for 19 studies (Table 2).

### Participant and treatment characteristics

A total of 26,536 participants were randomly assigned to any of the following 22 treatments; placebo (no exposure)[N = 727], SCP (Subcutaneous progesterone) [N = 877], VP (vaginal progesterone) [N = 13862], IMP + VP (intramuscular progesterone and vaginal progesterone) [N = 475], VP + OE (vaginal progesterone and oral estradiol) [N = 898], IMP (intramuscular progesterone) [N = 2136], VP + PatchE (vaginal progesterone and patch estrogen) [N = 179], IMP + OE (intramuscular progesterone and oral estradiol) [N = 387], IMHCG (intramuscular hCG) [N = 592], SCP + VP [N = 78], Intranasal GnRH-a [N = 23], OP (oral progesterone) [N = 3693], IMP + IME (intramuscular progesterone and intramuscular estradiol) [N = 55], IMP + VP + OE (Intramuscular progesterone, vaginal progesterone and oral estradiol) [N = 249], IMP + VE (Intramuscular progesterone and vaginal estradiol) [N = 174], VP + SCGNRH-a [(Vaginal progesterone and subcutaneous GNRH agonist (GNRH-a)] [N = 1008], VP + OE + SCGNRH-a (Vaginal progesterone, oral estradiol and subcutaneous GNRH-a) [N = 386], RP (Rectal progesterone) [N = 168], SCHCG (subcutaneous HCG) [N = 160], VP + DHEA (vaginal progesterone and oral DHEA)[N = 104] and IMP + VP + SCGNRH-a (Intramuscular progesterone, vaginal progesterone and subcutaneous GNRH-a) [N = 213] and OP + VP (oral progesterone and vaginal progesterone) [N = 92] (Fig. 2A).

Median participant age across all treatment groups was 32 years [IQR 31.75, 33.85] (Fig. 2B) and the median BMI was 23.94 (kg/m^2^) [IQR 22.45, 26.8] (Fig. 2B,C). Duration of infertility was of a median of 4.96 years [IQR 3.98, 6.10] (Fig. 3A). The population percentage diagnosed with primary infertility was 29.6% [Range: 10.9 to 42.62%] and secondary infertility was 34.5% [Range: 16.1 to 84.65%] and were not found to significantly differ across comparator groups (Fig. 3A–C). Median values of basal AMH, LH, FSH, progesterone levels on HCG trigger, progesterone levels on embryo transfer (ET) day, and endometrial thickness on ET day, per LPS were not found to be significantly different in comparison to the VP group (Figs. 3D–F, 4A–C). Regarding OS protocol, 54.69% of the participants underwent ovarian stimulation with a standard (long) GnRH agonist while 18.17% with a standard (short) GnRH antagonist protocol. A 1.05% underwent OS via clomiphene and HMG, 0.96% via a microdose flare and 0.54% by an ultrashort GnRH protocol (Fig. 4D)^101^. The remaining 24.59% of the participants underwent either a standard long or short OS protocol however the distribution was not noted in the original studies. Characteristics of embryo transfers were not consistently reported across arms of included studies (Table S.11). Of note, 20 of the 76 studies, failed to report upon these variables.

Regarding LPS protocols, schemas were segregated by active compound to explore variations of dosage (median dosage and maximum dosage), initiation day, duration of LPS (weeks) as well route of administration (Tables 1, 3). The majority of LPS protocols were initiated on the oocyte pickup day (OPU), and duration of 8 weeks (SD = 2). No significant differences were noted amongst LPS protocols regarding implantation 24.55% [IQR 18.17, 28.9] or fertilisation 63.6% [IQR 61, 78.9] median rates (Fig. S1).

**Table 3:** LPS protocol characteristics per compound.

Part of Intervention	Compound	Brand name	Route	Dose (Median)	Dose (Max)	Median start of treatment	Median start of treatment SD (days)	Median end of treatment (Weeks)	Median SD end of Treatment (weeks)	Total Number of Patients
VP; IM + VP; VP + OE; VP + PatchE; IMP + VP + OE; IMP + VE; VP + SCGNRH; VP + DHEA; IMP + VP + SCGNRH; OP + VP; SCP + VP	Progesterone (Gel)	Crinone 8%	PV	90 mg	270 mg	OPU	1	10	3	9398
	Progesterone (Pessary)	Utrogestan; Cyclogest; Endometrin; Progeffik;Progestan; Prontogest	PV	600 mg	800 mg	OPU	1.5	8	4	11,033
VP	Progesterone (Ring)	-	PV	1000 mg	1000 mg	OPU	1	7	2.5	1540
OP; OP + VP	Progesterone (Tablet)	Duphaston; Utrogestan	O	Duphaston 30 mg; Utrogestan 600 mg	Duphaston 40 mg; Utrogestan 600 mg	OPU	2	12	3.5	3785
RP	Progesterone (Pessary)	Cyclogest	PR	600 mg	800 mg	OPU	0	4	2	168
SCP	Progesterone (Solution)	Prolutex	SC	25 mg	50 mg	OPU	0	10	2	955
IM + VP; IMP + OE; IMP + VP + OE; IMP + VP + OE; IMP + VE; IMP + VP + SCGNRH	Progesterone (Solution)	Progesterone oil; Gestone	IM	100 mg	200 mg	OPU	1	6	3.5	5134
VP + OE; IMP + VP + OE; VP + OE + SCGNRH	Estradiol valerate	Estrofem; Progynova; Cycloprogynova (with norgestrel)	O	4 mg	4 mg	OPU	1	7	4	1920
IMP + IME	Estradiol valerate	Estradiol valerate	IM	20 mg	20 mg	OPU	0	12	0	55
IMP + VE	Estradiol valerate	Estradiol valerate	PV	4 mg	6 mg	OPU	2	6	0	174
VP + PatchE	Estradiol	Estraderm T	Patch	100 mcg	200 mcg	OPU	2	10	1.5	179
IMHCG	HCG	Pregnyl	IM	2000 IU	2500 IU	OPU + 2	2	2	1.2	592
SCHCG	HCG	Profasi	SC	1500 IU	1500 IU	OPU + 2	0	1	0	160
VP + SCGNRH; VP + OE + SCGNRH; IMP + VP + SCGNRH	GnRH	Decapeptyl; Leuprolide acetate; Triptorelin	SC	Decapeptyl 0.1 mg Leuprolide 1 mg Triptorelin 0.1 mg	Decapeptyl 0.1 mg Leuprolide 2 mg Triptorelin 0.1 mg	OPU	0	1 dose	14 doses (daily till bHCG test)	1607
Intranasal GNRG	GnRH	Buserelin	Intranasal	100mcg	200mcg	Ovulation trigger day	0	100 μg IN buserelin TDS for luteal support starting the next day of ovulation trigger up to day 14 of luteal phase	N/A	23
VP + DHEA	DHEA (tablets)	Prasterone	O	75 mg	75 mg	OPU	0	12	0	104

Compound mono- or multi-treatment for luteal support, median and maximum dose, median day of luteal support initiation and median duration of treatment.

OPU, Oocyte retrieval day; ET, Embryo Transfer; placebo, no exposure; SCP, Subcutaneous progesterone; VP, vaginal progesterone; IMP + VP, intramuscular progesterone and vaginal progesterone; VP + OE, vaginal progesterone and oral estradiol; IMP, intramuscular progesterone; VP + PatchE, vaginal progesterone and patch oestrogen; IMP + OE, intramuscular progesterone and oral estradiol; IMHCG, intramuscular hCG; SCP + VP, Intranasal GnRH-a; OP, oral progesterone; IMP + IME, intramuscular progesterone and intramuscular estradiol; IMP + VP + OE, Intramuscular progesterone, vaginal progesterone and oral estradiol; IMP + VE, Intramuscular progesterone and vaginal estradiol; VP + SCGNRH-a, Vaginal progesterone and subcutaneous GNRH agonist (GNRH-a); VP + OE + SCGNRH-a, Vaginal progesterone, oral estradiol and subcutaneous GNRH-a; RP, Rectal progesterone), SCHCG (subcutaneous HCG), VP + DHEA (vaginal progesterone and oral DHEA; IMP + VP + SCGNRH-a, Intramuscular progesterone, vaginal progesterone and subcutaneous GNRH-a; OP + VP, oral progesterone and vaginal progesterone.

### Data synthesis and network meta-analysis

VP was considered as the reference treatment as previously mentioned (NICE guidelines^17^. In NMA, effect size estimates suggested that all LPS protocols were consistently superior to placebo, employed as a negative control for both primary and secondary outcomes, regardless of risk of bias sensitivity analysis (Fig. 5, Fig. S2–S10, Tables S2–S4).

More specifically, regarding NMA primary outcomes:

1. For clinical pregnancy events, reported by 74 studies, CiNeMa NMA RoB rating was deemed “moderate” and overall network incoherence was found to be moderate, *χ*^*2*^ 7.02, 4 degrees of freedom, p-value: 0.005) (Table S5, Fig. 5A, Fig. S2A, Fig. S3A, Fig. S5A, Fig. S6A, Fig. S9A). All LPS protocols appeared to be equivalent to VP in respect to the clinical pregnancy events, except for VP + OE + SCGNRH-a, [OR 1.57 (95% CrI 1.11 to 2.22) (SUCRA: 80%; N_patients_:386, “Moderate” GRADE] and VP + SCGNRH-a [OR 1.28 (95% CrI 1.05 to 1.55) (SUCRA: 80%; N_patients_:583, “High” GRADE], which were found to be superior, with high SUCRA probability (Fig. 5A, Fig. S5A, S6A). Equally, VP + PatchE was also associated with higher clinical pregnancy probability, OR 1.73 (95% CrI 1.16, 2.58) (SUCRA: 79%; N_patients_:179, “Moderate” GRADE). Treatments such as IMP + IME OR 2.68 (95% CrI 1.06, 7.72) (SUCRA: 90%; N_patients_:55, “Low” GRADE) and were shown to be superior in comparison to VP however the certainty in evidence was deemed low given the small number of participants included and the high risk of subsequent heterogeneity.
2. For the live pregnancy events, reported by 43 studies, CiNeMa NMA RoB rating was deemed “moderate” and overall network incoherence was found to be moderate, *χ*^*2*^ 10.95 (5 degrees of freedom), *p* value: 0.052 (Table S6, Fig. 5B, Fig. S2B, Fig. S3B, Fig. S5B, Fig. S6B, Fig. S9B). The following interventions were found to improve live pregnancy events in comparison to the reference LPS, IMHCG [OR 9.67 (95% CrI 2.34 to 73.2) (SUCRA: 92%; N_patients_:592, “Moderate” GRADE)], VP + OE [OR 4.57 (95% CrI 1.26 to 20) (SUCRA: 80%; N_patients_:898, “Moderate” GRADE)], VP + OE + SCGNRH-a OR [OR 8.81 (95% CrI 2.35 to 39.1) (SUCRA: 95%; N_patients_:386, “High” GRADE)], VP + SCGNRH-a [OR 1.76 (95% CrI 1.45 to 2.15) (SUCRA: 72%; N_patients_:1008, “High” GRADE)] (Fig. 5B, Fig. S5B).

Regarding secondary outcomes:

3. For biochemical pregnancy events, reported by 29 studies, CiNeMa NMA RoB rating was deemed “Moderate” and network incoherence was found to be moderate, *χ*^*2*^ 6.60 (2 degrees of freedom), *p* value: 0.037 (Table S7, Fig. 5C, Fig. S2C, Fig. S3C, Fig. S5C, Fig. S6C, Fig. S9C). For VP versus all other LPS protocols. No LPS protocol appeared to result in a significantly higher biochemical pregnancy probability.
4. Regarding miscarriage events, reported by 41 studies, CiNeMa NMA RoB rating was deemed “Moderate” and network incoherence was found to be moderate, *χ*^*2*^ 11.30 (4 degrees of freedom), *p* value: 0.023 (Table S9, Fig. 5D, Fig. S2D, Fig. S4A, Fig. S5D, Fig. S6D, Fig. S9D). VP + SCGnRH-a was found to reduce miscarriage events in comparison to the reference LPS, [OR 0.54 (95% CrI 0.372 to 0.806), N_patients_:1008, “Moderate” GRADE] with a SUCRA of 82.2% (Fig. 5D, Fig. S4A, Fig. S5D). Additionally, a similar finding was confirmed for IMP + IME [OR 0.08 (95% CrI 0.01 to 0.46), N_patients_:55] however the certainty in evidence was deemed “Low”.
5. For multiple pregnancy events, reported by 21 studies, CiNeMa NMA RoB rating was deemed “High” (Table S9). Overall network incoherence was found to be low, *χ*^*2.*^ 0.115 (2 degrees of freedom), *p* value: 0.94 (Fig. 5E, Fig. S2E, Fig. S4B, Fig. S5E, Fig. S6E, Fig. S9E). All LPS protocols appeared to produce similar results to PVP, except for SCP [OR 0.09 (95% CrI 0.009 to 0.556); SUCRA 1.2%, N_patients_:877, “High” GRADE] resulting to significantly lower multiple pregnancy events and IMP + VP + SCGNRH-a [OR 6.88 (95% CrI 2.42 to 30.4); SUCRA 81.2%, N_patients_:213, “Low” GRADE] resulting in significantly higher multiple pregnancy events.
6. For OHSS events, reported by 15 studies, CiNeMa NMA RoB confidence rating was deemed “Low” (Table S10). Overall network incoherence was found to be low, *χ*^*2.*^: 0.26 (2 degrees of freedom), *p* value: 0.015 (Fig. 5F, Fig. S2F, Fig. S5F, Fig. S6F, Fig. S9F). Pairwise analysis of included studies was not feasible due to the multitude of non-events (zero events of OHSS in either of the arms of the original study). All LPS protocols appeared to be associated with similar OHSS events to the reference LPS, except for OP [OR 1.87 (95% CrI 1.15 to 3.04); N_patients_:3693, SUCRA 75%, “Low” GRADE] which was found to be associated with significantly higher OHSS events. The latter is likely to be a result of bias towards an OP LPS protocol selection in patients at high risk of ovarian hyperstimulation^102,103^.

Subgroup analysis of low and medium risk of bias studies (Figs. S7, S8, S10, Table 2) and node-splitting (Table S2–S3) did not significantly alter cumulative effects analysis or residual deviance (Fig. S10A–F). Optimal LPS per OS, long (Gonadotropin releasing hormone agonist) vs. short (GnRH antagonist) protocol, was explored to identify further sources of heterogeneity and to delineate whether a particular LPS appears to yield improved clinical outcomes in association with specific ovarian stimulation protocols (Table 4, Table S4). In view of live birth events, the following protocols were deemed optimal for participants that underwent OS by standard GnRH agonist protocol: (a) VP + OE + SCGNRH-a [OR 9.7 (95% CrI 3.73, 13.5)] (b) VP + OE [OR 4.58 (95% CrI 1.26, 20.3)], (c) VP + SCGNRH-a [OR 2.89 (95% CrI 1.46, 3.42)], and (d) IMHCG [OR 1.57 (95% CrI 2.24, 71.9)]. Of the aforementioned, the VP + OE, VP + SCGNRH-a and IMHCG comparators had a “High” GRADE rating while the VP + OE + SCGNRH-a protocol was also associated with a higher probability of miscarriage when used in combination with a GnRH agonist OS protocol, [OR 3.93 (1.69, 10.1)]. On the contrary, optimal luteal support protocols for standard GnRH antagonist OS were (a) IMHCG [OR 3.2 (95% CrI 1.54, 334.), “low” GRADE] and (b) VP + SCGNRH [OR 2.84 (95% CrI 1.35, 6.24), “High” GRADE] presenting the optimal LPS options across short protocols. Of note, IMHCG was also associated with a higher probability of miscarriage when used in conjunction with a short OS protocol [OR 2.11 (95% CrI 0.75, 6.40), high GRADE] while the opposite held true for VP + SCGNRH, which was associated with lower probability of miscarriage in short OS [OR 0.54 (95% CrI 0.37, 0.80), high GRADE]. Network meta-regression for all outcomes, according to embryological parameters, did not significantly alter effect sizes (Table S11–S12).

**Table 4:** Subgroup analysis of optimal LPS in short (GnRH antagonist) vs. long (GnRH agonist) protocol.

Outcome												
Ovarian stimulation	Clinical Pregnancy		Live Birth		Biochemical Pregnancy		Multiple Pregnancy		Miscarriage		OHSS	
	Long	Short	Long	Short	Long	Short	Long	Short	Long	Short	Long	Short
LPS Protocol												
SCP	1.99 (1.00, 4.03)	1.06 (0.869, 1.31)	0.454 (0.362, 0.572)	N/A	0.928 (0.789, 1.09)	0.928 (0.789, 1.09)	0.0942 (0.0106, 0.549)		1.37 (0.0994, 48.4)	0.842 (0.444, 1.59)	N/A	Disconnected Network
Placebo	0.707 (0.489, 1.01)	0.385 (0.265, 0.550)	0.101 (0.0145, 0.370)	N/A	0.284 (0.153, 0.495)	0.284 (0.153, 0.495)	N/A		0.135 (0.0279, 0.457	N/A	N/A	
IM + VP	0.871 (0.526, 1.44)	0.966 (0.561, 1.66)	N/A	N/A	N/A	N/A	7.01 (2.33, 29.2)	7.41 (2.59, 33.8)	3.98 (1.93, 8.46	N/A	N/A	
VP + OE	0.943 (0.650, 1.36)	1.51 (1.00, 2.29)	4.58 (1.26, 20.3)	N/A	N/A	N/A	2.47 (0.191, 77.4)	3.69 (0.884, 20.2)	1.16 (0.666, 1.99)	N/A	0.22 (0.12 − 0.97)	
IMP	1.06 (0.896, 1.26)	0.969 (0.797, 1.17	1.06 (0.784, 1.43)	1.07 (0.820, 1.39)	1.86 (1.31, 2.65)	1.86 (1.31, 2.65)	N/A	0.825 (0.640, 1.06)	1.34 (0.626, 2.97)	1.26 (0.671, 2.42)	N/A	
VP + PE	0.992 (0.528, 1.87)	2.35 (1.38, 4.10	N/A	N/A	N/A	N/A	0.801 (0.295, 2.16		3.01 (1.15, 8.98)	N/A	N/A	
IMP + OE	N/A	0.824 (0.598, 1.13	N/A	N/A	N/A	N/A	N/A	0.852 (0.446, 1.61	N/A	N/A	N/A	
IMHCG	0.801 (0.578, 1.10)	2.08 (1.09, 6.32)	1.57 (2.24, 71.9)	3.2 (1.54, 334.)	N/A	N/A	N/A		0.846 (0.423, 1.70	N/A	1.64 (0.749, 3.73)	
OP	0.963 (0.833, 1.11)	0.980 (0.869, 1.11)	1.23 (1.07, 1.42)	1.06 (0.729, 1.54	1.61 (0.806, 3.22)	1.61 (0.806, 3.22)	0.679 (0.275, 1.62)		0.879 (0.652, 1.18)	2.11 (0.754, 6.40)	2.68 (2.11, 2359)	
IMP + IME	N/A	1.96 (0.734, 5.59)	1.06 (0.784, 1.43)	N/A	0.00975 (0.000295, 0.0	0.00975 (0.0002, 0.0668)	N/A		N/A	N/A	N/A	
IMP + VP + OE	N/A	1.12 (0.655, 1.91)	N/A	N/A	N/A	N/A	N/A		2.35 (1.04, 5.36)	N/A	N/A	
IMP + VE	N/A	0.990 (0.638, 1.55)	N/A	N/A	N/A	N/A	N/A		N/A	N/A	N/A	
VP + SCGNRH	1.13 (0.874, 1.46)	2.05 (1.08, 2.96)	2.89 (1.46, 3.42)	2.84 (1.35, 6.26)	1.91 (0.974, 3.74)	1.91 (0.974, 3.74)	1.09 (0.638, 1.89)	8.34 (2.57, 37.6)	N/A	0.549 (0.376, 0.804	N/A	
VP + OE + SCGNRH	1.25 (0.743, 2.08)	2.12 (1.25, 3.62)	9.7 (3.73, 13.5)	N/A	N/A	N/A	N/A		3.93 (1.69, 10.1)	N/A	N/A	
RP	3.23 (2.36, 40.8)	0.754 (0.385, 1.47)	N/A	N/A	0.775 (0.486, 1.23)	0.775 (0.486, 1.23)	N/A	0.650 (0.0737, 4.46	N/A	N/A	N/A	
SCHCG	3.13 (2.30, 40.8	N/A	N/A	N/A	N/A	N/A	N/A		N/A	N/A	N/A	
VP + DHEA	N/A	0.958 (0.534, 1.72)	N/A	N/A	N/A	N/A	N/A		N/A	N/A	N/A	
IMP + VP + SCGNRH	0.817 (0.430, 1.56)	N/A	N/A	N/A	N/A	N/A	6.82 (2.29, 28.8)	7.21 (2.51, 32.7)	N/A	N/A	N/A	
OP + VP	N/A	0.101 (0.0425, 0.210)	N/A	N/A	0.0692 (0.0241, 0.166)	0.0692 (0.0241, 0.166)	N/A		N/A	N/A	N/A	
SCP + VP	N/A	0.0457 (0.0105, 0.130)	N/A	N/A	0.113 (0.0445, 0.256)	0.113 (0.0445, 0.256)	N/A		N/A	N/A	N/A	
IN + GNRH	N/A	1.87 (0.454, 10.2)	N/A	N/A	N/A	N/A	N/A		N/A	N/A	N/A	

no exposure, placebo; SCP, Subcutaneous progesterone; VP, vaginal progesterone; IMP + VP, intramuscular progesterone and vaginal progesterone; VP + OE, vaginal progesterone and oral estradiol; IMP, intramuscular progesterone; VP + PatchE, vaginal progesterone and patch oestrogen; IMP + OE, intramuscular progesterone and oral estradiol, IMHCG, intramuscular hCG; SCP + VP, Intranasal GnRH-a; OP, oral progesterone; IMP + IME, intramuscular progesterone and intramuscular estradiol; IMP + VP + OE, Intramuscular progesterone, vaginal progesterone and oral estradiol; IMP + VE, Intramuscular progesterone and vaginal estradiol; VP + SCGNRH-a, Vaginal progesterone and subcutaneous GNRH agonist (GNRH-a); VP + OE + SCGNRH-a, Vaginal progesterone, oral estradiol and subcutaneous GNRH-a); RP, Rectal progesterone; SCHCG, subcutaneous HCG; VP + DHEA, vaginal progesterone and oral DHEA; IMP + VP + SCGNRH-a, Intramuscular progesterone, vaginal progesterone and subcutaneous GNRH-a; OP + VP, oral progesterone and vaginal progesterone; Short, standard GnRH antagonist protocol; Long, standard GnRH agonist protocol for ovarian stimulation.

Overall, NMA data suggest that combinatorial treatments, with the addition of SCGNRH-a on a VP base results in improved clinical pregnancy and live birth events and reduced miscarriage events in participants undergoing OS either a standard GnRH antagonist or agonist protocol. However, participants undergoing a long GnRH protocol OS appear to benefit more from IMHCG as LPS while participants undergoing a short GnRH protocol OS appear to benefit more from VP + SCGNRH, considering the reduction of miscarriage events of these luteal support protocols in conjunction to OS.

## Discussion

This study is based on 76 RCTs, including 26,536 participants randomly assigned to 22 LPS protocols including non-exposure. Given the plethora of previous data suggesting that any LPS protocol is superior to non-exposure, the most widely employed LPS, vaginal progesterone, was set as a reference treatment^3,17^. Overall, meta-synthesized data presented here, suggest that combinatorial treatments, those with the addition of SCGnRH on a VP base result in improved clinical pregnancy, OR 1.28 (95% CrI 1.05 to 1.55) and live birth events, OR 1.76 (95% CrI 1.45 to 2.15) with high confidence in evidence. Of note, addition of oral estradiol to a VP + SCGNRH-a LPS, resulted in further improvement of clinical pregnancy events by 29% and 44% increase of a clinical pregnancy and live birth odds respectively. Of note, participants undergoing a long GnRH protocol OS appeared to benefit more from progesterone free LPS such as IMHCG in view of increased live birth, OR 1.57 (95% CrI 2.24 to 71.9) and reduced miscarriage events, OR 1.57 (95% CrI 2.24 to 71.9). However, participants appeared to be at a higher risk of OHSS, OR 1.64 (95% CrI 0.74 to 3.73). On the other hand, participants undergoing a short GnRH OS protocol appeared to benefit more from VP + SCGNRH with a live birth OR 2.84 (95% CrI 1.35 to 6.26), however while the probability of miscarriage was significantly reduced, OR 0.55 (95% CrI 0.38 to 0.80), the probability of multiple pregnancy significantly increased, OR 8.34 (95% CrI 2.57 to 37.6).

Luteal support is a critical aspect of IVF/ICSI cycles as it aids in maintaining the endometrial lining, in turn promoting embryo implantation, and supporting early pregnancy. In fresh IVF cycles, luteal support management can pose several challenges, including timing and duration of administration, individual outcome variability and tolerability of LPSs that may impact upon the success rates of the cycle. The effectiveness of luteal support in achieving live birth and clinical pregnancy rates is dependent on the timing of its administration^104–106^. Various studies have examined the optimal timepoint to initiate LPS, with only two out of five RCTs reporting statistically significant results^104^. Earlier evidence had suggested that delayed administration of LPS [(24 h after ovum pick-up (OPU)] may be more advantageous than pre-OPU administration (12 h prior to OPU)^106^. Williams et al. found initiating LPS on day 3 post OPU to be significantly better than delaying it until day 6^105^. Overall, these studies suggest that the optimal time for LPS administration is from the evening of OPU up until 3 days post OPU. Present NMA evidence suggested that the majority of studies favoured LPS initiation on the day of OPU (within the 24 h timeframe following the procedure), including for LPS protocols generating superior results namely, VP + SCGNRH-a and VP + OE + SCGNRH-a. Equally important to the LPS initiation timing, is the duration of luteal support administration. A recent meta-analysis including 1297 participants, indicating that continuing progesterone for two weeks after a positive pregnancy test did not have any significant impact on miscarriage or delivery rates^106^. The same study suggested that it is unnecessary to continue LPS for up to 10 weeks of pregnancy with further studies reaching to the same conclusion^107–109^. However, ESHRE 2020 recommendations suggest that LPS should be administered up until, at least the day of the pregnancy test^101^. Aggregate evidence of the present study indicate that duration of administration is highly dependent upon the selected LPS regimen, with an overall median of 8 weeks [Range 2–12] coinciding with ultrasonographic evidence of fetal motion and the concept of the luteo-placental shift^110,111^.

In addition, while initiation and duration of LPS treatment may appear more standardised, the selection of optimal type and dose of luteal support is largely individualised and dependent upon participant factors such as age, BMI, and reproductive history. Regardless of clinical and demographic parameters, undoubedtly the most important parameter affecting LPS selection and duration of perscription, is fundamentaly influenced by patient preference, which is in turn heavily reliant upon LPS side effect profile and tolerability, patient compliance and cost. For example, in view of treatment acceptability, IM progesterone has been widely available prior to vaginal formulation becoming available, and has been shown to have superior absorption and achieve stable serum concentration shortly after administration^109,112,113^. Nonetheless, administration complications involving pain, higher risk of infection, sterile abscess formation, and even rarely eosinophilic pneumonia as well as practical impediments requiring daily visits and injections, have necessitated the exploration of alternative more convinient routes, such as the one offered by the vaginal preparation^114^. Currently, vaginal progesterone products are administered in various ways, including pessaries, capsules, tablets, gel, and inserts which can achieve maximum serum concentration of progesterone after 3–8 h of administration, and by daily doses of 300–600 mg may achieve adequate available plasma levels^115^. Evidence has also shown that a 300–600 mg of vaginal micronized progesterone daily can induce similar endometrial maturation as 100 mg intramuscular progesterone daily^109^. By enabling direct transport of "first uterine pass" progesterone from the vagina to the uterus, vaginal preparations achieve adequate tissue levels of progesterone with lower circulating levels, indicating acceptable bioavailability^111^.

Given the improved outcomes regarding clinical pregnancy and live birth, achieved by VP + SCGNRH-a and VP + OE + SCGnRH-a combinatorial treatments, shown in the present work, a mention to route and dosage of gonadotropin-releasing hormone agonist is warranted. The use of GnRH-a for LPS was suggested following accidental use of GnRH agonist during this phase which resulted in improved implantation rates^116^. The effect of GnRH agonist has been observed at three levels: support of the corpus luteum through pituitary LH secretion, direct effects on the embryo and implantation process, and the effect upon trophectoderm cells and endometrial GnRH receptors^77,116,117^. A meta-analysis showed that administering a single dose of GnRH-a increased the implantation rate in cycles with GnRH antagonist and long GnRH-a protocols, clinical pregnancy rate per transfer, and ongoing pregnancy rate^118^, whilst another revealed that the use of GnRH-a for LPS significantly improved live birth rate, clinical pregnancy rate, and ongoing pregnancy rate^119^. An additional study demonstrated that a single dose of GnRH-a had similar efficacy as three doses of hCG^120^. One can hypothesise that the addition of a GnRH agonist can bimodally support the corpus luteum by stimulating the release of gonadotrophins from the pituitary gland, and by directly influencing the endometrium through interaction with GnRH receptors. Furthermore, research suggests that administering a single dose of GnRH agonist during the luteal phase enhances rates of pregnancy, implantation, delivery, and birth among recipients of donated oocytes whose ovulation was suppressed and corpus luteum was absent, suggesting a potential direct impact of GnRH agonist on the embryo^77,98,116,119^. The present work has highlighted that a single SCGNRH administration in addition to a VP protocol, can positively impact on IVF/ICSI outcomes especially in patients undergoing GnRH antagonist OS and could be reserved for more challenging cycles to optimise results. Conversely, in view of the improved clinical pregnancy and live birth outcomes achieved by the addition of oral estradiol in the VP + SCGNRH-a protocol, exploration of the possible synergistic effects of this compound is necessitated. However, a Cochrane meta-analysis did not find evidence to support routinely administering estrogen with progesterone in IVF cycles^9^. In antagonist cycles, progesterone levels surge, leading to a rebound decrease in serum estradiol, which in turn has formulated the hypothesis that adding doses of 2–6 mg/day of estradiol could be beneficial^111^. However, contemporary systematic reviews failed to confirm the beneficial effects of oral or any route of estradiol addition to progesterone LPS upon pregnancy outcomes^120–122^. Of note, novel LPS regimens involving intranasal GnRH administration has been shown promising results regarding clinical pregnancy rates and treatment tolerability however given the scarse RCT evidence, further, adequately powered, RCTs would be required to allow recommendations regarding this LPS regimen^117,123^.

In addition, the present work has shown that progesterone free LPS protocols, such as intramuscular hCG, may be equal, if not more effective that progesterone-based LPS in view of live birth outcomes, especially in patients undergoing a GnRH agonist OS protocol. HCG, by mimicking LH pulsatility, was initially considered the primary choice for LPS as it stimulates the corpus luteum to produce progesterone continuously. However, this approach has drawbacks, as it can elevate the risk of OHSS, a hypothesis which was also confirmed by the present NMA, albeit lacking statistical significance, OR 1.64 [95% CrI 0.75, 3.71].

## Limitations and future perspectives

The optimal protocol for luteal support is a constantly evolving field of research in artificial reproduction. In view of the plethora of available LPS protocols, NMA precision of estimates provides a more comprehensive understanding of the comparative effectiveness of different protocols. In the present work, only RCT data have been employed to reach meaningful conclusions limiting inherent bias of diverse participant populations, with add-on sensitivity analysis targeted at low and moderate risk of bias studies and ovarian stimulation protocol to further explore confounding factors and detect sources of heterogeneity. Given the anticipated diversity of measured outcomes, a bayesian meta-synthesis approach has been adopted to account for the expected heterogeneity and to incorporate modelling flexibility by allowing for posterior distributions interpreted as SUCRA probabilities with the later enabling crisper communication of the uncertainty in the treatment effects estimates.

On this note, in the present study, LPS protocols have been treated as unique comparator entities, allowing for assessment of selected outcomes on a protocol- rather than a compound-level. However, side effect and safety profile of combinatorial treatments has not been assessed and therefore a significant confounder in tolerability and in turn, compliance, especially in the context of combinatorial LPS, remains to be investigated. Additionally, cost-effectiveness analysis has not been undertaken, which needs to be factored in a joined patient and clinical decision-making. Moreover, while reported, the present study did not aim to clarify of optimal initiation/cessation timing of LPS or the optimal dosage and therefore to produce concrete recommendations regarding these LPS parameters, further studies with relevant designs should be implemented. Notably, included studies were significantly heterogeneous in terms of reporting the characteristics of embryo transfers. Abeit no statistical difference was reported for variables such as follicles retrieved, peak oestradiol levels and number of embryos transferred in individuals studies, confounding effects cannot be confidently excluded. Lastly, OHSS events were found to be considerably under-reported across RCTs with only 15 studies noting such events. Reflecting on the implications of OHSS upon both the patient clinical management as well as the success of the IVF/ICSI, it would be strongly recommended that future RCTs would thoroughly record OHSS events across study arms. Overall, luteal support management in fresh IVF cycles is a complex and dynamic process that calls for careful consideration and individualised LPS selection to achieve optimal outcomes.

## Conclusion

Herein meta-synthesized data suggest that combinatorial treatments, with the addition of subcutaneous GnRH agonist, on a vaginally administered progesterone LPS base, results in improved clinical pregnancy and live birth events. However, the side-effect and tolerability profile of such combinatorial LPS protocols needs to be thoroughly investigated prior to their wide-scale adoption in clinical practice.

### Supplementary Information

Supplementary Information.

## Data Availability

All data associated with the present study are available in the main body or the supplementary material of the submission. Data regarding any of the subjects in the study has been published in the form of randomised control studies. Crude data were extracted and homogenized for the purposes of the present systematic review and network meta-analysis. All included studies have been referenced as required.
