# Supplementary material for: Comparison of luteal support protocols in fresh IVF/ICSI cycles: a network meta-analysis
Source: Sci Rep. 2024 Jun 24;14:14492. doi: 10.1038/s41598-024-64804-z (PMC11196689; doi:10.1038/s41598-024-64804-z)
Supplement: Supplementary file 1 — Supplementary Information. [file 41598_2024_64804_MOESM1_ESM.docx]

**Title.** Comparison of luteal support protocols in fresh IVF/ICSI cycles: A network meta-analysis”

**Authors**. Stavroula L. Kastora, PhD ^1,2^; Grigoria Gkova, MBChB^2^; Konstantinos Stavridis, MBChB^3^; Neerujah Balachandren^1^; Athanasios Kastoras, PhD^4^; Andreas Karakatsanis, PhD^5,6^; Dimitrios Mavrelos, PhD^1^

Supplementary Figures and Tables

Figures S 1-10

Figure S1. Comparison of reported implantation (A) and fertilisation (B) rates [Median, 95%CI] per luteal support protocol. Only comparisons that reached statistical significance are depicted. The reference group was VP. Two decimal p values and asterisk annotation of significance where p-value <0.05, it is flagged with one star (*), p-value < 0.01, 2 stars (**), p-value < 0.001, three stars (***), p-value < 0.0001, four stars (****). Abbreviations: placebo (no exposure), SCP (Subcutaneous progesterone), VP (vaginal progesterone), IMP+VP (intramuscular progesterone and vaginal progesterone), VP+OE (vaginal progesterone and oral estradiol), IMP (intramuscular progesterone), VP+PatchE (vaginal progesterone and patch oestrogen), IMP+OE (intramuscular progesterone and oral estradiol), IMHCG (intramuscular hCG), SCP+VP, Intranasal GnRH-a, OP (oral progesterone), IMP+IME (intramuscular progesterone and intramuscular estradiol), IMP+VP+OE (Intramuscular progesterone, vaginal progesterone and oral estradiol), IMP+VE (Intramuscular progesterone and vaginal estradiol), VP+SCGNRH-a [(Vaginal progesterone and subcutaneous GNRH agonist (GNRH-a)], VP+OE+SCGNRH-a (Vaginal progesterone, oral estradiol and subcutaneous GNRH-a), RP (Rectal progesterone), SCHCG (subcutaneous HCG), VP+DHEA (vaginal progesterone and oral DHEA), IMP+VP+SCGNRH-a (Intramuscular progesterone, vaginal progesterone and subcutaneous GNRH-a), OP+VP (oral progesterone and vaginal progesterone).


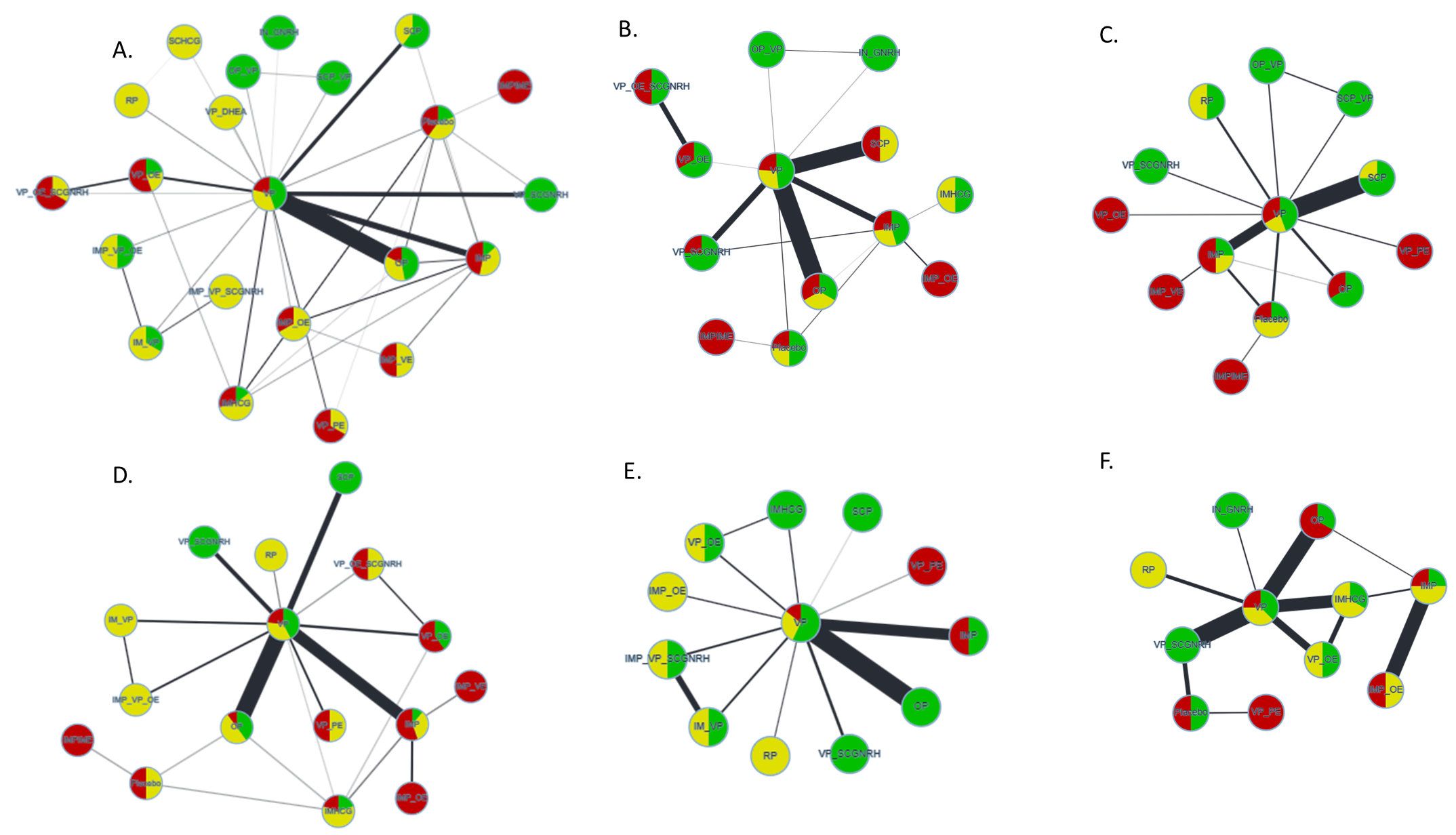


Figure S2. Network of comparisons per outcome, Clinical Pregnancy (A), Live Birth (B), Biochemical pregnancy (C), miscarriage (D), multiple pregnancy ( E) OHSS (F). The colours in the circles indicate the percentage of low RoB studies [green], moderate RoB studies [yellow] and high RoB studies [red] involving each luteal support protocol. The thickness of each edge (connective line) indicates the sample size in each comparison. Abbreviations: placebo (no exposure), SCP (Subcutaneous progesterone), VP (vaginal progesterone), IMP+VP (intramuscular progesterone and vaginal progesterone), VP+OE (vaginal progesterone and oral estradiol), IMP (intramuscular progesterone), VP+PatchE (vaginal progesterone and patch oestrogen), IMP+OE (intramuscular progesterone and oral estradiol), IMHCG (intramuscular hCG), SCP+VP, Intranasal GnRH-a, OP (oral progesterone), IMP+IME (intramuscular progesterone and intramuscular estradiol), IMP+VP+OE (Intramuscular progesterone, vaginal progesterone and oral estradiol), IMP+VE (Intramuscular progesterone and vaginal estradiol), VP+SCGNRH-a [(Vaginal progesterone and subcutaneous GNRH agonist (GNRH-a)], VP+OE+SCGNRH-a (Vaginal progesterone, oral estradiol and subcutaneous GNRH-a), RP (Rectal progesterone), SCHCG (subcutaneous HCG), VP+DHEA (vaginal progesterone and oral DHEA), IMP+VP+SCGNRH-a (Intramuscular progesterone, vaginal progesterone and subcutaneous GNRH-a), OP+VP (oral progesterone and vaginal progesterone).


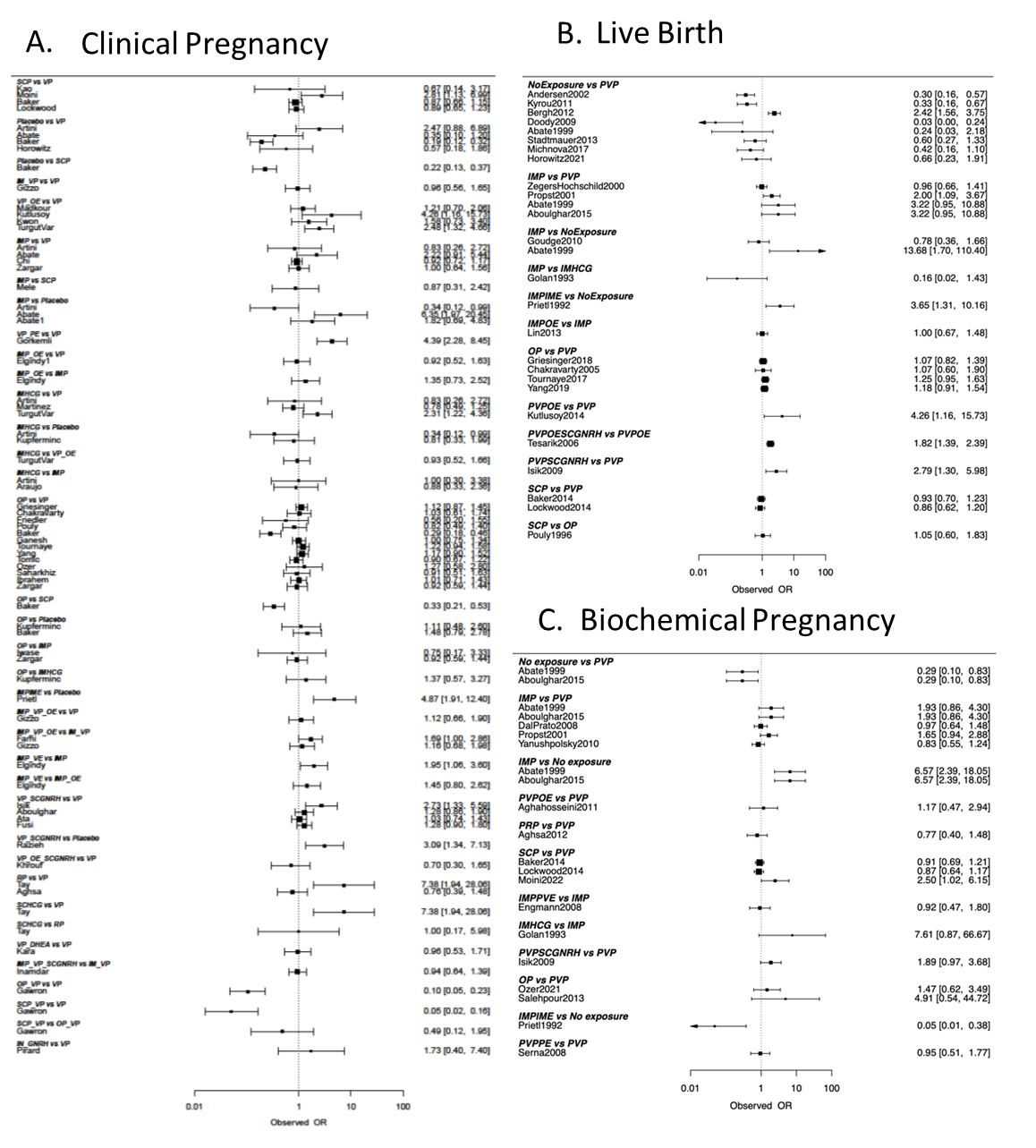


Figure S3. Pairwise comparisons of luteal support protocols measuring clinical pregnancy rate (A), live birth rate (B) and biochemical pregnancy (C) outcomes. Maentel Haensel Odds ratio (95% CI) forest plot generated with MetaInsight freeware package. Abbreviations: placebo (no exposure), SCP (Subcutaneous progesterone), VP (vaginal progesterone), IMP+VP (intramuscular progesterone and vaginal progesterone), VP+OE (vaginal progesterone and oral estradiol), IMP (intramuscular progesterone), VP+PatchE (vaginal progesterone and patch oestrogen), IMP+OE (intramuscular progesterone and oral estradiol), IMHCG (intramuscular hCG), SCP+VP, Intranasal GnRH-a, OP (oral progesterone), IMP+IME (intramuscular progesterone and intramuscular estradiol), IMP+VP+OE (Intramuscular progesterone, vaginal progesterone and oral estradiol), IMP+VE (Intramuscular progesterone and vaginal estradiol), VP+SCGNRH-a [(Vaginal progesterone and subcutaneous GNRH agonist (GNRH-a)], VP+OE+SCGNRH-a (Vaginal progesterone, oral estradiol and subcutaneous GNRH-a), RP (Rectal progesterone), SCHCG (subcutaneous HCG), VP+DHEA (vaginal progesterone and oral DHEA), IMP+VP+SCGNRH-a (Intramuscular progesterone, vaginal progesterone and subcutaneous GNRH-a), OP+VP (oral progesterone and vaginal progesterone),


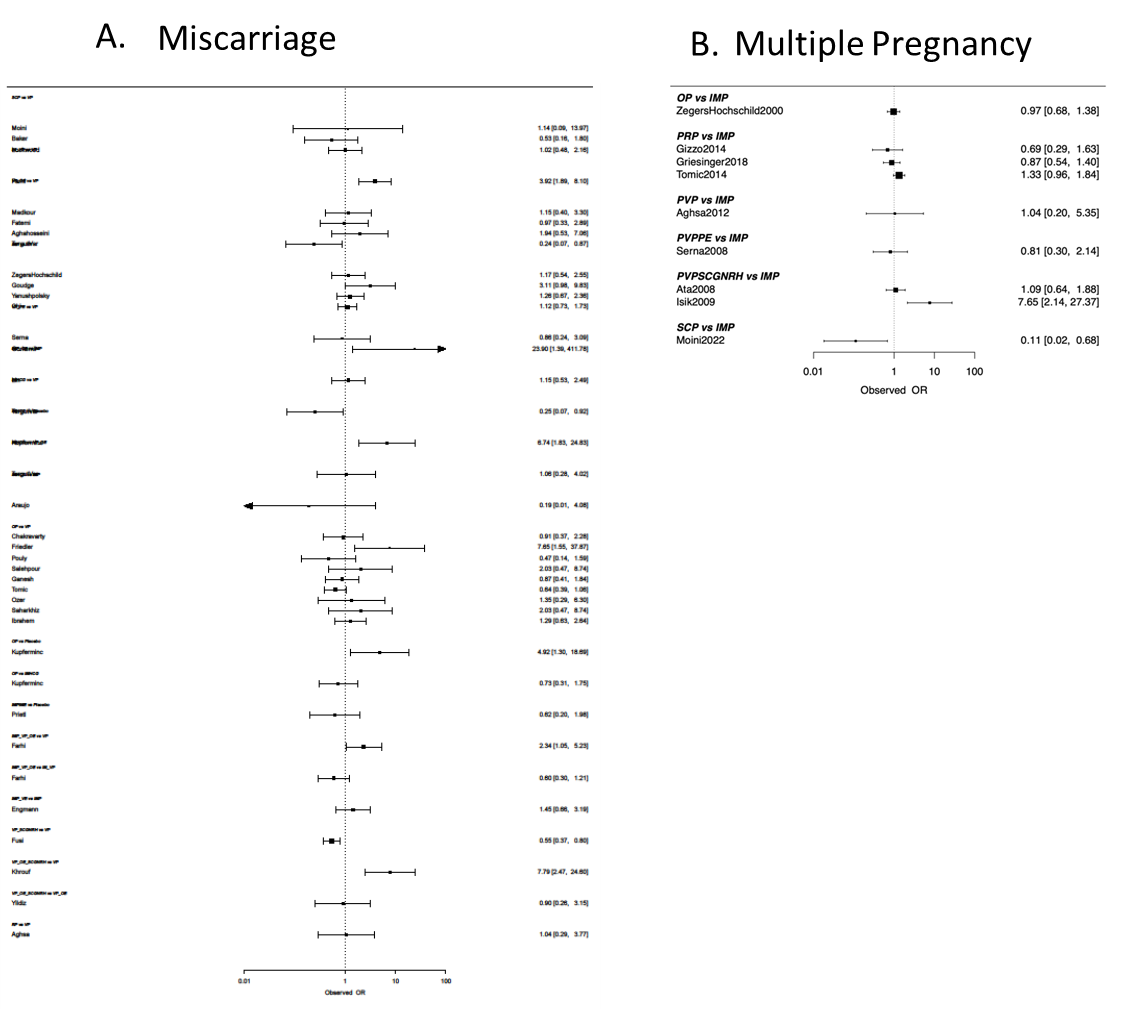


Figure S4. Pairwise comparisons of luteal support protocols measuring miscarriage (A) and multiple pregnancy events outcomes (B). OHSS Pairwise analysis not feasible as multiple non-events (0 out of the total population). Maentel Haensel Odds ratio (95% CI) forest plot generated with MetaInsight freeware package. Abbreviations: placebo (no exposure), SCP (Subcutaneous progesterone), VP (vaginal progesterone), IMP+VP (intramuscular progesterone and vaginal progesterone), VP+OE (vaginal progesterone and oral estradiol), IMP (intramuscular progesterone), VP+PatchE (vaginal progesterone and patch oestrogen), IMP+OE (intramuscular progesterone and oral estradiol), IMHCG (intramuscular hCG), SCP+VP, Intranasal GnRH-a, OP (oral progesterone), IMP+IME (intramuscular progesterone and intramuscular estradiol), IMP+VP+OE (Intramuscular progesterone, vaginal progesterone and oral estradiol), IMP+VE (Intramuscular progesterone and vaginal estradiol), VP+SCGNRH-a [(Vaginal progesterone and subcutaneous GNRH agonist (GNRH-a)], VP+OE+SCGNRH-a (Vaginal progesterone, oral estradiol and subcutaneous GNRH-a), RP (Rectal progesterone), SCHCG (subcutaneous HCG), VP+DHEA (vaginal progesterone and oral DHEA), IMP+VP+SCGNRH-a (Intramuscular progesterone, vaginal progesterone and subcutaneous GNRH-a), OP+VP (oral progesterone and vaginal progesterone.


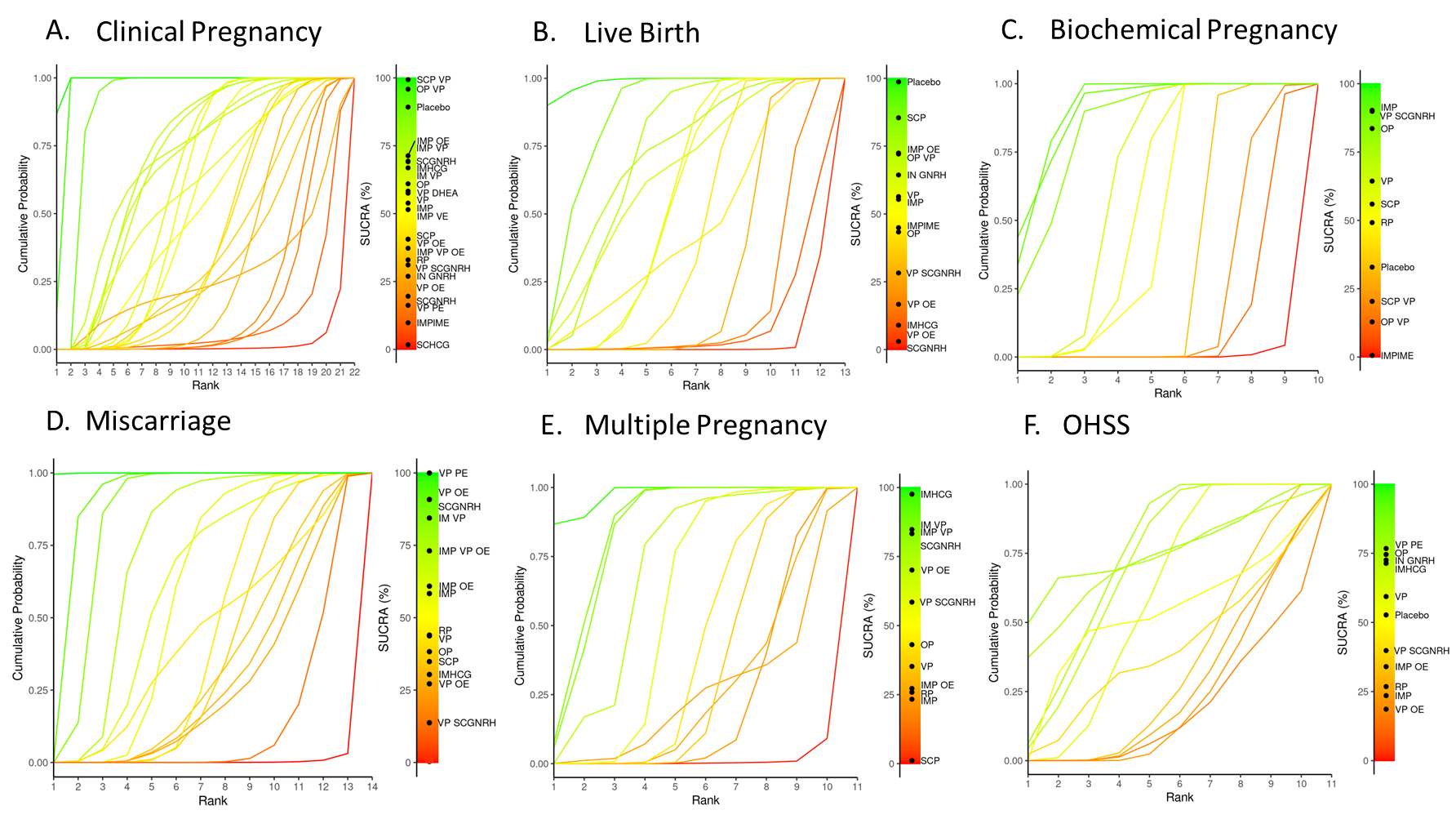


Figure S5. Litmus Rank-O-Gram; Higher SUCRA (Surface Under the Cumulative Ranking Curve) values Clinical pregnancy (A), Live Birth (B), Biochemical Pregnancy (C), Miscarriage (D) and Multiple pregnancy (E), OHSS (F). Cumulative ranking curves nearer the top left indicate better performance, generated with MetaInsight freeware package. Abbreviations: placebo (no exposure), SCP (Subcutaneous progesterone), VP (vaginal progesterone), IMP+VP (intramuscular progesterone and vaginal progesterone), VP+OE (vaginal progesterone and oral estradiol), IMP (intramuscular progesterone), VP+PatchE (vaginal progesterone and patch oestrogen), IMP+OE (intramuscular progesterone and oral estradiol), IMHCG (intramuscular hCG), SCP+VP, Intranasal GnRH-a, OP (oral progesterone), IMP+IME (intramuscular progesterone and intramuscular estradiol), IMP+VP+OE (Intramuscular progesterone, vaginal progesterone and oral estradiol), IMP+VE (Intramuscular progesterone and vaginal estradiol), VP+SCGNRH-a [(Vaginal progesterone and subcutaneous GNRH agonist (GNRH-a)], VP+OE+SCGNRH-a (Vaginal progesterone, oral estradiol and subcutaneous GNRH-a), RP (Rectal progesterone), SCHCG (subcutaneous HCG), VP+DHEA (vaginal progesterone and oral DHEA), IMP+VP+SCGNRH-a (Intramuscular progesterone, vaginal progesterone and subcutaneous GNRH-a), OP+VP (oral progesterone and vaginal progesterone).


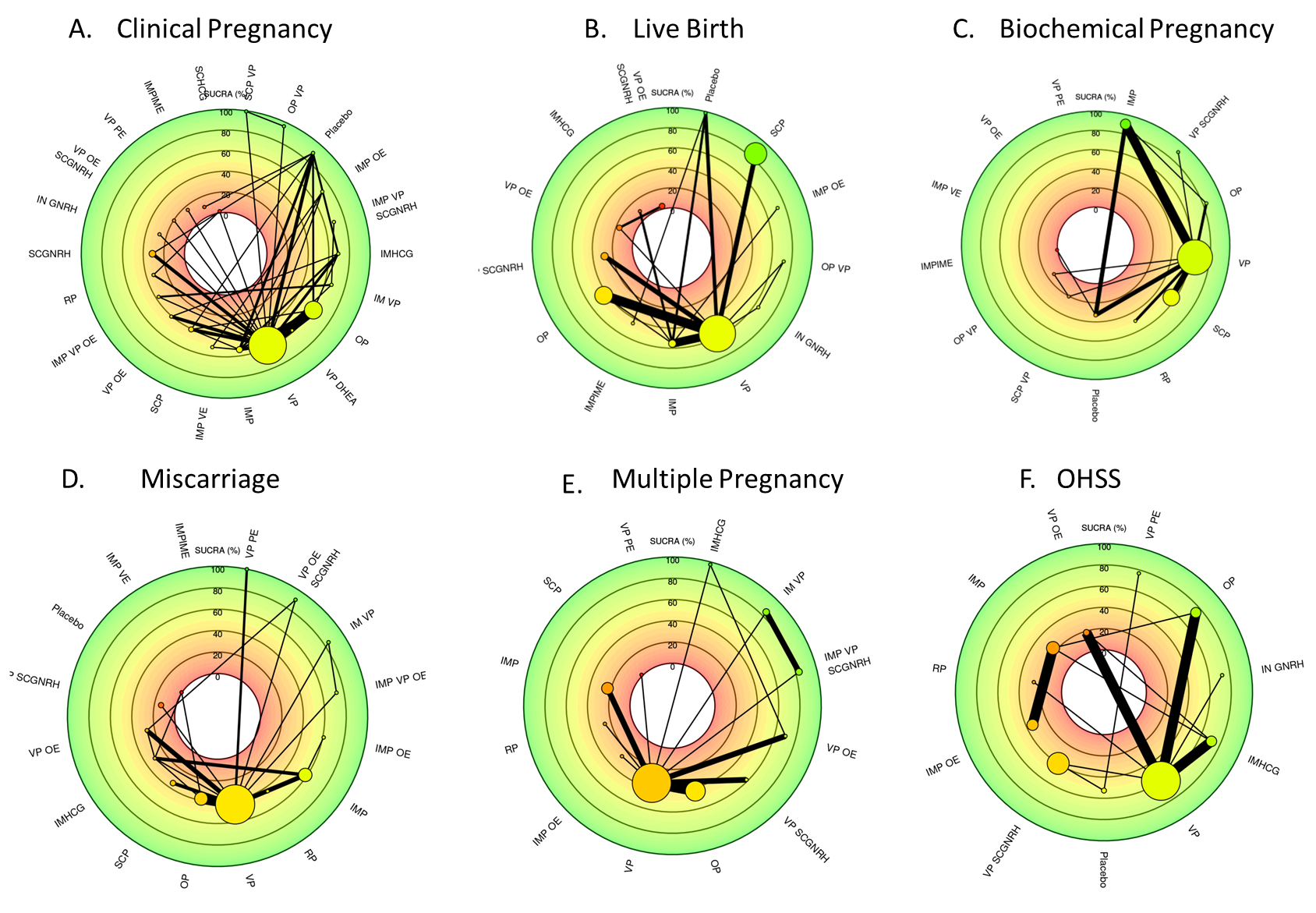
Figure S6. Radial SUCRA plot of NMA included LPS protocols. Clinical pregnancy (A), Live Birth (B), Biochemical Pregnancy (C), Miscarriage (D) and Multiple pregnancy (E), OHSS (F). Higher SUCRA values indicate better treatments; size of nodes represents number of participants and thickness of lines indicate number of trials conducted. Data analysed with MetaInsight freeware package. Abbreviations: placebo (no exposure), SCP (Subcutaneous progesterone), VP (vaginal progesterone), IMP+VP (intramuscular progesterone and vaginal progesterone), VP+OE (vaginal progesterone and oral estradiol), IMP (intramuscular progesterone), VP+PatchE (vaginal progesterone and patch oestrogen), IMP+OE (intramuscular progesterone and oral estradiol), IMHCG (intramuscular hCG), SCP+VP, Intranasal GnRH-a, OP (oral progesterone), IMP+IME (intramuscular progesterone and intramuscular estradiol), IMP+VP+OE (Intramuscular progesterone, vaginal progesterone and oral estradiol), IMP+VE (Intramuscular progesterone and vaginal estradiol), VP+SCGNRH-a [(Vaginal progesterone and subcutaneous GNRH agonist (GNRH-a)], VP+OE+SCGNRH-a (Vaginal progesterone, oral estradiol and subcutaneous GNRH-a), RP (Rectal progesterone), SCHCG (subcutaneous HCG), VP+DHEA (vaginal progesterone and oral DHEA), IMP+VP+SCGNRH-a (Intramuscular progesterone, vaginal progesterone and subcutaneous GNRH-a), OP+VP (oral progesterone and vaginal progesterone).


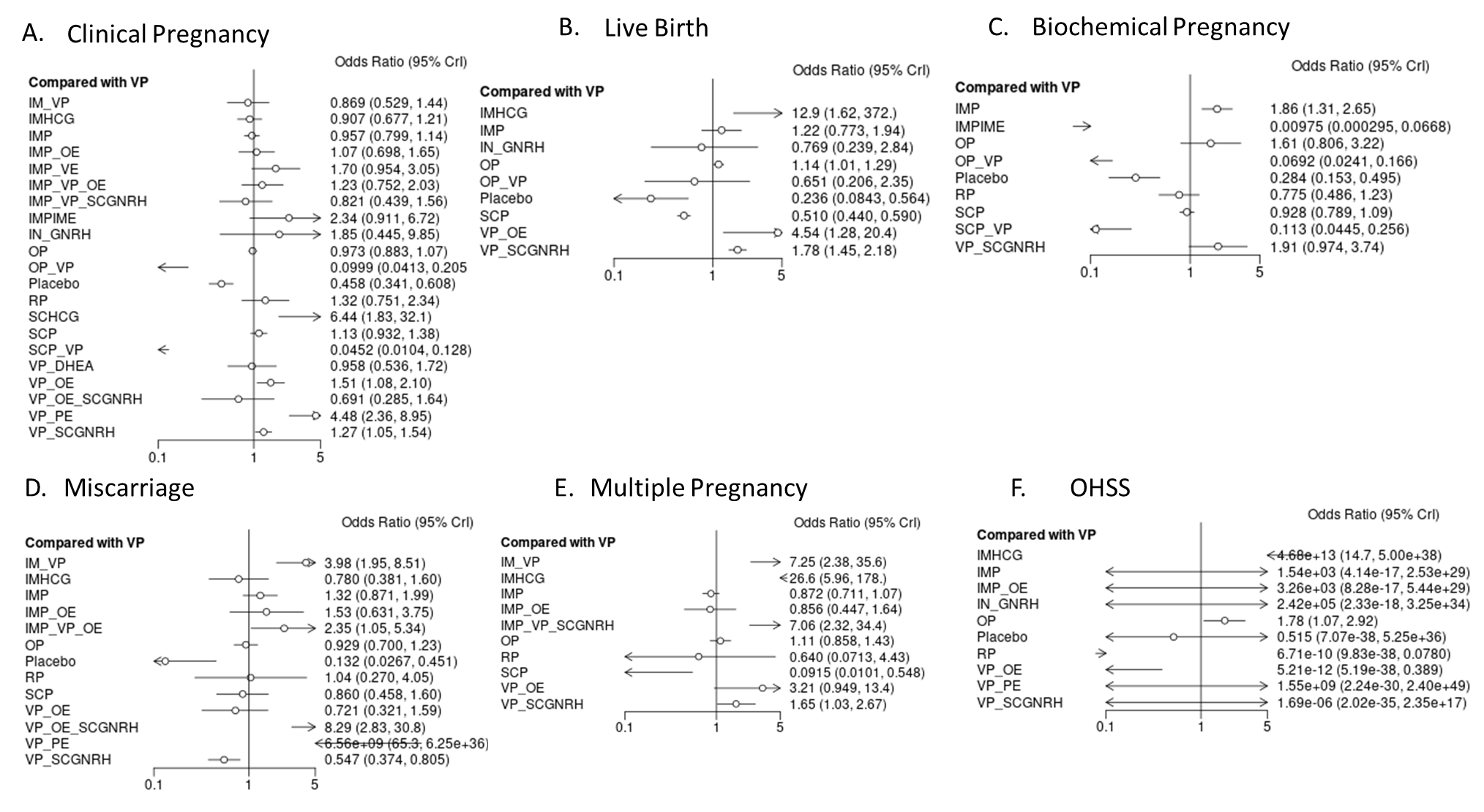
Figure S7. Subgroup analysis of studies with low risk of bias or “some concerns” according to the Cochrane risk-of-bias tool for randomized trials proposed judgement. Luteal support Bayesian fixed effect consistency forest plot (Odds ratio, 95% CI) for Clinical Pregnancy (A) Live Birth (B) Biochemical Pregnancy (C) Miscarriage (D) and Multiple pregnancy ( E), OHSS (F) outcomes. Graph generated by MetaInsight R package. Abbreviations: placebo (no exposure), SCP (Subcutaneous progesterone), VP (vaginal progesterone), IMP+VP (intramuscular progesterone and vaginal progesterone), VP+OE (vaginal progesterone and oral estradiol), IMP (intramuscular progesterone), VP+PatchE (vaginal progesterone and patch oestrogen), IMP+OE (intramuscular progesterone and oral estradiol), IMHCG (intramuscular hCG), SCP+VP, Intranasal GnRH-a, OP (oral progesterone), IMP+IME (intramuscular progesterone and intramuscular estradiol), IMP+VP+OE (Intramuscular progesterone, vaginal progesterone and oral estradiol), IMP+VE (Intramuscular progesterone and vaginal estradiol), VP+SCGNRH-a [(Vaginal progesterone and subcutaneous GNRH agonist (GNRH-a)], VP+OE+SCGNRH-a (Vaginal progesterone, oral estradiol and subcutaneous GNRH-a), RP (Rectal progesterone), SCHCG (subcutaneous HCG), VP+DHEA (vaginal progesterone and oral DHEA), IMP+VP+SCGNRH-a (Intramuscular progesterone, vaginal progesterone and subcutaneous GNRH-a), OP+VP (oral progesterone and vaginal progesterone).


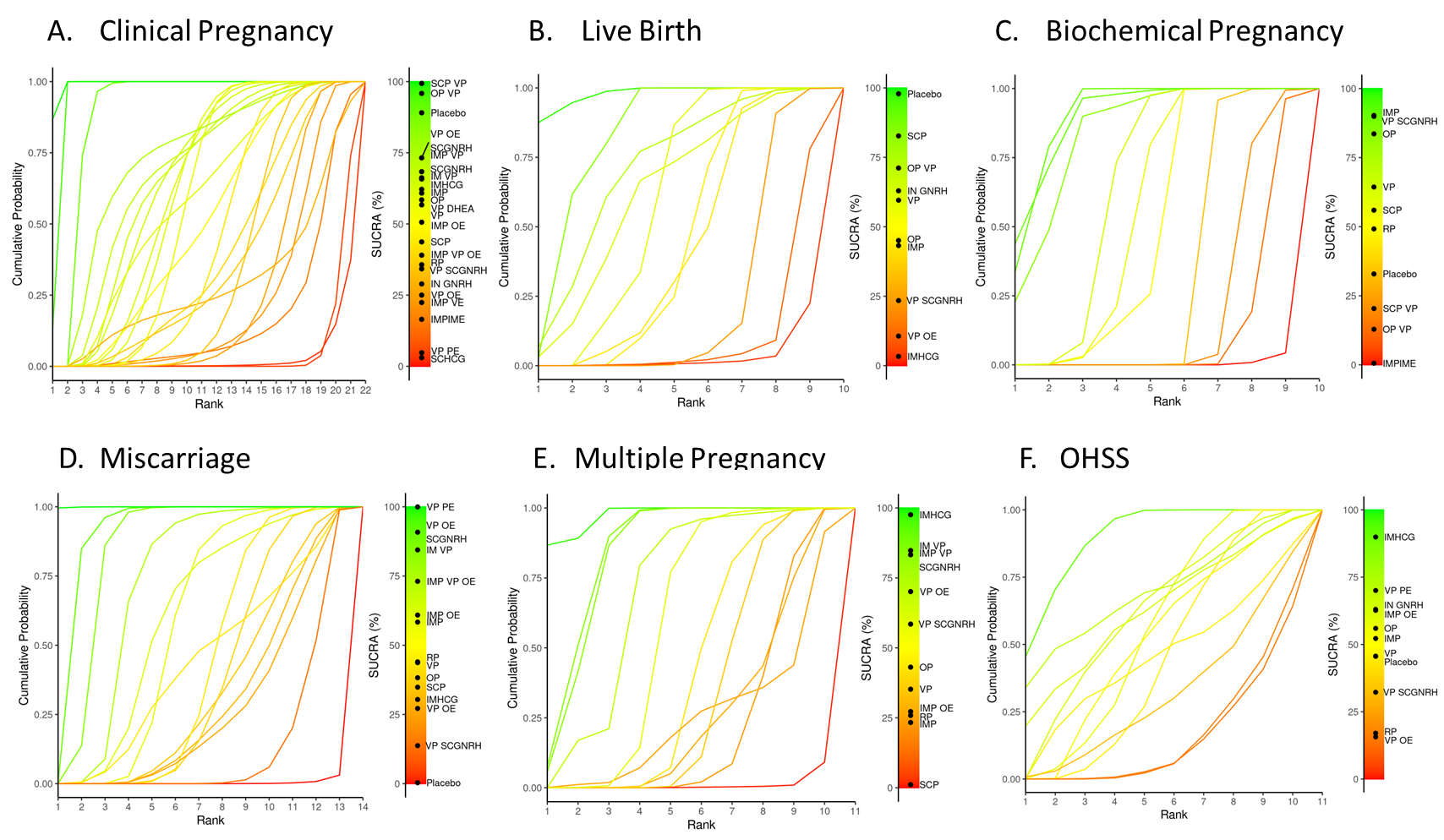
Figure S8. Litmus Rank-O-Gram of subgroup analysis; Higher SUCRA (Surface Under the Cumulative Ranking Curve) values; Clinical pregnancy (A), Live Birth (B), Biochemical Pregnancy (C), Miscarriage (D),Multiple pregnancy (E) and OHSS (F). Cumulative ranking curves nearer the top left indicate better performance, generated with MetaInsight freeware package.


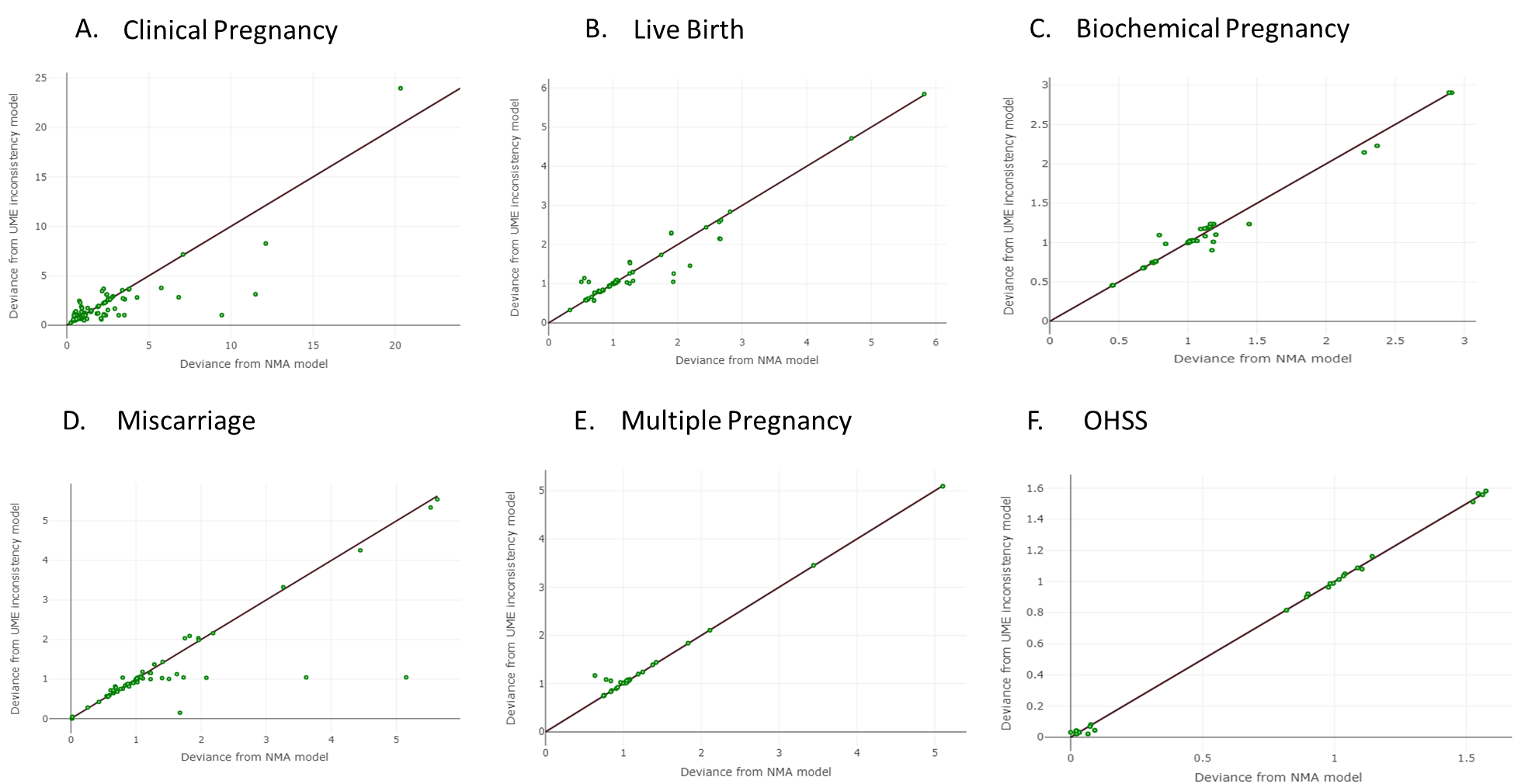


Figure S9. **Residual deviance from NMA model and UME inconsistency model for all studies.** Clinical pregnancy (A), Live Birth (B), Biochemical Pregnancy (C), Miscarriage (D),Multiple pregnancy (E) and OHSS (F). This plot represents each data points' contribution to the residual deviance for the NMA with consistency (horizontal axis) and the unrelated mean effect (ume) inconsistency models (vertical axis) along with the line of equality. The points on the equality line means there is no improvement in model fit when using the inconsistency model, suggesting that there is no evidence of inconsistency. Points above the equality line means they have a smaller residual deviance for the consistency model indicating a better fit in the NMA consistency model and points below the equality line means they have a better fit in the ume inconsistency model.


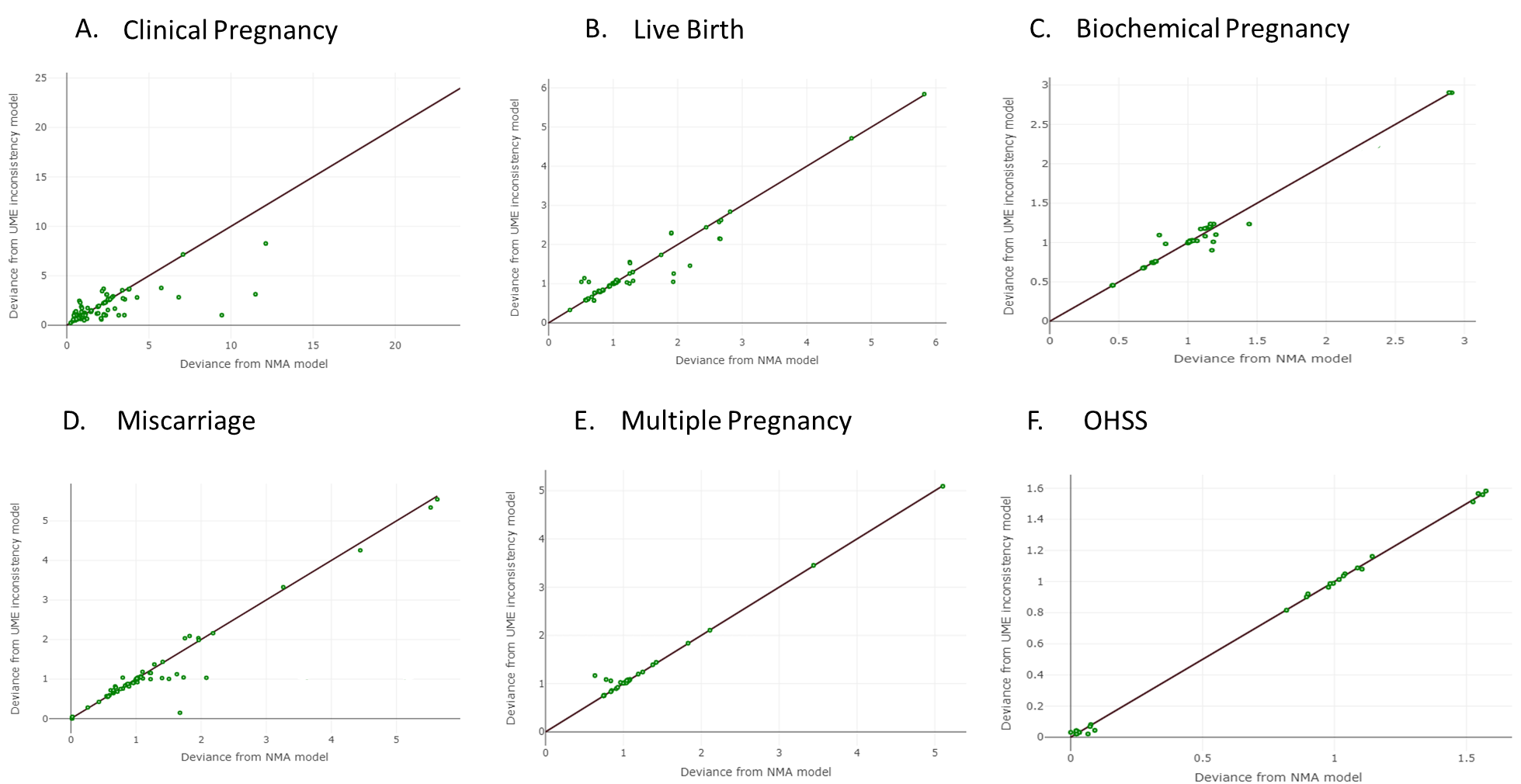


Figure S10. **Residual deviance from subgroup analysis of NMA model and UME inconsistency model for low and moderate RoB studies.** Clinical pregnancy (A), Live Birth (B), Biochemical Pregnancy (C), Miscarriage (D), Multiple pregnancy (E) and OHSS (F). This plot represents each data points' contribution to the residual deviance for the NMA with consistency (horizontal axis) and the unrelated mean effect (ume) inconsistency models (vertical axis) along with the line of equality. The points on the equality line means there is no improvement in model fit when using the inconsistency model, suggesting that there is no evidence of inconsistency. Points above the equality line means they have a smaller residual deviance for the consistency model indicating a better fit in the NMA consistency model and points below the equality line means they have a better fit in the ume inconsistency model.

Tables S1-11

| **Author et al., year** | **Exclusion reason** | **Reference** |
| --- | --- | --- |
| Feinberg et al., 2013 | Quasi-RCT Observational | Feinberg, E. C., Beltsos, A. N., Nicolaou, E., Marut, E. L., & Uhler, M. L. (2013). Endometrin as luteal phase support in assisted reproduction. Fertility and sterility, 99(1), 174-178. |
| Ho et al., 2008 | Quasi-RCT Observational | Ho, C. H., Chen, S. U., Peng, F. S., Chang, C. Y., & Yang, Y. S. (2008). Luteal support for IVF/ICSI cycles with Crinone 8%(90 mg) twice daily results in higher pregnancy rates than with intramuscular progesterone. Journal of the Chinese Medical Association, 71(8), 386-391. |
| Gari et al., 2022 | Quasi-RCT Observational | Gari, S., & Al-Jaroudi, D. (2022). Adding Weekly Intramuscular Progesterone to a Twice Daily Vaginal Progesterone Capsule for Luteal Phase Support in IVF/ICSI Cycles Results in Similar Live Birth Rates. JBRA Assisted Reproduction, 26(1), 33. |
| Kao et al., 2022 | Quasi-RCT Drop out rate 47.6%; treated population demographics not stated | Kao, T. C., Tu, Y. A., Yang, P. K., Huang, C. C., Yang, J. H., Chen, S. U., & Chao, K. H. (2022). Clinical use of aqueous subcutaneous progesterone compared with vaginal progesterone as luteal support in in vitro fertilization: A randomized controlled study in Taiwan. Taiwanese Journal of Obstetrics and Gynecology, 61(5), 863-867. |
| Qublan et al., 2008 | both groups received progesterone pessaries in unknown doses | Qublan, H., Amarin, Z., Al-Quda, M., Diab, F., Nawasreh, M., Malkawi, S., & Balawneh, M. (2008). Luteal phase support with GnRH-a improves implantation and pregnancy rates in IVF cycles with endometrium of≤ 7 mm on day of egg retrieval. Human Fertility, 11(1), 43-47. |
| Rodriguez-Pezino et al., 2004 | Pdf unretrievable | Rodriguez‐Pezino J, Saucedo‐de la Llata E, Batiza‐Resendiz V, Galache‐Vega P, Santos‐Haliscak R, Hernandez‐Ayup S, et al. Vaginal progesterone in assisted reproduction. Human Reproduction. Berlin, Germany, 2004; Vol. 19 Suppl 1:i51. |
| Mui Lam et al., 2008 | Adressing short term Luteal support (between OPU and ET) | Mui Lam, P. O., Chun Cheung, M., Ping Cheung, L., Ingrid Lok, H., & John Haines, C. (2008). Effects of early luteal-phase vaginal progesterone supplementation on the outcome of in vitro fertilization and embryo transfer. Gynecological Endocrinology, 24(12), 674-680. |
| Zafardoust, et al. | LPS protocol in control group not clarified | Zafardoust S, Jeddi-Tehrani M, Akhondi MM, Sadeghi MR, Kamali K, Mokhtar S, Badehnoosh B, Arjmand-Teymouri F, Fatemi F, Mohammadzadeh A. Effect of administration of single dose GnRH agonist in luteal phase on outcome of ICSI-ET cycles in women with previous history of IVF/ICSI failure: a randomized controlled trial. Journal of Reproduction & Infertility. 2015 Apr;16(2):96. |
| Qu, et al.,2020 | Quasi-RCT | Qu D, Li Y. Multiple-Dose Versus Single-Dose Gonadotropin-Releasing Hormone Agonist After First In Vitro Fertilization Failure Associated With Luteal Phase Deficiency: A Randomized Controlled Trial. J Int Med Res (2020) 48:300060520926026. doi: 10.1177/0300060520926026 |
| Chavez et al., 2004 | Article in Spanish | Chavez FCP, Delgadillo JCB, Rueda SSO, Villa GB, Acosta SV, Solis VS, Caballero MO, Gavino FG. Estrogen role in the luteal phase support in in vitro fertilization with embryo transfer cycles. Ginecol Obstet Mex. 2004;72:645–655. |
| Samsami et al., 2016 | Pdf unretrievable | Samsami A, Zarei A, Shahrivar S. Effects of estradiol injection on outcome of in-vitro fertilization: a randomized, double-blind, placebo controlled trial. Clin Exp Obstet Gynecol. 2016;43(6):875–879. |

Table S1. Excluded studies and justification.

| Clinical Pregnancy |  |  |  | Live Birth |  |  |  | Biochemical Pregnancy | |  |  |
| --- | --- | --- | --- | --- | --- | --- | --- | --- | --- | --- | --- |
|  | comparison | p.value | CrI |  | comparison | p.value | CrI |  | comparison | p.value | CrI |
| 1 | d.IMHCG.IMP | 0.28915 | NA | 1 | d.IMP.OP | 0.751625 | NA | 1 | d.IMP.OP | 0.2537 | NA |
| 2 | -> direct | NA | -0.22 (-0.93, 0.47) | 2 | -> direct | NA | 0.38 (-1.3, 2.2) | 2 | -> direct | NA | -0.52 (-2.0, 0.88) |
| 3 | -> indirect | NA | 0.19 (-0.13, 0.51) | 3 | -> indirect | NA | 0.10 (-0.12, 0.33) | 3 | -> indirect | NA | 0.42 (-0.40, 1.3) |
| 4 | -> network | NA | 0.15 (-0.14, 0.43) | 4 | -> network | NA | 0.11 (-0.12, 0.34) | 4 | -> network | NA | 0.18 (-0.52, 0.88) |
| 5 | d.IMHCG.OP | 0.565025 | NA | 5 | d.IMP.Placebo | 0.03095 | NA | 5 | d.IMP.VP | 0.256825 | NA |
| 6 | -> direct | NA | 0.33 (-0.54, 1.2) | 6 | -> direct | NA | -2.8 (-4.8, -1.5) | 6 | -> direct | NA | -0.17 (-0.40, 0.053) |
| 7 | -> indirect | NA | 0.060 (-0.23, 0.35) | 7 | -> indirect | NA | -0.59 (-2.1, 0.77) | 7 | -> indirect | NA | -1.1 (-2.8, 0.51) |
| 8 | -> network | NA | 0.098 (-0.17, 0.37) | 8 | -> network | NA | -1.6 (-2.6, -0.69) | 8 | -> network | NA | -0.19 (-0.42, 0.029) |
| 9 | d.IMHCG.Placebo | 0.00025 | NA | 9 | d.IMP.VP | 0.741675 | NA | 9 | d.OP.VP | 0.2583 | NA |
| 10 | -> direct | NA | 0.019 (-0.39, 0.43) | 10 | -> direct | NA | -0.076 (-0.28, 0.13) | 10 | -> direct | NA | -0.59 (-1.4, 0.19) |
| 11 | -> indirect | NA | -1.1 (-1.6, -0.65) | 11 | -> indirect | NA | 0.21 (-1.5, 2.0) | 11 | -> indirect | NA | 0.34 (-1.1, 1.8) |
| 12 | -> network | NA | -0.51 (-0.82, -0.20) | 12 | -> network | NA | -0.069 (-0.27, 0.13) | 12 | -> network | NA | -0.37 (-1.1, 0.31) |
| 13 | d.IMHCG.VP | 0.149975 | NA | 13 | d.IMP.VP_SCGNRH | 0.057 | NA |  |  |  |  |
| 14 | -> direct | NA | -0.12 (-0.47, 0.24) | 14 | -> direct | NA | 1.1 (0.42, 1.9) | |  |  |  |
| 15 | -> indirect | NA | 0.29 (-0.13, 0.72) | 15 | -> indirect | NA | 0.36 (0.053, 0.68) | |  |  |  |
| 16 | -> network | NA | 0.12 (-0.14, 0.38) | 16 | -> network | NA | 0.50 (0.23, 0.77) | |  |  |  |
| 17 | d.IMHCG.VP_OE | 0.41245 | NA | 17 | d.OP.VP | 0.741375 | NA |  |  |  |  |
| 18 | -> direct | NA | 0.075 (-0.51, 0.65) | 18 | -> direct | NA | -0.18 (-0.29, -0.064) | |  |  |  |
| 19 | -> indirect | NA | 0.37 (-0.042, 0.78) | 19 | -> indirect | NA | -0.47 (-2.3, 1.3) | |  |  |  |
| 20 | -> network | NA | 0.27 (-0.069, 0.61) | 20 | -> network | NA | -0.18 (-0.29, -0.067) | |  |  |  |
| 21 | d.IMP.IMP_OE | 0.98565 | NA |  |  |  |  |  |  |  |  |
| 22 | -> direct | NA | -0.12 (-0.45, 0.22) |  |  |  |  |  |  |  |  |
| 23 | -> indirect | NA | -0.11 (-0.71, 0.49) |  |  |  |  |  |  |  |  |
| 24 | -> network | NA | -0.18 (-0.46, 0.11) |  |  |  |  |  |  |  |  |
| 25 | d.IMP.OP | 0.8194 | NA |  |  |  |  |  |  |  |  |
| 26 | -> direct | NA | -0.095 (-0.52, 0.33) |  |  |  |  |  |  |  |  |
| 27 | -> indirect | NA | -0.041 (-0.23, 0.15) |  |  |  |  |  |  |  |  |
| 28 | -> network | NA | -0.048 (-0.22, 0.13) |  |  |  |  |  |  |  |  |
| 29 | d.IMP.Placebo | 0.387225 | NA |  |  |  |  |  |  |  |  |
| 30 | -> direct | NA | -0.45 (-1.0, 0.10) |  |  |  |  |  |  |  |  |
| 31 | -> indirect | NA | -0.74 (-1.1, -0.42) |  |  |  |  |  |  |  |  |
| 32 | -> network | NA | -0.66 (-0.94, -0.38) |  |  |  |  |  |  |  |  |
| 33 | d.IMP.SCP | 0.960725 | NA |  |  |  |  |  |  |  |  |
| 34 | -> direct | NA | 0.14 (-0.90, 1.2) |  |  |  |  |  |  |  |  |
| 35 | -> indirect | NA | 0.11 (-0.13, 0.36) |  |  |  |  |  |  |  |  |
| 36 | -> network | NA | 0.11 (-0.13, 0.35) |  |  |  |  |  |  |  |  |
| 37 | d.IMP.VP | 0.63885 | NA |  |  |  |  |  |  |  |  |
| 38 | -> direct | NA | -0.043 (-0.21, 0.12) |  |  |  |  |  |  |  |  |
| 39 | -> indirect | NA | 0.065 (-0.35, 0.48) |  |  |  |  |  |  |  |  |
| 40 | -> network | NA | -0.027 (-0.18, 0.12) |  |  |  |  |  |  |  |  |
| 41 | d.IMP_OE.IMP_VE | 0.138175 | NA |  |  |  |  |  |  |  |  |
| 42 | -> direct | NA | 0.37 (-0.23, 0.97) |  |  |  |  |  |  |  |  |
| 43 | -> indirect | NA | -0.33 (-1.0, 0.38) |  |  |  |  |  |  |  |  |
| 44 | -> network | NA | 0.20 (-0.25, 0.65) |  |  |  |  |  |  |  |  |
| 45 | d.IMP_OE.VP | 0.80335 | NA |  |  |  |  |  |  |  |  |
| 46 | -> direct | NA | 0.090 (-0.48, 0.67) |  |  |  |  |  |  |  |  |
| 47 | -> indirect | NA | 0.18 (-0.18, 0.53) |  |  |  |  |  |  |  |  |
| 48 | -> network | NA | 0.15 (-0.16, 0.45) |  |  |  |  |  |  |  |  |
| 49 | d.OP.Placebo | 0.9209 | NA |  |  |  |  |  |  |  |  |
| 50 | -> direct | NA | -0.29 (-0.80, 0.22) |  |  |  |  |  |  |  |  |
| 51 | -> indirect | NA | -0.32 (-0.68, 0.015) |  |  |  |  |  |  |  |  |
| 52 | -> network | NA | -0.61 (-0.88, -0.35) |  |  |  |  |  |  |  |  |
| 53 | d.OP.SCP | 0.000025 | NA |  |  |  |  |  |  |  |  |
| 54 | -> direct | NA | 1.1 (0.67, 1.6) |  |  |  |  |  |  |  |  |
| 55 | -> indirect | NA | -0.022 (-0.32, 0.28) |  |  |  |  |  |  |  |  |
| 56 | -> network | NA | 0.16 (-0.049, 0.37) |  |  |  |  |  |  |  |  |
| 57 | d.OP.VP | 0.734575 | NA |  |  |  |  |  |  |  |  |
| 58 | -> direct | NA | 0.024 (-0.072, 0.12) |  |  |  |  |  |  |  |  |
| 59 | -> indirect | NA | 0.15 (-0.54, 0.85) |  |  |  |  |  |  |  |  |
| 60 | -> network | NA | 0.021 (-0.072, 0.11) |  |  |  |  |  |  |  |  |
| 61 | d.Placebo.SCP | 0.000075 | NA |  |  |  |  |  |  |  |  |
| 62 | -> direct | NA | 1.5 (1.0, 2.1) |  |  |  |  |  |  |  |  |
| 63 | -> indirect | NA | 0.27 (-0.15, 0.69) |  |  |  |  |  |  |  |  |
| 64 | -> network | NA | 0.77 (0.48, 1.1) |  |  |  |  |  |  |  |  |
| 65 | d.Placebo.VP | 0.001825 | NA |  |  |  |  |  |  |  |  |
| 66 | -> direct | NA | 1.1 (0.73, 1.5) |  |  |  |  |  |  |  |  |
| 67 | -> indirect | NA | 0.26 (-0.11, 0.63) |  |  |  |  |  |  |  |  |
| 68 | -> network | NA | 0.63 (0.38, 0.89) |  |  |  |  |  |  |  |  |
| 69 | d.Placebo.VP_PE | 0.00945 | NA |  |  |  |  |  |  |  |  |
| 70 | -> direct | NA | -0.064 (-1.1, 0.97) |  |  |  |  |  |  |  |  |
| 71 | -> indirect | NA | 1.5 (0.96, 2.0) |  |  |  |  |  |  |  |  |
| 72 | -> network | NA | 1.2 (0.72, 1.6) |  |  |  |  |  |  |  |  |
| 73 | d.Placebo.VP_SCGNRH | 0.47395 | NA |  |  |  |  |  |  |  |  |
| 74 | -> direct | NA | 1.2 (0.35, 2.0) |  |  |  |  |  |  |  |  |
| 75 | -> indirect | NA | 0.83 (0.50, 1.2) |  |  |  |  |  |  |  |  |
| 76 | -> network | NA | 0.88 (0.57, 1.2) |  |  |  |  |  |  |  |  |
| 77 | d.SCP.VP | 0.6312 | NA |  |  |  |  |  |  |  |  |
| 78 | -> direct | NA | 0.074 (-0.13, 0.28) |  |  |  |  |  |  |  |  |
| 79 | -> indirect | NA | -0.19 (-1.3, 0.87) |  |  |  |  |  |  |  |  |
| 80 | -> network | NA | -0.14 (-0.33, 0.055) |  |  |  |  |  |  |  |  |
| 81 | d.VP.VP_OE | 0.02715 | NA |  |  |  |  |  |  |  |  |
| 82 | -> direct | NA | 0.30 (0.035, 0.57) |  |  |  |  |  |  |  |  |
| 83 | -> indirect | NA | -0.74 (-1.7, 0.15) |  |  |  |  |  |  |  |  |
| 84 | -> network | NA | 0.15 (-0.10, 0.40) |  |  |  |  |  |  |  |  |
| 85 | d.VP.VP_OE_SCGNRH | 0.041075 | NA |  |  |  |  |  |  |  |  |
| 86 | -> direct | NA | -0.37 (-1.2, 0.49) |  |  |  |  |  |  |  |  |
| 87 | -> indirect | NA | 0.61 (0.23, 0.99) |  |  |  |  |  |  |  |  |
| 88 | -> network | NA | 0.44 (0.10, 0.79) |  |  |  |  |  |  |  |  |
| 89 | d.VP.VP_PE | 0.0105 | NA |  |  |  |  |  |  |  |  |
| 90 | -> direct | NA | 0.76 (0.32, 1.2) |  |  |  |  |  |  |  |  |
| 91 | -> indirect | NA | -0.77 (-1.8, 0.31) |  |  |  |  |  |  |  |  |
| 92 | -> network | NA | 0.55 (0.14, 0.95) |  |  |  |  |  |  |  |  |
| 93 | d.VP.VP_SCGNRH | 0.49425 | NA |  |  |  |  |  |  |  |  |
| 94 | -> direct | NA | 0.23 (0.031, 0.43) |  |  |  |  |  |  |  |  |
| 95 | -> indirect | NA | 0.55 (-0.31, 1.5) |  |  |  |  |  |  |  |  |
| 96 | -> network | NA | 0.24 (0.055, 0.43) |  |  |  |  |  |  |  |  |
| 97 | d.VP_OE.VP_OE_SCGNRH | 0.042 | NA |  |  |  |  |  |  |  |  |
| 98 | -> direct | NA | 0.38 (0.10, 0.67) |  |  |  |  |  |  |  |  |
| 99 | -> indirect | NA | -0.59 (-1.5, 0.30) |  |  |  |  |  |  |  |  |
| 100 | -> network | NA | 0.29 (0.027, 0.56) |  |  |  |  |  |  |  |  |

Table S2. Bayesian node splitting, consistency assessment of direct and indirect OR for primary outcomes (Clinical Pregnancy, Live Birth, Biochemical pregnancy). Abbreviations: placebo (no exposure), SCP (Subcutaneous progesterone), VP (vaginal progesterone), IMP+VP (intramuscular progesterone and vaginal progesterone), VP+OE (vaginal progesterone and oral estradiol), IMP (intramuscular progesterone), VP+PatchE (vaginal progesterone and patch oestrogen), IMP+OE (intramuscular progesterone and oral estradiol), IMHCG (intramuscular hCG), SCP+VP, Intranasal GnRH-a, OP (oral progesterone), IMP+IME (intramuscular progesterone and intramuscular estradiol), IMP+VP+OE (Intramuscular progesterone, vaginal progesterone and oral estradiol), IMP+VE (Intramuscular progesterone and vaginal estradiol), VP+SCGNRH-a [(Vaginal progesterone and subcutaneous GNRH agonist (GNRH-a)], VP+OE+SCGNRH-a (Vaginal progesterone, oral estradiol and subcutaneous GNRH-a), RP (Rectal progesterone), SCHCG (subcutaneous HCG), VP+DHEA (vaginal progesterone and oral DHEA), IMP+VP+SCGNRH-a (Intramuscular progesterone, vaginal progesterone and subcutaneous GNRH-a), OP+VP (oral progesterone and vaginal progesterone).

| Miscarriage | comparison | p.value | CrI | Multiple Pregnancies | |  | OHSS |  |  |  |
| --- | --- | --- | --- | --- | --- | --- | --- | --- | --- | --- |
| 1 | d.IMHCG.IMP | 0.03025 | NA | Node Splitting not feasible | | |  | comparison | p.value | CrI |
| 2 | -> direct | NA | 12. (0.78, 44.) | |  |  | 1 | d.IMHCG.IMP | 0.19305 | NA |
| 3 | -> indirect | NA | 0.25 (-0.52, 1.1) | |  |  | 2 | -> direct | NA | -27. (-87., -3.1) |
| 4 | -> network | NA | 0.39 (-0.39, 1.2) | |  |  | 3 | -> indirect | NA | 13. (-41., 79.) |
| 5 | d.IMHCG.OP | 0.1501 | NA |  |  |  | 4 | -> network | NA | -16. (-53., -2.2) |
| 6 | -> direct | NA | -0.33 (-1.2, 0.55) | |  |  | 5 | d.IMHCG.VP | 0.193375 | NA |
| 7 | -> indirect | NA | 0.74 (-0.40, 2.0) | |  |  | 6 | -> direct | NA | -0.49 (-1.3, 0.29) |
| 8 | -> network | NA | 0.074 (-0.62, 0.78) | |  |  | 7 | -> indirect | NA | -44. (-1.3e+02, 20.) |
| 9 | d.IMHCG.VP | 0.0586 | NA |  |  |  | 8 | -> network | NA | -0.50 (-1.3, 0.27) |
| 10 | -> direct | NA | 1.4 (0.11, 2.8) | |  |  | 9 | d.IMHCG.VP_OE | 0.9935 | NA |
| 11 | -> indirect | NA | -0.096 (-0.99, 0.77) | |  |  | 10 | -> direct | NA | -26. (-85., -1.9) |
| 12 | -> network | NA | 0.14 (-0.58, 0.86) | |  |  | 11 | -> indirect | NA | -26. (-86., -1.2) |
| 13 | d.IMHCG.VP_OE | 0.636225 | NA |  |  |  | 12 | -> network | NA | -27. (-86., -2.4) |
| 14 | -> direct | NA | -0.070 (-1.4, 1.3) | |  |  | 13 | d.IMP.OP | 0.193275 | NA |
| 15 | -> indirect | NA | 0.34 (-0.72, 1.4) | |  |  | 14 | -> direct | NA | -13. (-80., 40.) |
| 16 | -> network | NA | 0.29 (-0.50, 1.1) | |  |  | 15 | -> indirect | NA | 27. (3.3, 86.) |
| 17 | d.IMP.VP | 0.026925 | NA |  |  |  | 16 | -> network | NA | 16. (2.3, 53.) |
| 18 | -> direct | NA | -0.22 (-0.54, 0.087) | |  |  | 17 | d.OP.VP | 0.1886 | NA |
| 19 | -> indirect | NA | -11. (-40., -0.78) | |  |  | 18 | -> direct | NA | -0.63 (-1.1, -0.14) |
| 20 | -> network | NA | -0.25 (-0.56, 0.068) | |  |  | 19 | -> indirect | NA | 43. (-21., 1.3e+02) |
| 21 | d.OP.VP | 0.163175 | NA |  |  |  | 20 | -> network | NA | -0.63 (-1.1, -0.14) |
| 22 | -> direct | NA | 0.018 (-0.27, 0.31) | |  |  |  |  |  |  |
| 23 | -> indirect | NA | 1.1 (-0.36, 2.6) | |  |  |  |  |  |  |
| 24 | -> network | NA | 0.063 (-0.22, 0.35) | |  |  |  |  |  |  |
| 25 | d.VP.VP_OE | 0.015225 | NA |  |  |  |  |  |  |  |
| 26 | -> direct | NA | -0.12 (-0.71, 0.45) | |  |  |  |  |  |  |
| 27 | -> indirect | NA | 2.2 (0.42, 4.0) | |  |  |  |  |  |  |
| 28 | -> network | NA | 0.15 (-0.40, 0.71) | |  |  |  |  |  |  |
| 29 | d.VP.VP_OE_SCGNRH | 0.019925 | NA |  |  |  |  |  |  |  |
| 30 | -> direct | NA | 2.1 (1.1, 3.5) |  |  |  |  |  |  |  |
| 31 | -> indirect | NA | -0.10 (-1.5, 1.4) | |  |  |  |  |  |  |
| 32 | -> network | NA | 1.4 (0.52, 2.3) | |  |  |  |  |  |  |
| 33 | d.VP_OE.VP_OE_SCGNRH | 0.0177 | NA |  |  |  |  |  |  |  |
| 34 | -> direct | NA | -0.069 (-1.3, 1.3) | |  |  |  |  |  |  |
| 35 | -> indirect | NA | 2.2 (0.94, 3.6) | |  |  |  |  |  |  |
| 36 | -> network | NA | 1.2 (0.30, 2.3) | |  |  |  |  |  |  |

Table S3. Bayesian node splitting, consistency assessment of direct and indirect OR for primary outcomes (Miscarriage, Multiple Pregnancy, OHSS). Abbreviations: placebo (no exposure), SCP (Subcutaneous progesterone), VP (vaginal progesterone), IMP+VP (intramuscular progesterone and vaginal progesterone), VP+OE (vaginal progesterone and oral estradiol), IMP (intramuscular progesterone), VP+PatchE (vaginal progesterone and patch oestrogen), IMP+OE (intramuscular progesterone and oral estradiol), IMHCG (intramuscular hCG), SCP+VP, Intranasal GnRH-a, OP (oral progesterone), IMP+IME (intramuscular progesterone and intramuscular estradiol), IMP+VP+OE (Intramuscular progesterone, vaginal progesterone and oral estradiol), IMP+VE (Intramuscular progesterone and vaginal estradiol), VP+SCGNRH-a [(Vaginal progesterone and subcutaneous GNRH agonist (GNRH-a)], VP+OE+SCGNRH-a (Vaginal progesterone, oral estradiol and subcutaneous GNRH-a), RP (Rectal progesterone), SCHCG (subcutaneous HCG), VP+DHEA (vaginal progesterone and oral DHEA), IMP+VP+SCGNRH-a (Intramuscular progesterone, vaginal progesterone and subcutaneous GNRH-a), OP+VP (oral progesterone and vaginal progesterone).


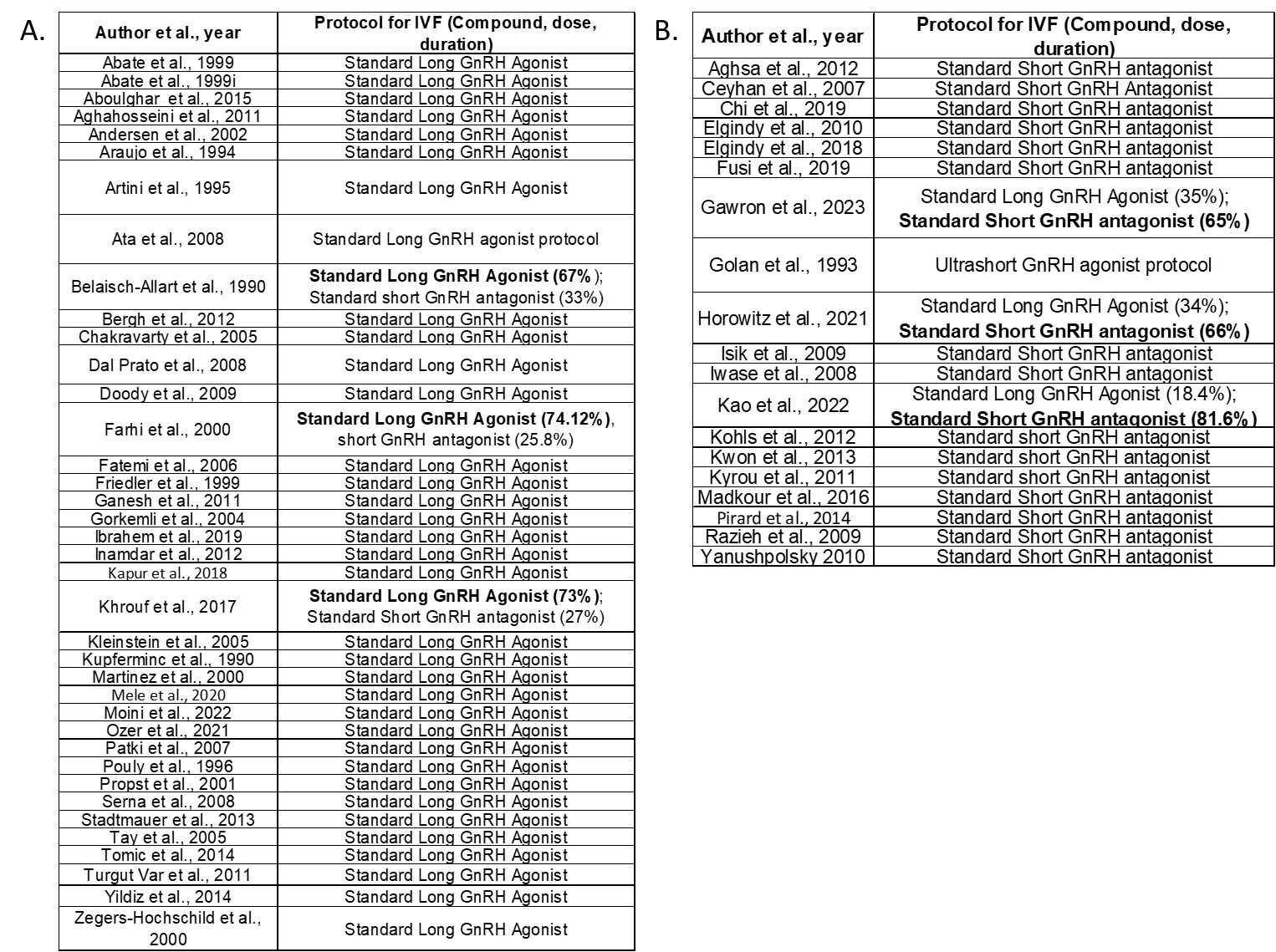


Table S4. Studies included in subgrouping based on stimulation protocol, Standard long GnRH (agonist) Protocol (A) and Standard short (antagonist) GnRH Protocol. If mixed populations were included in the original publication, a cut-off of ≥65% was employed to categorise studies according to subgroup. Studies that did not state how many patients were treated per stimulation protocol were excluded from the subgroup.


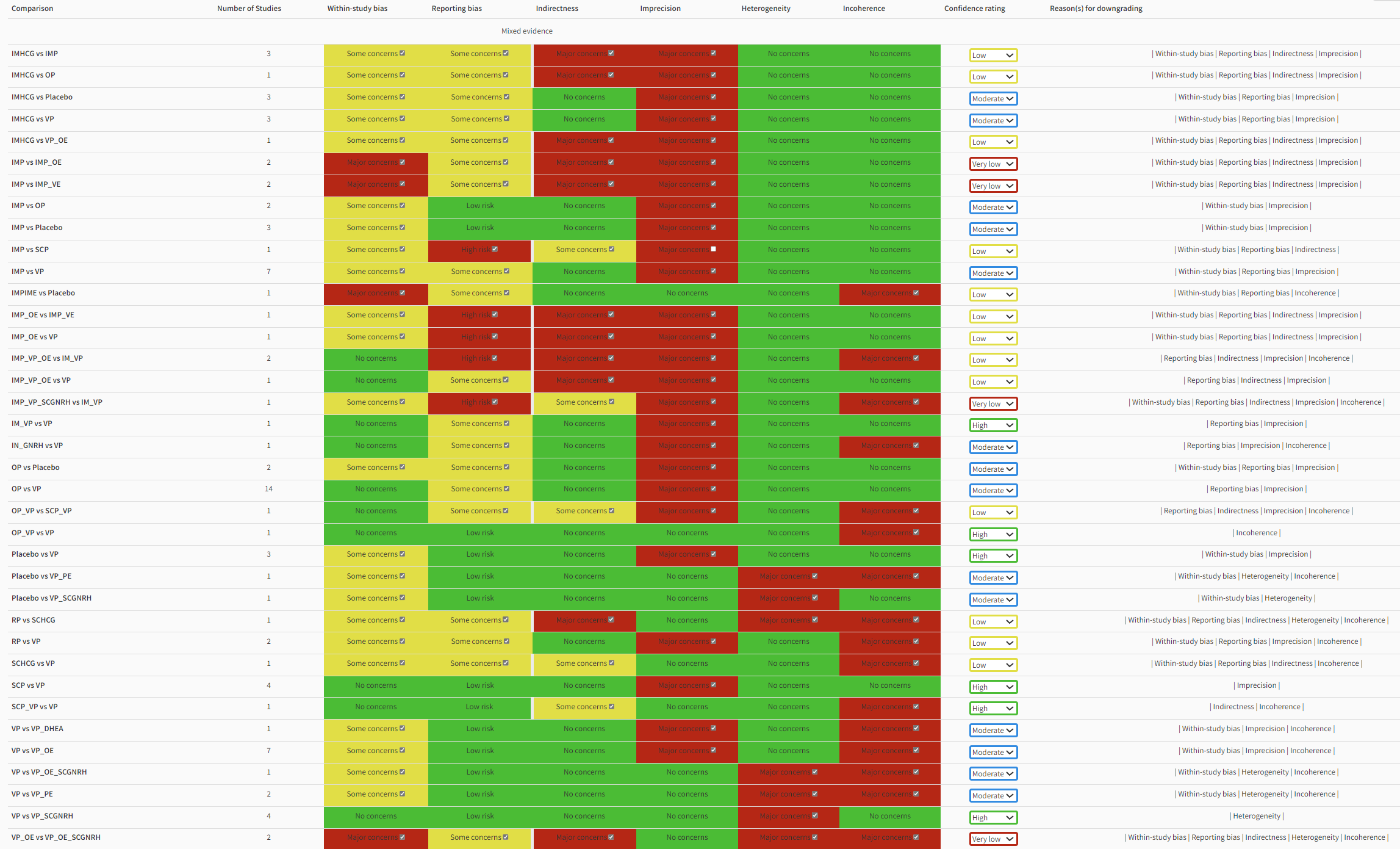


Table S5. CiNeMA Ratings; Mixed Evidence. Clinical Pregnancy.


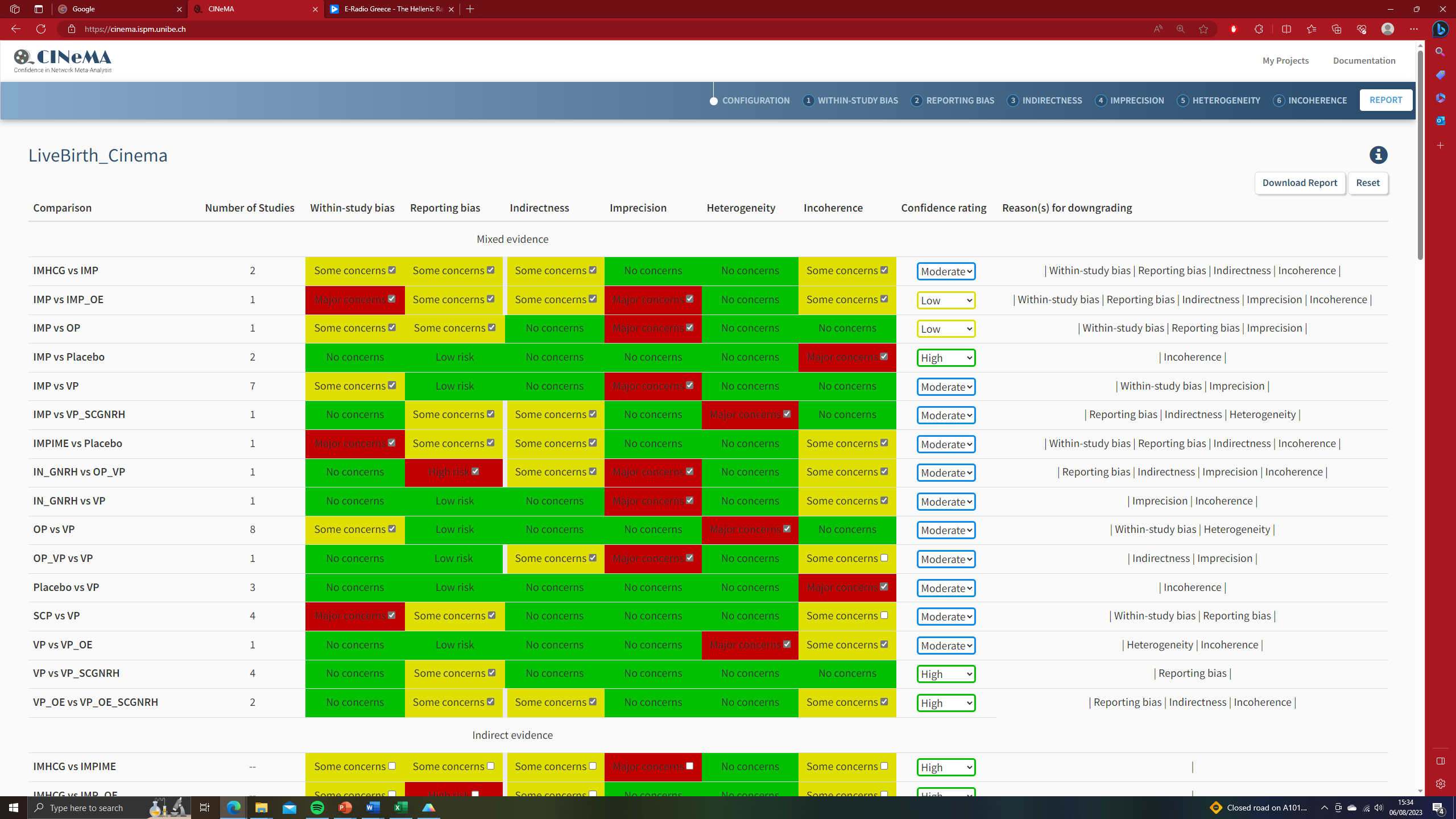
Table S6. CiNeMA Ratings; Mixed Evidence. Live Birth.


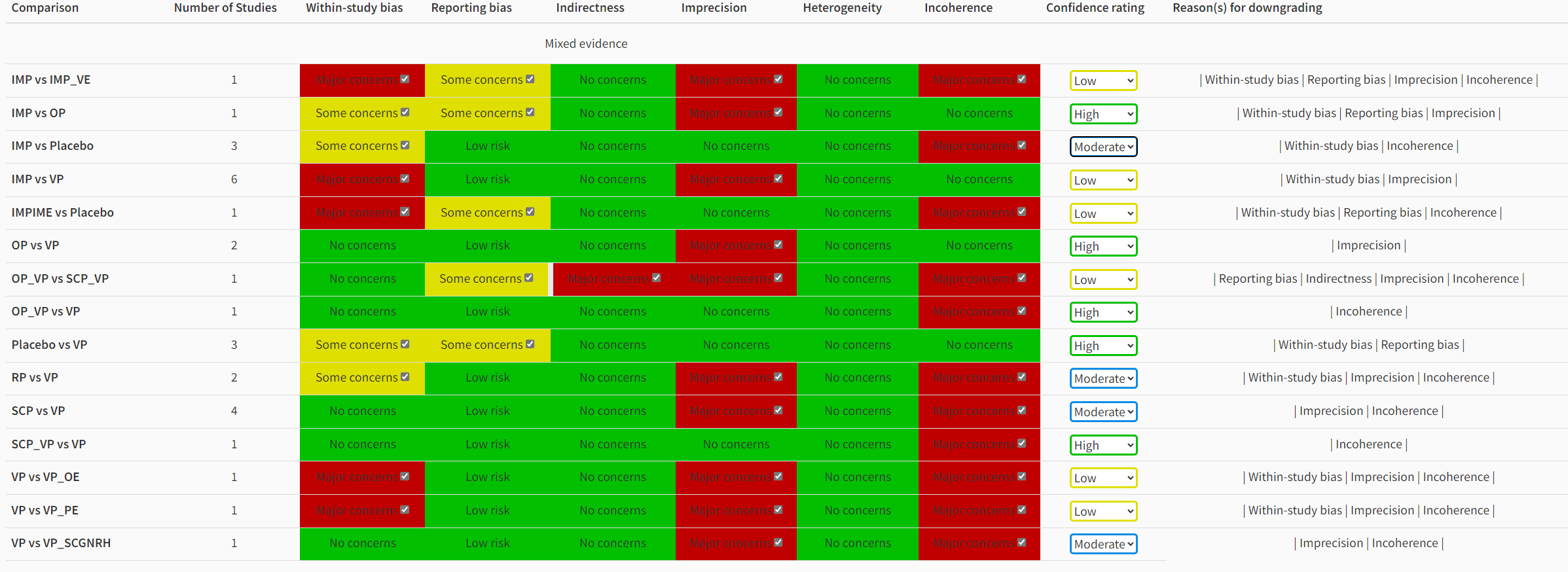
Table S7. CiNeMA Ratings; Mixed Evidence. Biochemical Pregnancy


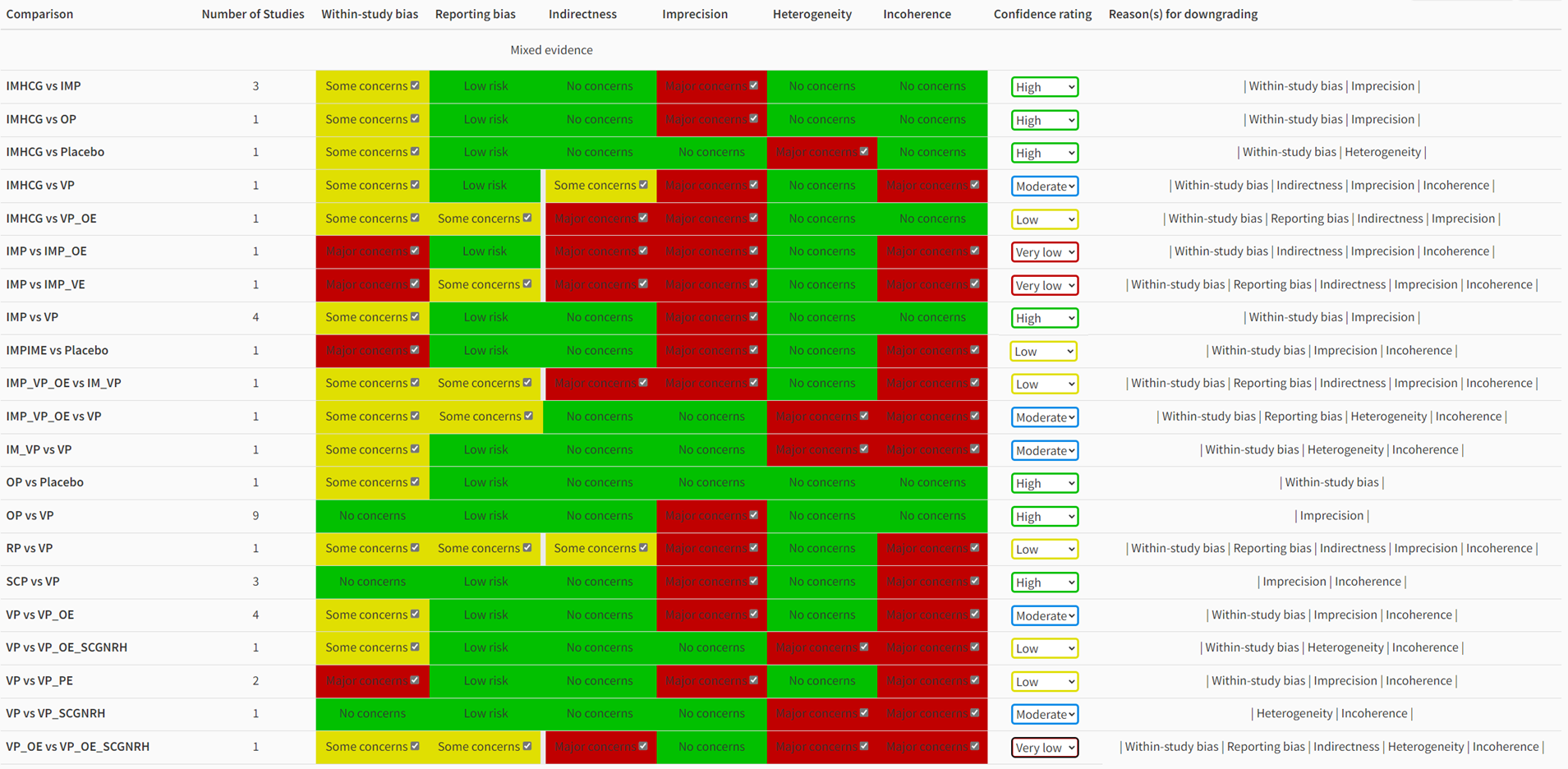


Table S8. CiNeMA Ratings; Mixed Evidence. Miscarriage.


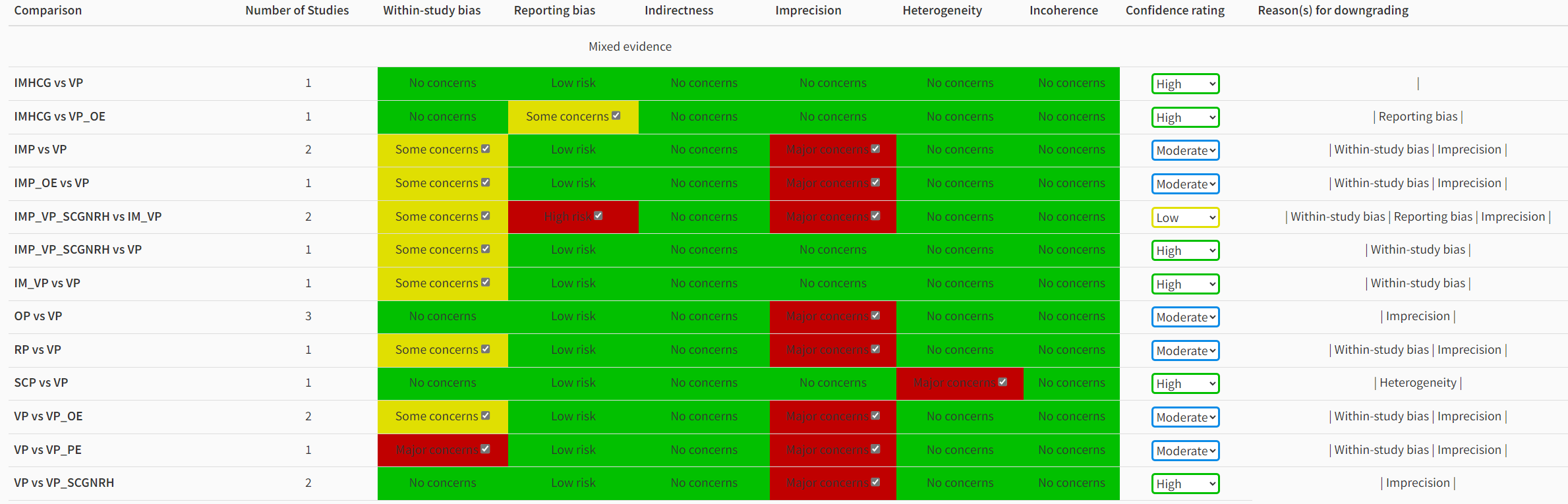
Table S9. CiNeMA Ratings; Mixed Evidence. Multiple Pregnancy.


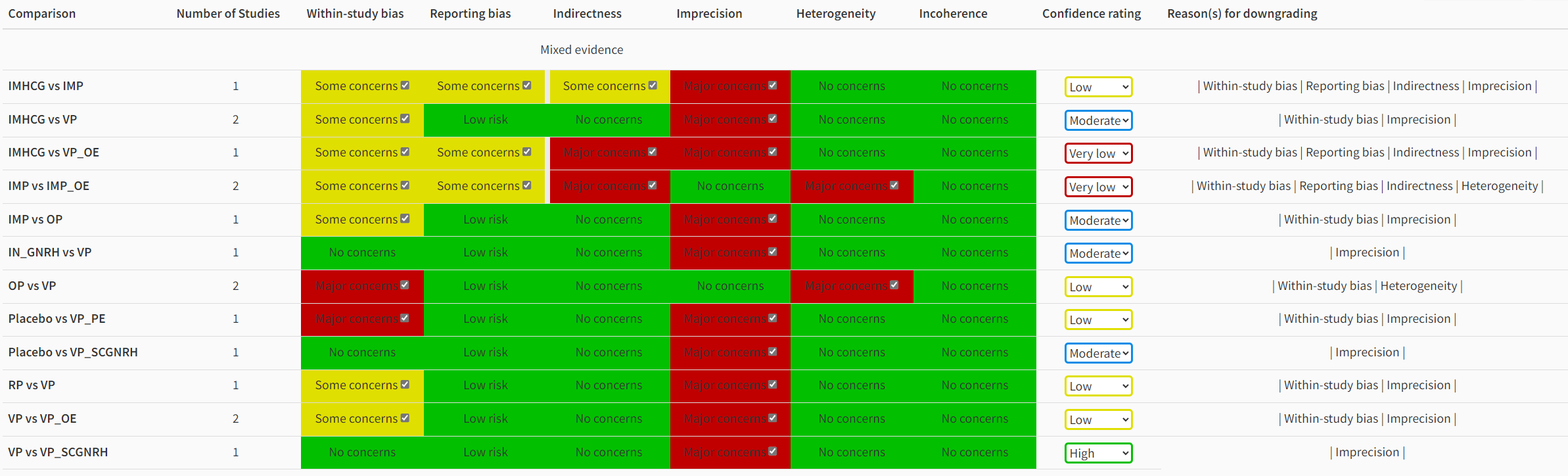
Table S10. CiNeMA Ratings; Mixed Evidence. OHSS.

| **Study** | **Treatments examined** | **Number of oocytes retrieved** | **Number of MII oocytes** | **Peak estradiol** | **Day 3 Embryo transfers (%, Population)** | **Number of embryos transferred** | **Notes** |
| --- | --- | --- | --- | --- | --- | --- | --- |
| Gawron et al., 2023 | OPPVP (21) vs. SCPPVP (22) | Not calculable | Not calculable | Not stated | 0% | Not calculable |  |
| Kao et al., 2022 | SCP (2) vs. PVP (3) | (2) 11.5 ±1.3; (3) 12.1 ± 1.5 | Not stated | Not stated | Not stated | (2) 2.6 ±0.3; (3) 2.7 ± 0.2 |  |
| Razieh et al., 2009 | PVPSCGNRH (14) vs Placebo (1) | (14) 5.42 ± 2.54; (1) 5.89 ± 2.81 | Not stated | Not stated | 100% | (14) 2.25 ± 0.78; (1) 2.4 ±0.79 |  |
| Iwase et al., 2008 | IMP (6) vs OP (10) | (6)9.95 ± 1.02; (10) 11.9 ± 1.78 | Not stated | (6) 3,666 ± 309; (10) 3,248 3,248 ± 210 | 100% | (6) 2.47± 0.19; (10) 2.20± 0.19 |  |
| Moini et al., 2022 | SCP (2) vs PVP (3) | (2) 8.7±4.6; (3) 9.1±3.9 | (2) 7.1±3.6; (3) 8.0±3.6 | Not stated | 100 | (2) 2.0± 0.5; (3) 2.0± 0.6 |  |
| Madkour et al., 2016 | PVP (3) vs PVPOE (5) | (3) 12.33 ± 5.73 1; (5) 11.37 ± 5.56 | (3) 78.61 ± 8.21, (5) 79.13 ± 6.46 | (3) 1725.63 ± 590.96; (5) 1623.89 ± 437.53 | 100 | (3) 2.36 ± 0.34; (3) 2.41 ± 0.29 | Number of mature oocytes expressed in % |
| Kara et al., 2014 | PVP (3) vs PVPDHEA (18) | (3) 5.35 ± 3.45; (18) 5.74 ± 3.69 | (3) 3.97 ± 2.63; (18) 4.03 ± 2.40 | (3) 2052.42 ± 2399.97(18) 1632.76 ± 1945.16 | 100 | Not stated |  |
| Serna et al., 2008 | PVP (3) vs PVPPE (7) | (3) 10± 0.9; (7) 10.9± 1.1 | Not stated | (3) 2180± 172; (7) 1952± 167 | 100 | (3) 2; (7) 2 |  |
| Fatemi et al., 2006 | PVP (3) vs PVPOE (5) | (3) 12.33 ± 7.4; (5) 11.19 ± 6.15 | Not stated | Not stated | 100 | (3) 1.29 ± 0.46; (5) (5) 1.27 ± 0.45 |  |
| Kleinstein et al., 2005 | PVP (3) Tablet vs. gel (gel incl) | Not stated | Not stated | Not stated | 100 | (Both arms) 2±1 |  |
| Zegers-Hochschild et al., 2000 | PVP (3) vs. IMP (6) | Not stated | Not stated | Not stated | 100 | (3) 3.73; (6) 3.72 |  |
| Andersen et al., 2002 | PVP (3) | Not stated | Not stated | Not stated | 100 | (Both arms) 2±1 |  |
| Artini et al., 1995 | Placebo (1) vs. PVP (3) vs. IMP (6) vs IMHCG (9) | Not stated | Not stated | Not stated | 100 | Not stated |  |
| Araujo et al., 1994 | IMP (6) vs IMHCG (9) | Not stated | Not stated | Not stated | 100 | Not stated |  |
| Griesinger et al., 2018 | PVP (3) vs. OP (10) | Not stated | Not stated | Not stated | 50 | (3) 2±1; (10) 2±1 |  |
| Goudge et al., 2010 | IMP (6) | Not stated | Not stated | (6) 1,179.7 ±603.5 | 100 | (6) 2.12±0.32 |  |
| Kohls et al., 2012 | PVP (3) | (3) 10.1 ±4.8 | (3) 7.7 ±3.6 | (3) 1,526 ±728 | 100 | (3) 3.5±1.7 |  |
| Kyrou et al., 2011 | PVP (3) | (3) 11.6+6.2 | Not stated | (3) 1860.8+1176.1 | 100 | (3) 1.5+0.6 |  |
| Prietl et al., 1992 | Placebo (1) vs. IMPIME (11) | (1) 8.2±l.O; (11) 8.8 ±1.0 | Not stated | (1) 2767±314; (11) 3174±340 | 100 | (1) 2.7 ±0.1; (11) 2.9 ±0.1 |  |
| Ceyhan et al., 2007 | PVP (3) vs. PVPPE (7) | (3) 10.2±3.5; (7) 12±3.6 | (3) 6.7±3.9; (7) 8.1±3.7 | (3) 1,793.2±849.4; (7) 1,959.9±1,014.8 | 75 | (3) 2.1±1.2; (7) 1.8±1.2 |  |
| Farhi et al., 2000 | IMPVP (4) vs. IMPPVPOE (12) | (4) 14.3± 6.0; (12) 12.9 ± 7.1 | Not stated | Not stated | 100 | (4) 3.8 ± 1.1; (12) 3.7 ± 0.9 |  |
| Engmann et al., 2008 | IMP (6) vs IMPPVE (13) | (6) 13.5 ± 6.9; (13)13.1 ± 6.4 | (6) 79.9 ± 15.1; (13)78.1± 19.2 | (6) 2029 ± 931; (13) 1975 ± 871 | 100 | (6) 2.5 ± 0.7; (13)2.5± 0.8 | Number of mature oocytes expressed in % |
| Belaisch-Allart et al., 1990 | Placebo (1) vs IMHCG (9) | Not stated | Not stated | Not stated | 100 | Not stated |  |
| Kupferminc et al., 1990 | Placebo (1) vs IMHCG (9) OP (10) | Not stated | Not stated | Not stated | 100 | (10) 2.85; (1) 2.7; (9) 2.9 |  |
| Aghahosseini et al., 2011 | PVP (3) vs. PVPOE (5) | (3) 3 ±1; (5) 3 ±1 | (3) 2 ±1; (5) 2 ±1.5 | Not stated | 100 | (3) 2 ±1; (5) 2 ±1.5 |  |
| Lin et al., 2013 | IMP (6) vs. IMPOE (8) | (6)12.40 ± 4.79; (8) 11.78 ± 4.81 | Not stated | (6) 2996 ± 1167.37; (8) 2790 ± 1193.5 | 100 | (6) 2.19 ± 0.4; (8) 2..21 ± 0.51 |  |
| Yanushpolsky 2010 | PVP (3) vs. IMP (6) | (3) 16.0 ±8.0; (6) 15.4 ± 7.6 | (3) 72.8 ± 19.7; (6) 71.0 ± 21.1 | Not stated | 100 | (3) 2.1±0.7; (6) 2.2 ±0.8 |  |
| Elgindy et al., 2010 | IMP (6) vs. IMPOE (8) vs. IMPPVE (13) | (6) 14 3.8; (8) 13.19 4.13; (13) 12.98 5.9 | Not stated | Not stated | 100 | (6) 2.77 0.43; (8) 2.67 0.54; (13) 2.73 0.44 |  |
| Isik et al., 2009 | PVP (3) vs PVPSCGNRH (14) | (3) 10.07 ± 8.87; (14) 10.00 ± 7.74 | (3)7.77 ± 6.63; (14) 7.71 ± 5.57 | Not stated | 100 | (3) 2.23 ± 1.1; (14) 2.27 ± 1.15 |  |
| Yildiz et al., 2014 | PVPOE (5) vs. .PVPOESCGNRH (15) | (5) 13.2 7.1; (15) 14.7 7.0 | Not stated | Not stated | 100 | (5) 2.68 ± 0.7; (14) 2.76 ± 0.5 |  |
| Dal Prato et al., 2008 | PVP (3) vs IMP (6) | Not stated | Not stated | Not stated | 100 | Not stated |  |
| Propst et al., 2001 | PVP (3) vs IMP (6) | (3) 14.0 (10.0, 16.0); (6) 13.0 (9.0, 19.5) | Not stated | (3) 1470 (1096, 2105); (6) 1688 (1244, 2312) | 79 | (3) 4.0 (3.0, 4.0); (6) 3.0 (2.3, 4.0) |  |
| Chakravarty et al., 2005 | PVP (3) vs oP (10) | Not stated | Not stated | Not stated | 100 | Not stated |  |
| Friedler et al., 1999 | PVP (3) vs OP (10) | Not stated | Not stated | Not stated | 100 | Not stated |  |
| Pouly et al., 1996 | PVP (3) vs OP (10) | Not stated | Not stated | (3) 2352±1381; (7) 2357±1071 | 100 | (3) 2.9±0.8; (7) 3±0.9 |  |
| Salehpour et al., 2013 | PVP (3) vs OP (10) | (3) 9.68 ± 6.45; (10) 11.05 ± 5.66 | (3) 7.43 ± 5.52; (10) 8.55 ± 5.20 | Not stated | 100 | (3) 2.33 ± 0.86; (10) 2.30 ± 0.74 |  |
| Bergh et al., 2012 | PVP (3) | (3) 10.5 (5.3) | Not stated | Not stated | 100 | (3) 1.3 (0.5) |  |
| Doody et al., 2009 | PVP (3) | Not stated | Not stated | Not stated | 50 | Not stated |  |
| Tay et al., 2005 | PVP (3) vs PRP (16) SCHCG (17) | (3) 7.1 ±4.4 ; (16) 9.3 ± 4.4l (17) 8.6 ± 3.4 | Not stated | (3) 9357 ; (16)9975 (17) 5096 | 100 | (3) 2.3 ± 0.6 ; (16) 2.3 ± 0.7 (17)2.3 ± 0.5 |  |
| Abate et al., 1999 | PLACEBO (1) vs. PVP (3) vs. IMP (6) | Not stated | Not stated | Not stated | 100 | Not stated |  |
| Abate et al., 1999i | PLACEBO (1) vs IMP (6) | (1) 8.2 ±4.1(6) 8.6 ±4.9 | (1) 80%; (6) 78% | Not stated | 100 | (1) 3± 1.5; (14) 3.2 ± 1.3 |  |
| Aboulghar et al., 2015 | PVP (3) vs. PVPSCGNRH (14) | (3) 13.7 ± 5.5; (14) 13.5 ± 6.0 | Not stated | (3) 2483 ± 867; (14) 2401 ± 746 | Not stated | (3) 2.7 ± 0.6; (14) 2.6 ± 0.6 |  |
| Aghsa et al., 2012 | PVP (3) vs.PRP (16) | (3) 8.0 ± 3.1; (16) 8.2 ± 3.9 | (3) 6 ± 2.8; (16) 6.3 ± 2.8 | (3) 2,180 ± 1,001; (16) 2,210 ± 1,100 | 100 | (3) 2.2 ± 0.5; (16) 2.0 ± 0.6 |  |
| Ata et al., 2008 | PVP (3) vs. PVPSCGNRH (14) | (3) 10.2 (9.7–10.7); (14) 9.5 (9– 10) | (3) 7 (6.7–7.3); (14) 6.9 (6.5–7.3) | Not stated | 100 | Not stated |  |
| Baker et al., 2014 | SCP (2) vs. PVP (3) | (2) 16.4 ±8.9; (3) 15.7 ±8.3 | Not stated | Not stated | 41.75 | Not calculable |  |
| Ganesh et al., 2011 | PVP (3) vs OP(10) | Not stated | Not stated | Not stated | 100 | Not stated |  |
| Golan et al., 1993 | IMP (6) vs. IMHCG (9) | Not stated | Not stated | Not stated | Not stated | Not stated |  |
| Inamdar et al., 2012 | IMPVP (4) vs. IMPVPSCGNRH (19) | (4) 10.88 (10.12 to  11.63); (19) 10.54 (9.78  to 11.30) | Not stated | (4) 1606 (1504 to  1709); (19) 1571 (1482 to  1660 | 0% (All day 2) | (4) 2.54 (2.44 to  2.64); (19) 2.42 (2.31 to  2.53) |  |
| Lockwood et al., 2014 | SCP (2) vs. PVP (3) | Not stated | Not stated | Not stated | Not stated | Not stated |  |
| Martinez et al., 2000 | PVP (3) vs. IMHCG (9) | (3) 20.4 ± 5.3 ; (9) 19.3 ± 4.8 | Not stated | (3) 2132 ± 509 ; (9) 2026 ±517 | 100 | (3)3.1 ± 0.7 (9) 3.2 ± 0.8 |  |
| Patki et al., 2007 | PVP (3) vs. OP (10) | Not stated | Not stated | Not stated | Not stated | Not stated |  |
| Stadtmauer et al., 2013 | PVP (3) | Not stated | Not stated | Not stated | Not stated | Not stated |  |
| Tesarik et al., 2006 | PVPOE (5) vs. PVPOESCGNRH (15) | (3) 12.2 ± 4.5; (15) 12.4 ± 4.6 | Not stated | (5) 1733 ± 190; (15) 1745 ± 201 | 100 | (3) 2.3 ± 0.5 ; (15) 2.3 ± 0.5 |  |
| Tournaye et al., 2017 | PVP (3) VS OP (10) | Not stated | Not stated | Not stated | Not stated | (3) 2.0 ± 1; (10) 2.0 ± 1 |  |
| Michnova et al., 2017 | PVP (3) | (3) 21.3±8.8 | (3) 10.2±5.9 | (3) 10889±3645 | 100 | (3) 2.0 ± 1 |  |
| Elgindy et al., 2018 | PVPOE (5) vs.IMPOE (8) | Not stated | (5) 14.9 ± 5.2; (8) 15.6 ± 5.2 | (5) 15678.8 ± 4353.8 (8) 15590.7 ± 3964.7 | 6.80% | (5) 1.93 ± 0.5; (8) 1.9 ± 1.6 |  |
| Yang et al., 2019 | PVP (3) VS. OP(10) | Not stated | Not stated | Not stated | Not calculable (3–6 day of ET) | (3) 2.0 ± 1; (10) 2.0 ± 1 |  |
| Tomic et al., 2014 | PVP (3) VS. OP(10) | Not stated | Not stated | Not stated | Not calculable (2–5 day of ET) | Not stated |  |
| Gizzo et al., 2014 | PVP (3) VS. IMPVP (4) VS. IMPPVPOE (12) | Not stated | Not stated | Not stated | Not stated | Not stated |  |
| Kutlusoy et al., 2014 | PVP (3) VS. PVPOE (5) | Not stated | (3) 3.5 ± 2.0; (5) 4.2 ± 1.4 | (3) 988.0 ± 794.3; (5) 946.5 ± 635.5 | 100 | (3) 2.2 ± 1.2; (5) 2.5 ± 0.8 |  |
| Ozer et al., 2021 | PVP (3) VS. OP(10) | (3) 14.258.11; (10) 14.618.57 | (3) 12.036.61 (10)12.817.4 | Not stated | 0 | Not stated |  |
| Saharkhiz et al., 2016 | PVP (3) VS. OP(10) | (3) 9.68 ± 6.45; (10) 11.05 ± 5.66 | Not stated | Not stated | 100 | (3) 2.33 ± 0.86; (10) 2.30 ± 0.74 |  |
| Horowitz et al., 2021 | PLACEBO (1) vs. PVP (3) | (1) 13.8 ± 6.9; (3) 11.7 ± 4.6 | Not stated | (1) 1006 ± 218; (3) 1129 ± 442 | Not stated | (1) 2.0 ± 1; (3) 2.0 ± 1 |  |
| Belaisch-Allart et al., 1987 | PVP (3) VS. OP(10) | Not stated | Not stated | Not stated | 100 | Not stated |  |
| Chi et al., 2019 | PVP (3) VS. IMP (6) | Not stated | Not stated | Not stated | 100 | Not stated |  |
| Fusi et al., 2019 | PVP (3) VS.PVPSCGNRH (14) | (3) 8.7±4.5; (14) 9.2±5.3 | Not stated | Not stated | 100 | (3) 1.7±0.6; (14) 1.5±0.7 |  |
| Gorkemli et al., 2004 | PVP (3) vs. PVPPE (7) | (3) .10.3±4.3; (7) 9.6±1.5 | Not stated | (3) 1899±1345; (7) 1695±1226 | 100 | (3)3.4±2; (7) 3.6±1.9 |  |
| Ibrahem et al., 2019 | PVP (3) vs OP (10) | (3) 14.4 ± 6.9; (10) 14.02 ± 6.4 | Not stated | Not stated | 100 | (3) 2±1; (10) 2±1 |  |
| Kapur et al., 2018 | PVP (3) vs PVPOE (5) | Not stated | Not stated | (3) 2059.95±511.23; (5) 2028.16±480.26 | 100 | Not stated |  |
| Khrouf et al., 2017 | PVP (3) vs PRP (16) | (3) 8.15 (4.3); (16) 8.41 ± 4.5 | (3) 69.07; (16) 67.3 | Not stated | 85 | (3) 1; (16) 1.82 |  |
| Kwon et al., 2013 | PVP (3) vs PVPOE (5) | (3) 10 ± 2.1; (5)10.6 ± 2.7 | (3) 9.6 ± 2.6; (5) 9.6 ± 2.7 | Not stated | 100 | (3) 2.6 ± 0.2; (5) 2.7 ± 0.2 |  |
| Mele et al., 2020 | SCP (2) vs IMP (6) | Not stated | Not stated | Not stated | 100 | Not stated |  |
| Zargar et al., 2016 | PVP (3) vs IMP (6) vs. OP (10) | Not stated | Not stated | Not stated | 100 | Not stated |  |
| Pirard et al., 2014 | PVP (3) vs. INGNRH ( 22) | (3)10.7 ± 5.9; (22) 10.1 ± 4.1 | Not stated | (3) 2929 ± 1439; (22)2960 ± 1068 | 100 | (3) 1.4 ± 0.5; (22) 1.4 ± 0.5 |  |
| Turgut Var et al., 2011 | PVP (3) vs. PVPOE (5) vs. IMHCG (9) | (3)9.5 ± 1.8; (9)9.5 ± 3.9; (5) 9.1 ± 3.9 | Not stated | (3) 2,495.5 ± 309.5; (9) 2,338.4 ± 474.1; (5) 2,414.6 ± 620.5 | 100 | (3) 2.7 ± 0.4; (5) 2.7 ± 1.0 (9) 2.6 ± 0.6 |  |
| Humaidan et al., 2021 | PVP (3) vs. SCHCG (17) | (3) 7 (5–10); (17) 9 (7-11) | (3) 6 (4–8); (17) 7 (5-10) | Not stated | Not stated | (3) 1 (1–2); (17) 2 (1–3) |  |

Table S11. Characteristics of embryo transfers, oocytes retrieved, number of mature oocytes, number of embryos transferred and peak estradiol levels

| **Treatment** | **Clinical Pregnancy (OR, 95CrI)** | **Clinical Pregnancy (aOR, 95CrI)** | **Live Birth (OR, 95CrI)** | **Live Birth (aOR, 95CrI)** | **Biochemical Pregnancy (OR, 95CrI)** | **Biochemical Pregnancy (aOR, 95CrI)** | **Miscarriage (OR, 95CrI)** | **Miscarriage (aOR, 95CrI)** | **Multiple Pregnancy (OR, 95CrI)** | **Multiple Pregnancy (aOR, 95CrI)** | **OHSS (OR, 95CrI)** | **OHSS (aOR, 95CrI)** |
| --- | --- | --- | --- | --- | --- | --- | --- | --- | --- | --- | --- | --- |
| IM_VP | 0.86 [0.52, 1.45) |  |  |  |  |  | 3.99 (1.95, 8.56) | 3.92 (1.95, 5.78) | 7.09 (2.49, 31.1) | 7.11 (2.49, 12.78) |  |  |
| IMHCG | 0.89 (0.68, 1.15) | 0.90 (0.69, 1.16) | 9.67 (2.34, 73.2) | 9.71 (2.34, 37.8) |  |  | 0.88 (0.44, 1.74) | 0.88 (0.44, 1.74) | 26.3 (5.89, 178.00) | 26.1(6.78, 59.10) | 1.64 (0.75, 3.71) | 1.64 (0.75, 3.70) |
| IMP | 1.03 (0.88, 1.19) | 1.022 (0.83, 1.11) | 1.07 (0.88, 1.30) | 1.06 (0.81, 1.29) | 1.21 (0.97, 1.51) | 1.20 (0.96, 1.49) | 1.28 (0.93, 1.75) | 1.28 (0.93, 1.75) | 0.87 (0.71,1.07) | 0.87 (0.71,1.07) | N/A | N/A |
| IMP_OE | 0.85 (0.63, 1.16) | 0.85 (0.63, 1.16) | 0.77 (0.50, 1.20) | 0.74 (0.39, 1.13) |  |  | 1.47 (0.63, 3.44) | 1.48 (0.71, 3.54) | 0.85 (0.44, 1.61) | 0.85 (0.44, 1.61) |  |  |
| IMP_VE | 1.05 (0.68, 1.61) | 1.02 (0.59, 1.49) |  |  | 1.11 (0.54, 2.26) | 1.10 (0.53, 2.16) | 1.85 (0.79, 4.43) | 1.86 (0.81, 4.41) |  |  |  |  |
| IMP_VP_OE | 1.23 (0.75, 2.03) |  |  |  |  |  | 2.36 (1.05, 5.36) | 2.38 (1.18, 5.42) |  |  |  |  |
| IMP_VP_SCGNRH | 0.82 (0.43, 1.56) | 0.83 (0.42, 1.52) |  |  |  |  |  |  | 6.88 (2.42, 30.4) | 6.86 (2.42, 11.65) |  |  |
| IMPIME | 2.68 (1.06, 7.72) | 2.68 (1.06, 7.72) | 1.36 (0.33, 5.51) | 1.32 (0.31, 3.43) | 0.008 (0.0003, 0.05) | 0.008 (0.0003, 0.03) | 0.08 (0.01, 0.46) | 0.08 (0.01, 0.46) |  |  |  |  |
| INGNRH | 1.86 (0.45, 10) | 1.84 (0.45, 8.2) |  |  |  |  |  |  |  |  | N/A | N/A |
| OP | 0.98 (0.90, 1.07) | 0.98 (0.91, 1.06) | 1.19 (1.07, 1.34) | 1.18 (1.07, 1.31) | 1.45 (0.73, 2.91) | 1.45 (0.73, 2.89) | 0.94 (0.70, 1.24) | 0.91 (0.70, 1.24) | 1.11 (0.86, 1.43) | 1.11 (0.85, 1.42) | 1.87 (1.15, 3.04) | 1.87 (1.15, 3.01) |
| OP_VP | 0.09 (0.04, 0.21) |  | 0.64 (0.20, 2.29) | 0.63 (0.21, 2.27) | 0.07 (0.02, 0.16) | 0.07 (0.02, 0.16) |  |  |  |  |  |  |
| Placebo | 0.53 (0.41, 0.68) | 0.49 (0.39, 0.67) | 0.22 (0.08, 0.53) | 0.21 (0.05, 0.54) | 0.22 (0.12, 0.38) | 0.22 (0.12, 0.35) |  |  |  |  | N/A | N/A |
| RP | 1.34 (0.75, 2.36) | 1.31 (0.69, 2.76) |  |  | 0.77 (0.48, 1.23) | 0.75 (0.48, 1.21) | 1.04 (0.27, 4.03) | 1.04 (0.27, 4.01) | 0.65 (0.07, 4.42) | 0.65 (0.07, 4.42) | N/A | N/A |
| SCHCG | 6.42 (1.86, 31.5) | 6.57 (1.81, 13.45) |  |  |  |  |  |  |  |  |  |  |
| SCP_VP | 0.045 (0.01, 0.13) |  |  |  | 0.113 (0.04, 0.25) | 0.11 (0.03, 0.24) |  |  |  |  |  |  |
| VP_DHEA | 0.95 (0.53, 1.72) |  |  |  |  |  |  |  |  |  |  |  |
| VP_OE | 1.16 (0.90, 1.49) | 1.16 (0.90, 1.49) | 4.57 (1.26, 20) | 4.49 (1.25, 7.8) | 1.18 (0.46, 3.04) | 1.18 (0.47, 3.02) | 1.17 (0.69, 2.02) | 1.17 (0.69, 2.02) | 3.17 (0.94, 13.1) | 3.16 (0.94, 9.81) | N/A | N/A |
| VP_OE_SCGNRH | 1.57 (1.11, 2.22) | 1.64 (1.18, 2.29) | 8.81 (2.35, 39.1) | 8.82 (2.36, 17.8) |  |  | 3.94 (1.69, 10.1) | 3.94 (1.69, 10.1) |  |  |  |  |
| VP_PE | 1.73 (1.16, 2.58) | 1.73 (1.16, 2.58) |  |  | 0.95 (0.51, 1.76) | 0.93 (0.50, 1.73) | 2.97 (1.14, 8.93) | 2.97 (1.14, 6.78) | 1.65 (1.02, 266) | 1.65 (1.02, 266) | N/A | N/A |
| VP_SCGNRH | 1.28 (1.05, 1.55) |  | 1.76 (1.45, 2.15) | 1.76 (1.45, 2.15) | 1.91 (0.97, 3.76) | 1.91 (0.96, 3.76) |  |  |  |  | N/A | N/A |
| SCP |  |  | 0.51 (0.44, 0.59) | 0.51 (0.43, 0.57) | 0.93 (0.97, 3.76) | 0.93 (0.97, 3.75) | 0.85 (0.37, 0.80) | 0.85 (0.37, 0.80) | 0.09 (0.009, 0.55) | 0.09 (0.009, 0.55) |  |  |

Table S12. Unadjusted and multilevel network meta‐regression odds ration and 95% CrI per outcome. Variables adjusted for were number of oocytes retrieved, number of MII oocytes, peak estradiol, number of embryos transferred (Table S11).
